# Nonequilibrium Formation of C_4_ Carbonyls in Interstellar Ices Linked to Amino Acids

**DOI:** 10.1021/acscentsci.6c00923

**Published:** 2026-09-02

**Authors:** Zesen Wang, Jia Wang, Anastasia S. Shishova, Sergey O. Tuchin, Mason McAnally, Andrew M. Turner, Niki Jabari, Ralf I. Kaiser

**Affiliations:** † W. M. Keck Research Laboratory in Astrochemistry, University of Hawaii at Manoa, Honolulu, Hawaii 96822, United States; ‡ Department of Chemistry, University of Hawaii at Manoa, Honolulu, Hawaii 96822, United States; § Samara National Research University, Samara 443086, Russia

## Abstract

Complex organic molecules in interstellar ices and carbonaceous asteroids record key steps in the chemical evolution toward life, yet the origin of branched carbonyl compounds—a fundamental class of biorelevant molecules—remains unresolved. Here, we demonstrate the efficient formation of C_4_ carbonyls, isobutyraldehyde ((CH_3_)_2_CHCHO) and 2-butanone (CH_3_CH_2_COCH_3_) via barrierless radical–radical recombination in carbon monoxide–propane (CO–C_3_H_8_; 1:1.1 ± 0.2) and ethane–acetaldehyde (C_2_H_6_–CH3CHO; 1.5 ± 0.3:1) ice mixtures at 5 K irradiated with 5 keV electrons as proxies for secondary electrons generated by galactic cosmic rays. Isobutyraldehyde arises from recombination of formyl (HĊO) and isopropyl (CH_3_ĊHCH_3_) radicals, whereas 2-butanone forms through acetyl (CH_3_ĊO) and ethyl (CH_3_ĊH_2_) radical coupling. Using isomer-selective photoionization mass spectrometry with isotopic labeling, we provided strong evidence for these products together with the enol 2-methylprop-1-en-1-ol ((CH_3_)_2_CCHOH) in the gas phase. These results establish a plausible low-temperature mechanism for molecular mass growth that generates branched carbon skeletons without activation barriers, bridging a critical gap between simple interstellar species and structurally complex, biorelevant organics. The demonstrated pathways operate under cosmic-ray-driven, nonequilibrium chemistry in icy grains, providing a plausible route to C_4_ backbone motifs found in prebiotic molecules, including amino acids and fatty acids. Our findings show that chemical complexity—including carbon skeleton branching—can emerge in deep space prior to planetary accretion, implying a plausible extraterrestrial origin for key molecular precursors delivered to early Earth and exo planetary systems.

## Graphical abstract

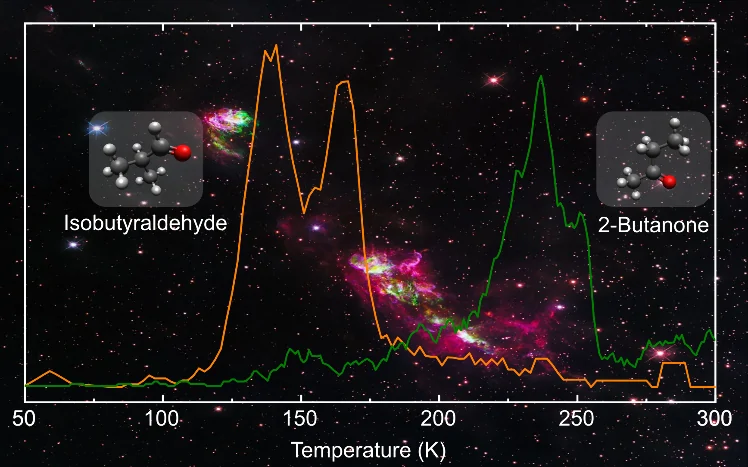

## Introduction

Since the first detection of formaldehyde (H_2_CO, **1**)^1^ and acetone (CH_3_COCH_3_, **2**)^2^ in the interstellar medium (ISM), aldehydes (RCHO) and ketones (RCOR′) have attracted extensive attention from the astronomy,^3–5^ astrochemistry,^6–8^ astrobiology,^9–11^ and laboratory astrophysics^12–16^ communities due to their roles as key intermediates in molecular mass growth toward biorelevant molecules linked to the origins of life.^9,10,16–18^ To date, 12 aldehydes and three ketones, including **1**, **2**, acetaldehyde (CH_3_CHO, **3**),^19–21^ propanal (CH_3_CH_2_CHO, **4**),^22^ and hydroxyacetone (CH_3_COCH_2_OH, **5**)^23^ have been detected in the interstellar medium (Figure S1). In addition, carbonaceous chondrites harbor not only **1**–**4** but also more complex aldehydes and ketones including butyraldehyde (CH_3_CH_2_CH_2_CHO, **6**),^24–27^ isobutyraldehyde ((CH_3_)_2_CHCHO, **7**),^24^ and 2-butanone (CH_3_CH_2_COCH_3_, **8**).^24,25,27^

These findings indicate that these organics form in extraterrestrial environments, survive incorporation into comets, asteroids, and planetesimals, and may ultimately be delivered to planets such as early Earth, providing an exogenous source of prebiotic matter.^28,29^ However, despite their importance as precursors to astrobiologically relevant molecules such as amino acids and fatty acids, the fundamental formation mechanisms of complex carbonyl compounds in interstellar environments remain largely unresolved in particular for the biorelevant species **7** and **8**, which provide direct entry into amino acid precursor chemistry via the Strecker synthesis.^30–34^

In prebiotic chemistry, **7** and **8** may react with ammonia (NH_3_) to form the corresponding imines, isobutylimine ((CH_3_)_2_CHCHNH, **9**) and 2-butanimine (CH_3_CH_2_C­(NH)­CH_3_, **10**) (Figure 1). **9** and **10** could then undergo hydrogen cyanide (HCN) addition to valinonitrile ((CH_3_)_2_CHCH (NH_2_)­CN, **11**) and isovalinonitrile (CH_3_CH_2_C­(CH_3_)­(NH_2_)­CN, **12**). Hydrolysis of **11** and **12** could yield the proteinogenic amino acid valine ((CH_3_)_2_CHCH­(NH_2_)­COOH, **13**) and the nonproteinogenic amino acid isovaline (CH_3_CH_2_C­(CH_3_)­(NH_2_)­COOH, **14**). Valine (**13**) is essential for protein structure and metabolic regulation,^35,36^ while isovaline (**14**) represents a precursor to branched-chain amino acid and a marker of prebiotic chemistry.^37–39^ Beyond amino-acid-related chemistry, oxidation of **7** could result in the formation of isobutyric acid ((CH_3_)_2_CHCOOH, **15**), a precursor to branched-chain fatty acids.^40^ Carboxylation of **8** may lead to levulinic acid (CH_3_COCH_2_CH_2_COOH, **16**), linking this ketone to a broader inventory of prebiotically relevant keto acids.^41^ Notably, **13**–**16** have been identified in carbonaceous meteorites such as Murchison.^39,42–44^ Consequently, **7** and **8** may serve as key precursors to keto acids, fatty acids, and amino acids (Figure 1), and may contribute to inventories of prebiotic precursors. Elucidating their interstellar formation is therefore critical for unraveling fundamental abiotic synthesis routes of a key class of complex organic molecules (COMs) in deep space: aldehydes and ketones.

**Figure 1:**
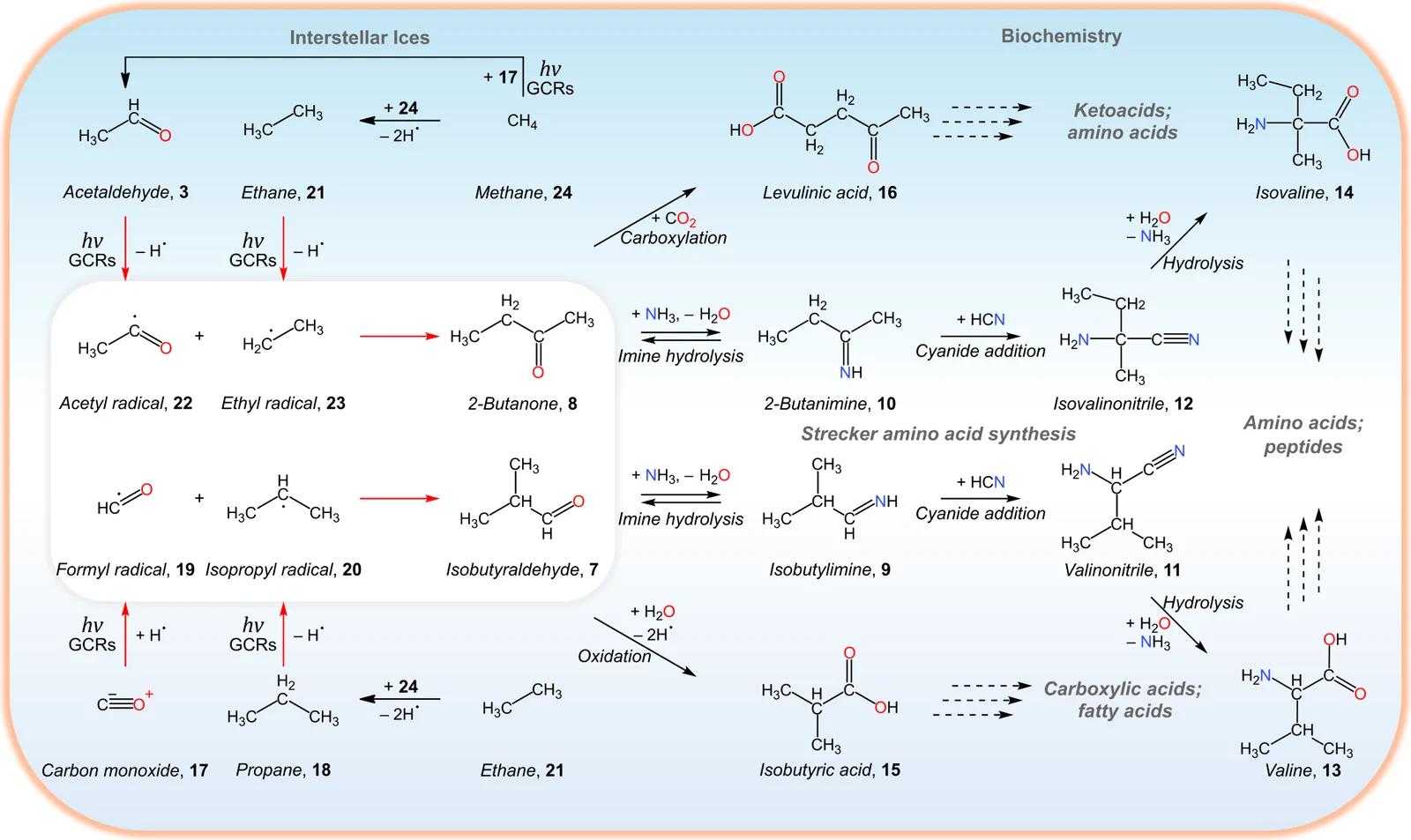
Schematic formation pathways of isobutyraldehyde ((CH_3_)_2_CHCHO, **7**) and 2-butanone (CH_3_CH_2_COCH_3_, **8**) in interstellar ices and their potential role as precursors to prebiotic molecules. The boxed region highlights the reaction network investigated in this work. Exposure of interstellar ice analogues carrying carbon monoxide (**17**) and propane (**18**) as well as acetaldehyde (**3**) and ethane (**21**) to proxies of galactic cosmic rays initiates a complex set of radical–radical reactions of formyl (HĊO, **19**) with isopropyl ((CH_3_)_2_ĊH, **20**) and acetyl (CH_3_ĊO, **22**) with ethyl (CH_3_ĊH_2_, **23**) yielding isobutyraldehyde (**7**) and 2-butanone (**8**), respectively. **7** and **8** serve as fundamental precursors to isobutyric acid (**15**) and levulinic acid (**16**), respectively, contributing to the formation of amino acids such as valine (**13**) and isovaline (**14**) as well as carboxylic and keto acids.

Here, we report the first preparation of isobutyraldehyde ((CH_3_)_2_CHCHO, **7**) and 2-butanone (CH_3_CH_2_COCH_3_, **8**) in interstellar ice analogues exposed to proxies of galactic cosmic rays (GCRs). The formation of **7** is accomplished in low temperature (5 K) carbon monoxide (CO, **17**)–propane (C_3_H_8_, **18**) interstellar analogue ices through barrierless recombination of formyl (HĊO, **19**) with isopropyl (CH_3_ĊHCH_3_, **20**) radicals (Figures 1 and 2), whereas **8** is produced in low temperature acetaldehyde (CH_3_CHO, **3**)–ethane (C_2_H_6_, **21**) ice via barrierless carbon–carbon bond coupling through recombination of acetyl (CH_3_ĊO, **22**) and ethyl (CH_3_ĊH_2_, **23**) radicals (Figures 1 and 3). Carbon monoxide (**17**) is among the most abundant constituents of interstellar ices with an abundance of up to 55% relative to water.^14,45,46^ Propane (**18**) and ethane (**21**) form in methane (CH_4_, **24**)-rich interstellar ices through irradiation-driven radical chemistry.^47,48^ Acetaldehyde (**3**) has been detected in diverse extraterrestrial environments, including cold molecular clouds,^49^ star-forming regions,^20,50^ meteorites^51^ and comets,^52^ and has also been reported in interstellar ices with upper limits of 10% relative to water.^53^ Utilizing tunable vacuum ultraviolet (VUV) photoionization reflectron time-of-flight mass spectrometry (PI-ReToF-MS) together with isotopic substitution studies, **7** and its enol 2-methylprop-1-en-1-ol ((CH_3_)_2_CCHOH, **25**), as well as **8**, were identified isomer-selectively during the temperature-programmed desorption (TPD) of processed ices. These findings unravel nonequilibrium reaction pathways leading to **7** and **8** in interstellar ices thus advancing our fundamental understanding of molecular mass growth to complex aldehydes and ketones under astrophysical conditions. This work provides a mechanistic framework between ice chemistry and the molecular foundations of life, highlighting a plausible extraterrestrial pathway for the formation of biologically relevant organics that could be available to early Earth and exoplanetary bodies.

**Figure 2:**
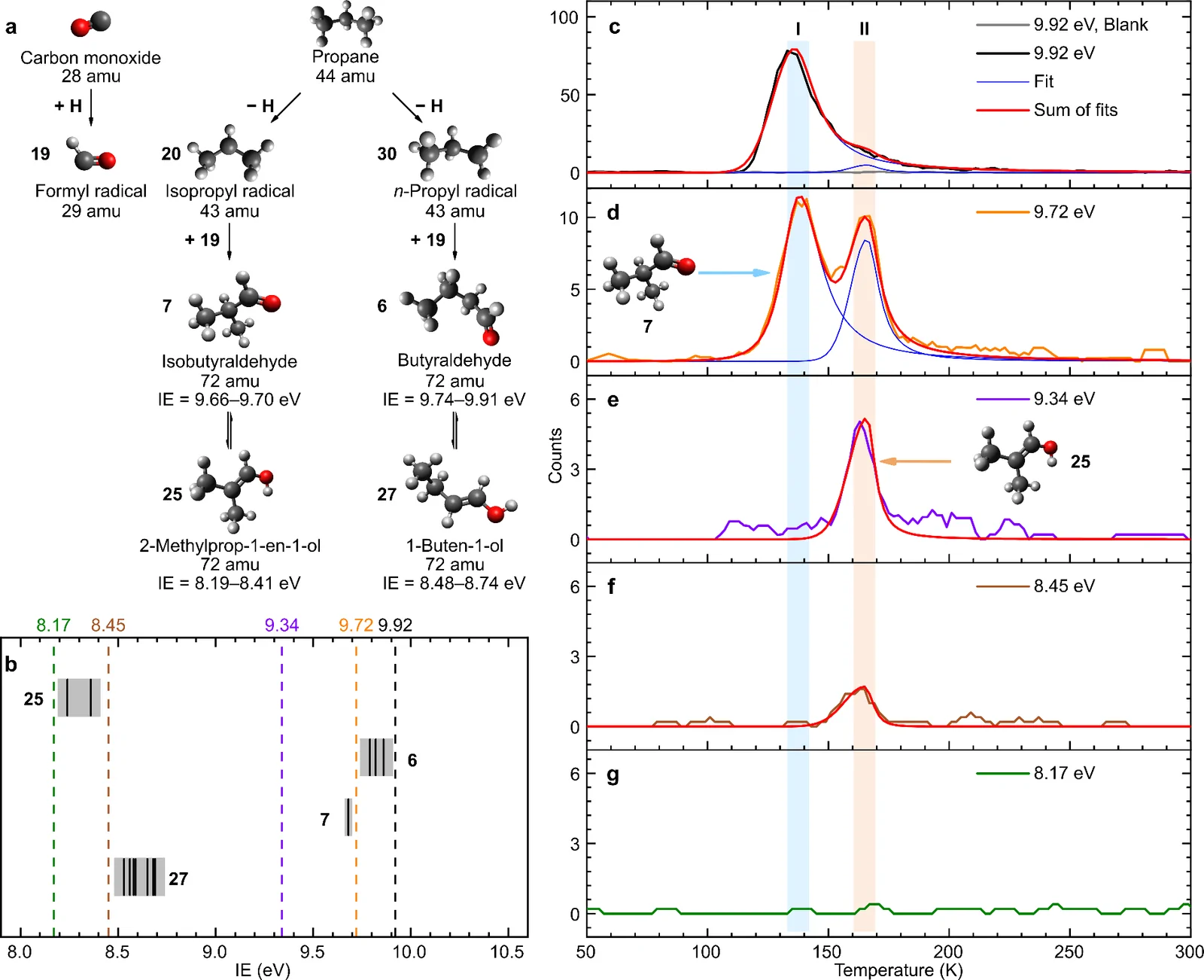
(a) Proposed formation pathways of four C_4_H_8_O (*m*/*z* = 72) isomers in irradiated CO–C_3_H_8_ ices. Barrierless radical–radical reactions produce **6** and **7**, followed by tautomerization to enols **27** and **25**. The computed adiabatic ionization energies (IEs; black solid line) and ranges of their conformers (gray area) are compiled (b). Dashed lines indicate the VUV photon energies used for photoionization. TPD profiles of *m*/*z* = 72 in CO–C_3_H_8_ ices recorded at 9.92 eV (c), 9.72 eV (d), 9.34 eV (e), 8.45 eV (f) and 8.17 eV (g). The TPD profiles are fitted with two peaks (I and II), highlighted by the shaded regions. Peaks I and II are assigned to isomers **7** and **25**, respectively. The red lines represent the overall fits.

**Figure 3:**
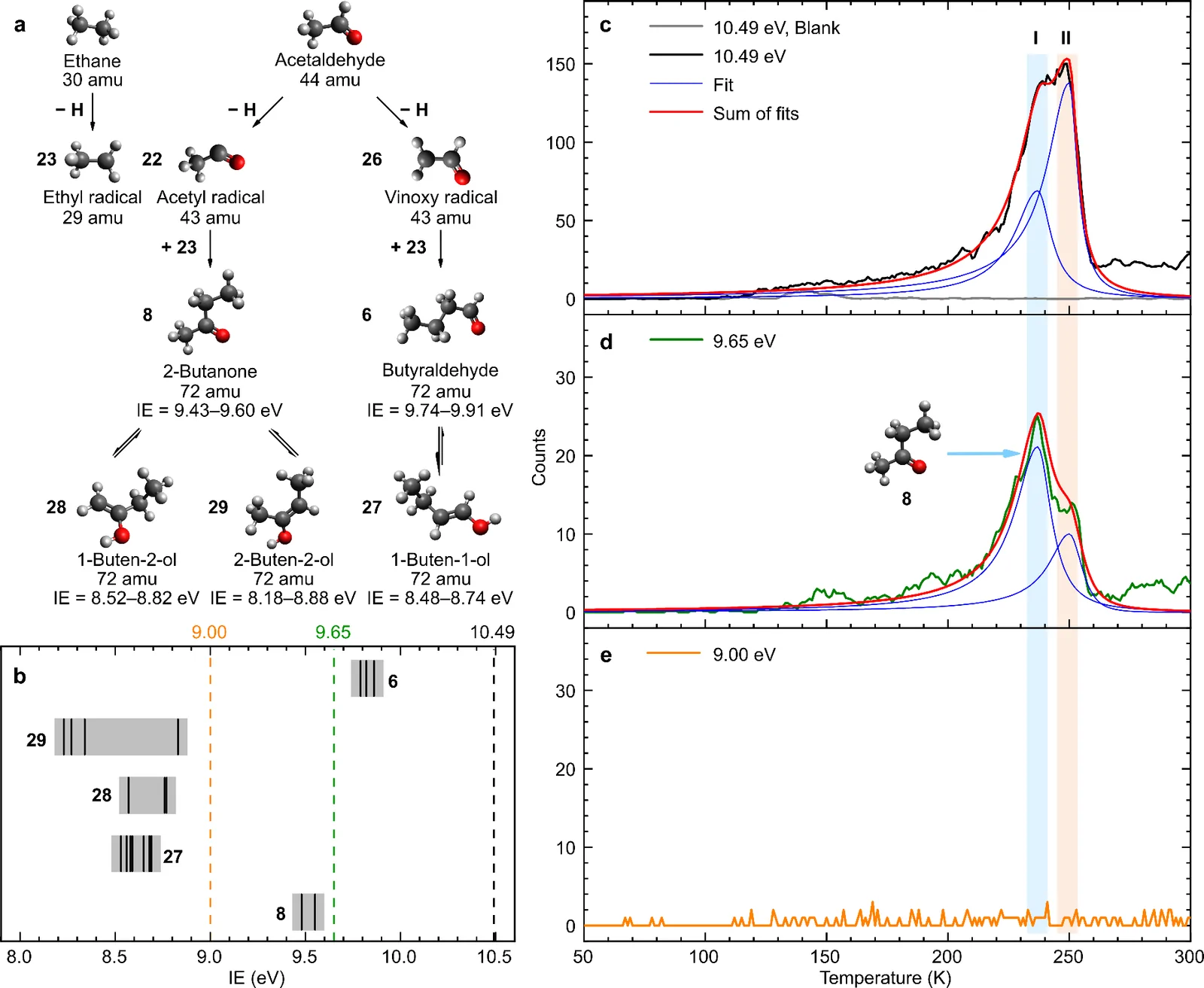
(a) Proposed formation pathways of five C_4_H_8_O (*m*/*z* = 72) isomers in irradiated C_2_H_6_–CH_3_CHO ices. Barrierless radical–radical reactions produce **6** and **8**, followed by tautomerization to enols **27**, **28**, and **29**. The computed adiabatic ionization energies (IEs; black solid line) and ranges of their conformers (gray area) are compiled (b). Dashed lines indicate the VUV photon energies used for photoionization. TPD profiles of *m*/*z* = 72 in C_2_H_6_–CH_3_CHO ices recorded at 10.49 eV (c), 9.65 eV (d), and 9.00 eV (e) are shown. The TPD profiles are fitted with two peaks (I and II), highlighted by the shaded regions. Peak I is assigned to isomer **8**. The red lines represent the overall fits.

## Results

### Fourier Transform Infrared Spectroscopy

Fourier transform infrared (FTIR) spectroscopy was employed to monitor the chemical evolution of the carbon monoxide (CO)–propane (C_3_H_8_), ethane (C_2_H_6_)–acetaldehyde (CH_3_CHO) ices along with isotopically labeled ethane (C_2_H_6_)–acetaldehyde-*d*_3_ (CD_3_CHO) systems before, during, and after exposure to ionizing irradiation (Figures S2–S5). Detailed assignments of the absorptions are provided in Tables S1–S4. In the pristine CO–C_3_H_8_ ice, the infrared absorptions are dominated by the overtone and fundamental modes of carbon monoxide such as 2ν­(CO) at 4246 cm^–1^ and ν­(CO) at 2135 cm^–1^,^54^ together with the fundamentals and combination bands of propane (Figure S2).^55–57^ In the unprocessed C_2_H_6_–CH_3_CHO and isotopically labeled C_2_H_6_–CD_3_CHO ices, the spectra can be assigned to the vibrational fundamentals and combination modes of ethane (C_2_H_6_) and acetaldehyde (CH_3_CHO/CD_3_CHO), such as CH_3_ stretching modes of C_2_H_6_ at 2973 cm^–1^ (ν_10_), 2956 cm^–1^ (ν_1_), and 2880 cm^–1^ (ν_5_)^58–60^ and the C=O stretching mode of CH_3_CHO/CD_3_CHO at 1723 cm^–1^ (ν_4_)^61,62^ (Figures S3–S5).

Following the low dose irradiation simulating (8 ± 2) × 10^5^ years of GCR exposure in cold molecular clouds, several new absorption features emerged; these were deconvoluted into multiple Gaussian peaks.^63,64^ In the irradiated CO–C_3_H_8_ ices, a new absorption at 1796 cm^–1^ is assigned to the formyl radical (HĊO, **19**, ν_3_)^65^ (Figure S2 and Table S1), whereas the exposed C_2_H_6_–CH_3_CHO ices reveal new absorptions at 2130 cm^–1^, 1839 cm^–1^, and 1570 cm^–1^; the former two are linked to carbon monoxide (ν­(CO)) and the acetyl radical (CH_3_ĊO, **22**, ν_3_),^54,66,67^ while 1570 cm^–1^ is tentatively assigned to the vinoxy radical (ĊH_2_CHO, **26**, ν_4_)^67,68^ (Figure S3 and Table S2). In the irradiated isotopically labeled C_2_H_6_–CD_3_CHO ices, absorptions at 2137 and 1849 cm^–1^ are attributed to carbon monoxide (ν­(CO)) and **22-** *d*_3_ (ν_3_(CD_3_ĊO)), while the new absorption at 2096 cm^–1^ is assigned to the ketene-*d*_2_ (ν_2_(CD_2_CO))^54,65,66,69^ (Figure S5 and Table S4).

At a higher irradiation dose, which mimics (6 ± 2) × 10^7^ years in cold molecular clouds, the spectra of the C_2_H_6_–CH_3_CHO system are more complex, revealing a broader product inventory (Figure S4 and Table S3). New absorptions at 4496 and 1301 cm^–1^ can be attributed to combination (ν_2_ + ν_3_) and fundamental (ν_4_) modes of methane,^60^ while absorptions at 3092 and 954 cm^–1^, 2338 cm^–1^, and 2133 cm^–1^, correspond to ethylene (C_2_H_4_), carbon dioxide (CO_2_), and carbon monoxide (CO), respectively.^60,70^ Additionally, absorptions at 3314 cm^–1^ and 3213 cm^–1^ are linked to acetylene (C_2_H_2_).^60^ These additional products indicate that high dose irradiation promotes secondary decomposition and sequential reaction chemistry beyond the first-generation radical formation observed under low dose conditions. While FTIR spectroscopy reveals the formation of radical intermediates and small products in the irradiated ices, the C_4_ carbonyls such as **7** and **8** cannot be uniquely identified via the infrared data alone: their absorptions are expected to overlap with those of reactants and a complex suite of products carrying the same carbonyl functional group.^71,72^ Therefore, an alternative, isomer-selective technique is clearly required to identify individual reaction products formed in these ices.

### Photoionization Reflectron Time-of-Flight Mass Spectrometry

The photoionization reflectron time-of-flight mass spectrometry (PI-ReToF-MS) technique was utilized to identify individual C_4_H_8_O isomers formed in electron-irradiated ices during TPD based on their desorption profiles, isotope shifts, and adiabatic ionization energies (IEs).^72,73^ PI-ReToF data of the irradiated carbon monoxide (**17**)–propane (**18**) ices during TPD are compiled in Figure 2 and Figure S6. Recall that the ices were studied at a low dose equivalent of cold molecular cloud galactic cosmic ray exposure of only (8 ± 2) × 10^5^ yr to minimize sequential reactions,^74^ thereby limiting the formation pathways to first-generation products.

In the CO–C_3_H_8_ system, five photon energies of 9.92, 9.72, 9.34, 8.45, and 8.17 eV were selected to distinguish the C_4_H_8_O isomers **6** and **7** accessed via radical–radical recombination and enolization, i.e. 1-buten-1-ol (CH_3_CH_2_CHCHOH, **27**) and 2-methylprop-1-en-1-ol ((CH_3_)_2_CCHOH, **25)** (Figure 2). At 9.92 eV, which can ionize all isomers if present, the TPD profile of *m*/*z* = 72 reveals sublimation events (Figure 2c), which can be deconvoluted into Peaks I and II using split Pearson VII distributions.^62,75,76^ A blank experiment carried out under otherwise identical conditions, but without electron irradiation, shows no sublimation event at *m*/*z* = 72 (Figure 2c and Figure S6a), confirming that Peaks I and II are caused by radiation-induced processing of the ices. Since all isomers **6** (IE = 9.74–9.91 eV), **7** (IE = 9.66–9.70 eV),^77^ and their corresponding enols **27** (IE = 8.48–8.74 eV) and **25** (IE = 8.19–8.41 eV), can be photoionized at 9.92 eV (Figure 2 and Tables S5–S10), Peaks I and II could potentially originate from any of these isomers. Thereafter, the photon energy was lowered to 9.72 eV, at which isomer **6** (IE = 9.74–9.91 eV) is not expected to be efficiently ionized; both Peaks I and II persisted (Figure 2d), indicating that no evidence for the formation of isomer **6** can be provided. Upon reducing the photon energy to 9.34 eV, at which only the enols **25** and **27** can be ionized, Peak I disappears while Peak II remains (Figure 2e); this finding suggests that Peak I can be associated with **7**. At an even lower photon energy of 8.45 eV, where only **25** can be ionized, a weak signal of Peak II remains (Figure 2f), indicating that Peak II can be assigned to **25**. Further lowering the photon energy to 8.17 eV, no signal is detected (Figure 2g), providing further support for the formation of **25**. Additionally, the ratio of the integrated areas of Peak I at 9.92 and 9.72 eV is 7.69 ± 0.76; this finding agrees well with the ratio of 7.51 ± 1.06 derived from the reference photoionization data of **7**,^78^ providing further evidence for the formation of **7**.

The data for the C_2_H_6_–CH_3_CHO system are compiled in Figures 3 and 4 and Figure S8. VUV photons with energies of 10.49, 9.65, and 9.00 eV were employed to photoionize and distinguish the first-generation C_4_H_8_O products **6** and **8**, as well as their enols **27**, 1-buten-2-ol (CH_2_C­(OH)­CH_2_CH_3_, **28**), and 2-buten-2-ol (CH_3_C­(OH)­CHCH_3_, **29**) (Figure 3). At 10.49 eV, all isomers can be ionized (Figure 3b). The TPD profile of ion signal at *m*/*z* = 72 obtained for the C_2_H_6_–CH_3_CHO ice was deconvoluted to two split Pearson VII distributions peaking at 237 K (Peak I) and 250 K (Peak II), respectively (Figure 3c). A blank experiment of C_2_H_6_–CH_3_CHO ice was performed without electron irradiation under otherwise identical conditions; no sublimation event was observed at *m*/*z* = 72 (Figure 3c and Figure S8a), confirming that the ion signal at *m*/*z* = 72 arises from the irradiation of ices. When the photon energy was lowered to 9.65 eV, at which isomer **6** (IE = 9.74–9.91 eV) is not expected to be ionized, both Peaks I and II remain (Figure 3d). Therefore, no evidence for the formation of isomer **6** can be provided. Thereafter, the photon energy was further reduced to 9.00 eV; at this energy, enols **27** (IE = 8.48–8.74 eV), **28** (IE = 8.52–8.82 eV), and **29** (IE = 8.18–8.88 eV) can still be ionized, but not isomer **8** (IE = 9.43–9.60 eV). However, no sublimation events were observed at 9.00 eV (Figure 3e), indicating that the ion signals of Peaks I and II at 9.65 eV may be attributed to **8**, with no evidence for the formation of enols **27**–**29** in the low dose irradiation experiments. Additional support for the formation of **8** is provided by the isotopic substitution experiments in ethane–acetaldehyde-*d*_3_ (C_2_H_6_–CD_3_CHO) ices (Figure 4). According to the proposed reaction pathways in the C_2_H_6_–CD_3_CHO ice (Figure S9), isomer **6** shifts to *m*/*z* = 74 (**6**-*d*_2_), whereas isomer **8** shifts to *m*/*z* = 75 (**8**-*d*_3_). At 10.49 eV, the TPD profiles at *m*/*z* = 74 and 75 exhibit Peak I (Figure 4a). Since both **8**-*d*_3_ and **6**-*d*_2_ can be photoionized at 10.49 eV (Figure 3b), Peak I can be associated with both isomers. However, when the photon energy was reduced to 9.65 eV, at which only **8**-*d*_3_ can be ionized, Peak I remains in TPD profiles of both *m*/*z* = 74 and 75. Moreover, the relative intensity ratio of Peak I at *m*/*z* = 74 and 75 remains essentially the same at 10.49 and 9.65 eV, indicating that the ion signals at *m*/*z* = 75 and 74 originate from the same isomer and therefore supporting the assignment of Peak I to **8**-*d*_3_.

**Figure 4:**
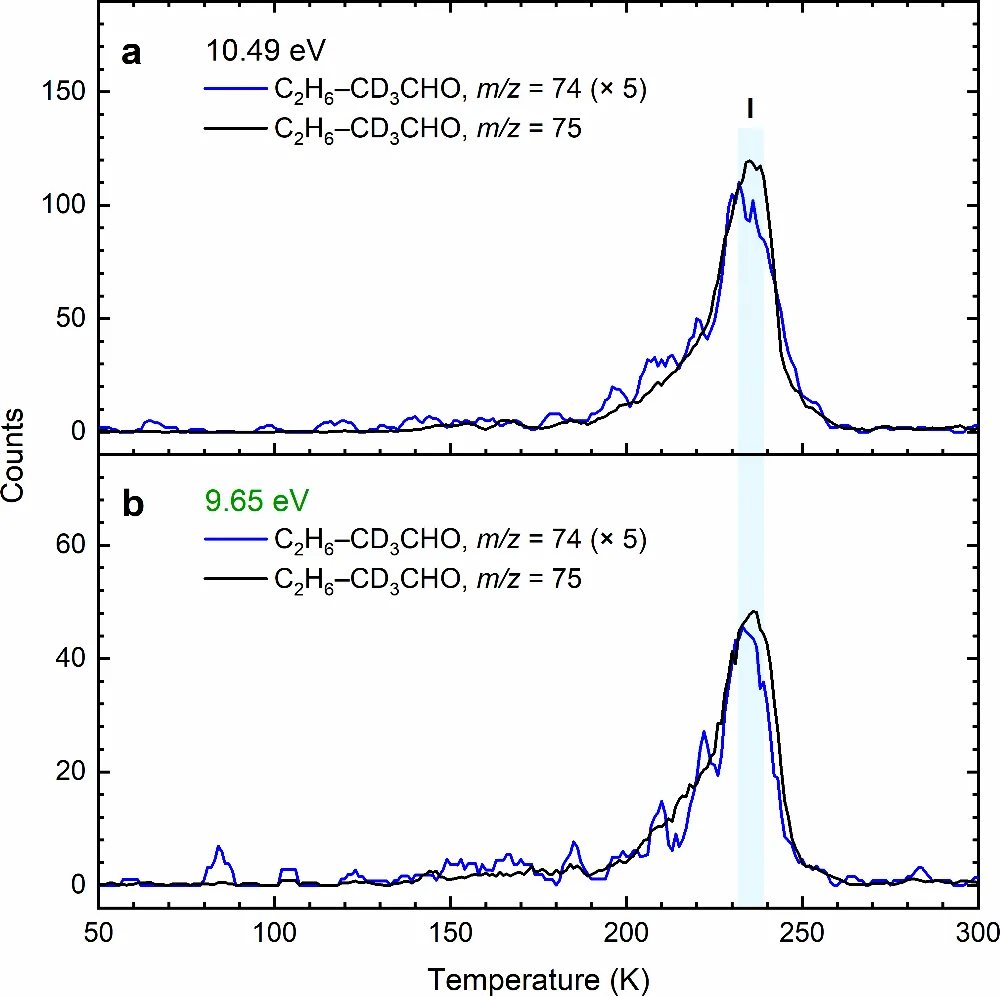
TPD profiles for *m*/*z* = 74 and 75 in irradiated C_2_H_6_–CD_3_CHO ice recorded at 10.49 eV (a) and 9.65 eV (b). The *m*/*z* = 74 traces are multiplied by a factor of 5 for clarity. The same scaling factor is applied at both photon energies, illustrating that the relative Peak I intensities at *m*/*z* = 74 and 75 remain essentially unchanged between 10.49 and 9.65 eV.

To examine whether the ion signal at *m*/*z* = 74 may arise from the dissociative photoionization of **8**-*d*_3_, gas phase experiments were conducted with isomer **8** at 10.49 eV (Figure S10); no ion signal at *m*/*z* = 71, which would correspond to a hydrogen atom loss from **8**, was detected. Therefore, ion signal at *m*/*z* = 74 observed in the C_2_H_6_–CD_3_CHO system does not arise from the fragmentation of **8**-*d*_3_. Instead, this signal is rationalized by secondary H/D scrambling involving the ĊD_2_CHO radical, formed via deuterium atom loss from CD_3_CHO, followed by rapid hydrogen atom addition to generate CD_2_HCHO prior to subsequent reactions. Consequently, no conclusive evidence for the formation of **6**-*d*_2_ can be provided; Peak II in the C_2_H_6_–CH_3_CHO experiment is associated with fragment ions produced from higher-molecular-weight products, paraldehyde (C_6_H_12_O_3_).^15^ Additionally, a high dose irradiation experiment of C_2_H_6_–CH_3_CHO ice was performed. The TPD profile at *m*/*z* = 72 reveals a broad sublimation event at 9.00 eV (Figure S11), showing that enolization occurs under high dose processing; due to overlapping ionization energies, no attempt was made to discriminate these enol isomers.

The formation yields of isomers **7** and **8** were quantified by combining calibration experiments using pure ices of **7** and **8** with the total energy deposited during electron irradiation in CO–C_3_H_8_ and C_2_H_6_–CH_3_CHO ices. The numbers of molecules in the calibration ices were determined from their thicknesses and densities. The TPD profiles of the pure reference ices used for calibration are shown in Figure S7. Comparison of the integrated PI-ReToF-MS peak area of the isomer **7** calibration ice with that of Peak I at 9.92 eV (Figure 2c) yielded (5.93 ± 1.33) × 10^15^ molecules of **7** in the irradiated CO–C_3_H_8_ ice. Similarly, comparison of the integrated peak area of the isomer 8 calibration ice with that of Peak I at 10.49 eV (Figure 3c) yielded (8.78 ± 1.76) × 10^14^ molecules of **8** in the irradiated C_2_H_6_–CH_3_CHO ice. The total energies deposited into the CO–C_3_H_8_ and C_2_H_6_–CH_3_CHO ices were calculated from the irradiation parameters and electron backscattering and transmission data obtained from CASINO simulations, yielding (1.43 ± 0.21) × 10^17^ and (1.53 ± 0.21) × 10^17^ eV, respectively. Normalization of the product numbers to the corresponding deposited energies provided formation yields of (4.14 ± 1.11) × 10^–2^ molecules eV^–1^ for **7** in the irradiated CO–C_3_H_8_ ice and (5.75 ± 1.40) × 10^–3^ molecules eV^–1^ for **8** in the irradiated C_2_H_6_–CH_3_CHO ice (Table S11). Further details of the yield calculations are provided in the Supporting Information.

## Discussion

Having provided compelling evidence for the formation of isobutyraldehyde (**7**) and its enol 2-methylprop-1-en-1-ol (**25**) in irradiated interstellar ice analogues composed of carbon monoxide–propane (CO–C_3_H_8_), as well as 2-butanone (**8**) in irradiated ethane–acetaldehyde (C_2_H_6_–CH_3_CHO) ices, we now turn our attention to potential formation mechanisms. In present experiments, the low dose irradiation was designed to minimize sequential reactions^16^ and thus to constrain the possible formation pathways of C_4_H_8_O isomers. The electrons employed here act as proxies for secondary electrons generated by GCRs as they penetrate interstellar ices in cold molecular clouds.^79^

In the exposed ices, propane (CH_3_CH_2_CH_3_, **18**) undergoes unimolecular decomposition through carbon–hydrogen bond cleavage to produce atomic hydrogen (Ḣ) together with either the isopropyl radical (CH_3_ĊHCH_3_, **20**) via reaction 1, or the *n*-propyl radical (CH_3_CH_2_ĊH_2_, **30**) via reaction 2. Reactions 1 and 2 are endoergic by 404 and 417 kJ mol^–1^,^80^ and the required energy can be supplied by the impinging GCR proxies. The suprathermal hydrogen atom can add to carbon monoxide (CO, **17**) to form the formyl radical (HĊO, **19**) through reaction 3 with a reaction exoergicity of 61 kJ mol^–1^.^16^ Previous work by Bennett et al. suggested an entrance barrier for reaction 3 to be 11 kJ mol^–1^, which can be overcome by the excess kinetic energy of the suprathermal hydrogen atom.^81^ Recall that the formation of the formyl radical (**19**) in the irradiated CO–C_3_H_8_ ice is confirmed via the infrared absorption at 1796 cm^–1^ (ν_3_).^65,70^

1 $${\mathrm{CH}}_{3}{\mathrm{CH}}_{2}{\mathrm{CH}}_{3}\;\left(18\right)\to {\mathrm{CH}}_{3}Ċ{\mathrm{HCH}}_{3}\;\left(20\right)+Ḣ,\Delta {H_{r}}^{{}^{\circ}}\left(0\;K\right)=+404\;\mathrm{kJ}\;{\mathrm{mol}}^{-1}$$

2 $${\mathrm{CH}}_{3}{\mathrm{CH}}_{2}{\mathrm{CH}}_{3}\;\left(18\right)\to {\mathrm{CH}}_{3}{\mathrm{CH}}_{2}ĊH_{2}\;\left(30\right)+Ḣ,\Delta {H_{r}}^{{}^{\circ}}\left(0\;K\right)=+417\;\mathrm{kJ}\;{\mathrm{mol}}^{-1}$$

3 $$Ḣ+\mathrm{CO}\;\left(17\right)\to HĊO\;\left(19\right),\Delta {H_{r}}^{{}^{\circ}}\left(0\;K\right)=-61\;\mathrm{kJ}\;{\mathrm{mol}}^{-1}$$

Once formed, **19** and **20** can recombine barrierlessly through carbon–carbon bond formation to yield isomer **7** via reaction 4, which is exoergic by 331 kJ mol^–1^.^80,82^ It is worth noting that the radical–radical recombination can proceed either during irradiation at 5 K or during TPD at higher temperatures. However, the FTIR spectra indicate formation of carbonyl-bearing products, consistent with but not uniquely diagnostic of C_4_ carbonyl formation at 5 K. Additionally, **7** can tautomerize to enol **25** with a reaction endoergicity of 9 kJ mol^–1^ via reaction 5.^83,84^ Although thermal tautomerization is unlikely at 5 K, energy deposited by energetic electrons as GCR proxies may promote the keto–enol tautomerization through nonequilibrium processing.^85^ Previous irradiation experiments on acetaldehyde (**3**) ices demonstrated that GCR proxies can induce keto–enol tautomerization through an intramolecular [1,3]-hydrogen shift under cold molecular cloud conditions.^62^ A similar irradiation-induced hydrogen-transfer mechanism may enable the conversion of **7** to **25** in the present ice system. Although butyraldehyde (CH_3_CH_2_CH_2_CHO, **6**) represents a plausible C_4_H_8_O isomer in the CO–C_3_H_8_ system through recombination of radical **19** and radical **30**, no evidence of **6** was observed, indicating that **30** is either not formed or is present at insufficient concentrations to produce detectable levels of **6** under our experimental conditions.

4 $$\mathrm{HĊO}\;\left(19\right)+{\mathrm{CH}}_{3}Ċ{\mathrm{HCH}}_{3}\;\left(20\right)\to {\left({\mathrm{CH}}_{3}\right)}_{2}\mathrm{CHCHO}\;\left(7\right),\Delta {H_{r}}^{{}^{\circ}}\left(0\;K\right)=-331\;\mathrm{kJ}\;{\mathrm{mol}}^{-1}$$

5 $${\left({\mathrm{CH}}_{3}\right)}_{2}\mathrm{CHCHO}\;\left(7\right)⇋{\left({\mathrm{CH}}_{3}\right)}_{2}\mathrm{CCHOH}\;\left(25\right),\Delta {H_{r}}^{{}^{\circ}}\left(298\;K\right)=+9\;\mathrm{kJ}\;{\mathrm{mol}}^{-1}$$

In the C_2_H_6_–CH_3_CHO system, upon interaction with energetic electrons as GCR proxies, acetaldehyde (CH_3_CHO, **3**) can undergo carbon–hydrogen bond cleavage to form the hydrogen atom plus the acetyl radical (CH_3_ĊO, **22**) or the vinoxy radical (ĊH_2_CHO, **26**) through reactions 6 and 7, which are endoergic by 367 and 394 kJ mol^–1^, respectively.^80^ Under the low dose conditions, acetaldehyde (**3**) is expected to produce **22** more efficiently than **26**.^73,86,87^ Recall that acetyl radical (**22**) was identified by FTIR absorptions at 1839 cm^–1^ for **22** (CH_3_ĊO, ν_3_) in irradiated C_2_H_6_–CH_3_CHO, and at 1849 cm^–1^ for **22-** *d*_3_ (CD_3_ĊO, ν_3_) in the irradiated C_2_H_6_–CD_3_CHO ice. Ethane (CH_3_CH_3_, **21**) undergoes carbon–hydrogen bond cleavage, yielding the ethyl radical (CH_3_ĊH_2_, **23**) via reaction 8, which is endoergic by 416 kJ mol^–1^.^80^ If radicals **22** and **23** are in a favorable, neighboring geometry within the ice matrix, barrierless carbon–carbon coupling can form isomer **8** through reaction 9, which is exoergic by 345 kJ mol^–1^.^80^

6 $${\mathrm{CH}}_{3}\mathrm{CHO}\;\left(3\right)\to {\mathrm{CH}}_{3}ĊO\;\left(22\right)+Ḣ,\Delta {H_{r}}^{{}^{\circ}}\left(0\;K\right)=+367\;\mathrm{kJ}\;{\mathrm{mol}}^{-1}$$

7 $${\mathrm{CH}}_{3}\mathrm{CHO}\;\left(3\right)\to ĊH_{2}\mathrm{CHO}\;\left(26\right)+Ḣ,\Delta {H_{r}}^{{}^{\circ}}\left(0\;K\right)=+394\;\mathrm{kJ}\;{\mathrm{mol}}^{-1}$$

8 $${\mathrm{CH}}_{3}{\mathrm{CH}}_{3}\;\left(21\right)\to {\mathrm{CH}}_{3}ĊH_{2}\;\left(23\right)+Ḣ,\Delta {H_{r}}^{{}^{\circ}}\left(0\;K\right)=+416\;\mathrm{kJ}\;{\mathrm{mol}}^{-1}$$

9 $${\mathrm{CH}}_{3}ĊO\;\left(22\right)+{\mathrm{CH}}_{3}ĊH_{2}\;\left(23\right)\to {\mathrm{CH}}_{3}{\mathrm{CH}}_{2}{\mathrm{COCH}}_{3}\;\left(8\right),{{\Delta H}_{r}}^{{}^{\circ}}\left(0\;K\right)=-345\;\mathrm{kJ}\;{\mathrm{mol}}^{-1}$$

Carbon monoxide (**17**) and acetaldehyde (**3**) have been detected in interstellar ices at up to 55% ^14,45,46^ and 10%^53^ relative to water. Although the abundances of ethane (**21**) and propane (**18**) remain poorly constrained observationally, they can readily form in methane-bearing interstellar analogue ices under ionizing radiation such as GCRs.^47,48^ Therefore, the precursor combinations investigated here are chemically plausible constituents in interstellar icy grains. However, these binary ice mixtures were designed as simplified mechanistic systems to isolate the relevant reaction pathways and do not represent typical interstellar ice compositions, in which water is a major component.^88^ In water-rich interstellar ices, the ice composition may affect radical formation and subsequent reactions. Further experiments with water-containing ices may reveal the formation of other compounds and additional formation pathways. During the evolution of a cold molecular cloud toward a star-forming region, warming of icy grain mantles can release newly formed organics into the gas phase.^16^ The detection of **7** and **8** during TPD thus simulates the thermal desorption of these carbonyls during the warm-up stage. Since aldehydes and ketones constitute a key class of interstellar and meteoritic complex organic molecules,^24–27^ the present work provides laboratory evidence that C_4_ carbonyls can be formed from simple ice constituents through irradiation-driven radical chemistry. Beyond astrochemical molecular growth, the formation of **7** and **8** provides possible connections to broader prebiotic reaction networks. As potential downstream chemistry, **7** and **8** may participate in Strecker-type chemistry to yield valine (**13**) and isovaline (**14**), respectively. In addition, oxidation of **7** could yield isobutyric acid (**15**), a precursor to branched-chain fatty acid chemistry, while carboxylation of **8** could provide access to levulinic acid (**16**). These downstream reactions were not investigated in the present study but are presented as plausible chemical connections. Therefore, **7** and **8** play crucial roles in linking low-temperature carbonyl formation to amino acids and fatty acids. The detection of **7**, **8**, and **13**–**16** in carbonaceous meteorites and asteroid-return samples^27,34,39,42–44,89^ is consistent with a broader scenario in which complex organic molecules formed in extraterrestrial environments,^9^ while the specific formation pathways in their parent bodies remain to be established. Altogether, the present study establishes a mechanistic foundation for the abiotic synthesis of C_4_ carbonyls in interstellar ices, advancing our understanding of how biologically relevant molecules can be synthesized via nonequilibrium chemistry in deep space.

## Conclusions

To conclude, we present the first formation pathways of isobutyraldehyde (**7**) and its enol 2-methylprop-1-en-1-ol (**25**), as well as 2-butanone (**8**), in low-temperature interstellar ice analogues composed of carbon monoxide–propane (CO–C_3_H_8_) and ethane–acetaldehyde (C_2_H_6_–CH_3_CHO). The ice mixtures were exposed to energetic electrons, which simulate secondary electrons produced by GCRs as they penetrate icy grains in cold molecular clouds, corresponding to (8 ± 2) × 10^5^ yr of GCR exposure.^74^ Isomers **7**, **8**, and **25** were identified in the gas phase during TPD utilizing photoionization reflectron time-of-flight mass spectrometry (PI-ReToF-MS) along with isotopic substitution experiments. Isomers **7** and **8** were formed via radical–radical recombination of the formyl (HĊO, **19**) radical with the isopropyl (CH_3_ĊHCH_3_, **20**) radical, and the acetyl (CH_3_ĊO, **22**) radical with the ethyl (CH_3_ĊH_2_, **23**) radical, respectively; the enol **25** was produced through keto–enol tautomerization of **7**. The detection of these C_4_ carbonyls highlights the critical roles of nonequilibrium chemistry in providing viable routes to complex aldehydes, ketones, and their enols under astrophysical conditions. It is important to note that CO–C_3_H_8_ and C_2_H_6_–CH_3_CHO ices employed in this study serve as simplified model systems to probe the formation mechanisms of C_4_H_8_O isomers upon exposure to GCR proxies. Further studies using realistic multicomponent interstellar ice compositions may reveal additional reaction pathways and products.

## Materials and Methods

The experiments involved the preparation of low-temperature carbon monoxide–propane (CO–C_3_H_8_), ethane–acetaldehyde (C_2_H_6_–CH_3_CHO), and isotopically labeled ethane–acetaldehyde-*d*_3_ (C_2_H_6_–CD_3_CHO) ice mixtures on silver substrate at 5 K in an ultrahigh vacuum chamber maintained at pressure of 5 × 10^–11^ Torr. The carbon monoxide–propane and ethane–acetaldehyde ices had compositions of 1:(1.1 ± 0.2) and (1.5 ± 0.3):1, respectively, as determined from infrared band integrations. The ices were irradiated with 5 keV electrons over an area of 1.6 cm^2^ to initiate nonequilibrium chemistry. Low dose experiments were conducted at 20 nA for 5 min, corresponding to an electron flux of 7.8 × 10^10^ electrons cm^–2^ s^–1^ and a fluence of 2.3 × 10^13^ electrons cm^–2^. The high dose C_2_H_6_–CH_3_CHO experiment was performed at 106 nA for 60 min, corresponding to an electron flux of 4.1 × 10^11^ electrons cm^–2^ s^–1^ and a fluence of 1.5 × 10^15^ electrons cm^–2^. CASINO 2.42 simulations showed that 99% of the deposited electron energy in low dose irradiation was confined within depths of 659 ± 22 nm for the CO–C_3_H_8_ ice and 532 ± 17 nm for the C_2_H_6_–CH_3_CHO ice. Infrared spectra were recorded in situ before, during, and after irradiation. During TPD, the CO–C_3_H_8_ ices were heated from 5 to 320 at 1 K min^–1^, whereas the C_2_H_6_–CH_3_CHO and C_2_H_6_–CD_3_CHO ices were heated at 0.5 K min^–1^. The resulting ions during TPD were mass analyzed by tunable vacuum ultraviolet photoionization reflectron time-of-flight mass spectrometry (PI-ReTOF-MS). Adiabatic ionization energies and relative energetics were calculated using the CBS-QB3 composite method. Additional experimental and computational details are provided in the Supporting Information.

## Supplementary Material



## Notes

Methods (Experimental and Computational); Yield calculation; Aldehydes and ketones identified in interstellar medium (Figure S1); FTIR spectra data of irradiated carbon monoxide–propane and ethane–acetaldehyde ices (Figures S2–S5, Tables S1–S4); PI-ReToF-MS data during TPD of carbon monoxide–propane and ethane–acetaldehyde ices (Figures S6 and S8); TPD profiles of *m*/*z* = 72 for pure 2-butanone, butyraldehyde and isobutyraldehyde ices (Figure S7); Proposed formation pathways of isomers **6** and **8** in irradiated C_2_H_6_–CD_3_CHO ices (Figure S9); Gas phase mass spectra of 2-butanone and butyraldehyde samples recorded at 10.49 eV (Figure S10); TPD profiles of *m*/*z* = 72 recorded at 9.00 eV in irradiated C_2_H_6_–CH_3_CHO ices (Figure S11); Error analysis of adiabatic ionization energies and relative energies (Tables S5–S10); Parameters used to calculate the yields of isobutyraldehyde and 2-butanone (Table S11); Experimental conditions (Tables S12 and S13); VUV generation parameters (Table S14); Cartesian coordinates (Å), and harmonic frequencies (cm^–1^) of isomer **6**, **8**, **25**, **27**, **28**, **29** (Table S15–S20) (PDF)
