# Supplementary material for: Nonequilibrium Formation of C4 Carbonyls in Interstellar Ices Linked to Amino Acids
Source: ACS Cent Sci. 2026 Sep 2;12(9):1458–67. doi: 10.1021/acscentsci.6c00923 (PMC13614037; doi:10.1021/acscentsci.6c00923)
Supplement: Supplementary file 1 [file oc6c00923_si_001.pdf]

Supporting Information

Nonequilibrium Formation of C_4 Carbonyls in Interstellar Ices Linked to Amino Acids

Zesen Wang[1,2], Jia Wang[1,2], Anastasia S. Shishova[3], Sergey O. Tuchin[3], Mason McAnally[1,2], Andrew M. Turner[1,2], Niki Jabari[1,2], and Ralf I. Kaiser[1,2]*

[1] *W. M. Keck Research Laboratory in Astrochemistry, University of Hawaii at Manoa, Honolulu, Hawaii 96822, United States*

[2] *Department of Chemistry, University of Hawaii at Manoa, Honolulu, Hawaii 96822, United States*

[3] *Samara National Research University, Samara 443086, Russia*

[*]Corresponding Author:

Ralf I. Kaiser, ralfk@hawaii.edu

This PDF file includes:

Methods (Experimental and Computational)

Yield calculation

Figures S1 to S11

Tables S1 to S20

Supplementary References

Methods

Experimental. The experiments were carried out in a stainless-steel ultrahigh vacuum chamber at the W. M. Keck Research Laboratory in Astrochemistry, University of Hawai‘i at Mānoa.[1–4] The chamber was maintained at a base pressure of 5×10^{-11} Torr by magnetically levitated turbomolecular pumps (Osaka, TG1300MUCWB, TG420MCAB) backed by a hydrocarbon-free dry scroll pump (XDS35i, BOC Edwards). A polished silver substrate (0.6 inch × 0.5 inch) was interfaced to a closed-cycle helium refrigerator (Sumitomo Heavy Industries, RDK-415E) and cooled to 5 K. The cold head could be rotated freely and translated vertically through a doubly differentially pumped rotational feedthrough (Thermionics Vacuum Products, RNN-600/FA/MCO) and an adjustable bellows (McAllister, BLT86) to allow ice deposition, electron irradiation, infrared spectroscopic characterization, and temperature-programmed desorption measurements. Carbon monoxide (CO; Sigma-Aldrich, >99%), propane (C_3H_8; The Specialty Gas Group, Grade 5.0), ethane (C_2H_6; Linde, Grade 5.0), acetaldehyde (CH_3CHO; Sigma-Aldrich, >99.5%), acetaldehyde-d_3 (CD_3CHO; Sigma-Aldrich, >98% atom D), isobutyraldehyde ($(CH_3)_2CHCHO$; Sigma-Aldrich, >99.0%), 2-butanone ($CH_3CH_2COCH_3$; Sigma-Aldrich, >99.0%), and butyraldehyde ($CH_3CH_2CH_2CHO$; Sigma-Aldrich, >99.0%) were used. Liquid acetaldehyde and acetaldehyde-d_3 samples were stored in borosilicate vials and purified by several freeze–pump–thaw cycles to remove residual atmospheric gases.

CO–C_3H_8 was prepared by premixing CO and C_3H_8 in a gas-mixing chamber with a 5:3 pressure ratio of carbon monoxide to propane. The premixed gas was leaked into the main chamber via a glass capillary array at a deposition pressure of 5×10^{-8} Torr for 8 min onto the silver substrate held at 5 K. The resulting CO–C_3H_8 ice thickness was 717 ± 24 nm, as determined by laser interferometry[5] via a photodiode and a helium-neon laser (632.8 nm). The average refractive index of 1.326 ± 0.004 was used to derive the thickness of the mixture ice (CO–C_3H_8) from the refractive indexes of carbon monoxide ice (n = 1.292 ± 0.003)[6] and that of propane ice (n = 1.357 ± 0.005).[7] A Fourier transform infrared (FTIR) spectrometer (Bruker, Vertex Neo R) measured the ice before, during, and after electron irradiation in the range of 6000−500 cm^{-1} with a resolution of 4 cm^{-1}. The ratio of carbon monoxide to propane in the ice mixture was determined to be 1: (1.1 ± 0.2) (Table S12). This was done by integrating the infrared absorptions of carbon monoxide at

2137 cm^{-1} (ν_1, 1.1 × 10^{-17} cm molecule^{-1})[8] and of propane at 2957 cm^{-1} (ν_1, 4.46 × 10^{-17} cm molecule^{-1}).[7]

C_2H_6–CH_3CHO ices were prepared by premixing C_2H_6 and CH_3CHO in a gas-mixing chamber with a 2.5: 1 pressure ratio of ethane to acetaldehyde. The gas mixture was deposited onto the 5 K substrate at 3 × 10^{-8} Torr for 7 min. Based on the average refractive index of 1.325 ± 0.009, derived from the refractive indexes of ethane ice (n = 1.340 ± 0.015)[9] and acetaldehyde ice (n = 1.303 ± 0.005),[10] the ice thickness of the C_2H_6–CH_3CHO mixture ice was determined to be 719 ± 24 nm. The ratio of ethane to acetaldehyde in the ice mixture was determined as (1.5 ± 0.3): 1 based on the integrated infrared absorptions of ethane at 817.1 cm^{-1} (ν_{12}, 1.99 × 10^{-18} cm molecule^{-1}),[9] and of acetaldehyde at 1721 cm^{-1} (ν_4, 2.98 × 10^{-17} cm molecule^{-1}).[11] The isotopically labeled C_2H_6–CD_3CHO ices were prepared analogously by replacing CH_3CHO with CD_3CHO. The C_2H_6–CD_3CHO ice composition and thickness were determined to be (1.5 ± 0.3): 1 and 719 ± 24 nm, respectively (Table S13).

After deposition, the ice mixtures were irradiated with 5 keV electrons generated by an electron gun (SPECS, EQ PU-22); the electron beam was scanned over an area of 1.6 cm^2. Low dose (20 nA, 5 min) irradiations were carried out, whereas a high dose (106 nA, 1 h) irradiation was conducted for the C_2H_6–CH_3CHO ice (Table S12 and Table S13). Monte Carlo simulations performed with CASINO 2.42 were used to calculate the electron penetration depths and irradiation doses. The low dose irradiation of CO–C_3H_8 ice corresponds to doses of 0.18 ± 0.03 eV molecule^{-1} for CO and 0.29 ± 0.05 eV molecule^{-1} for C_3H_8 (Table S12), whereas the low dose irradiation of C_2H_6–CH_3CHO ice corresponds to doses of 0.20 ± 0.03 eV molecule^{-1} for C_2H_6 and 0.30 ± 0.05 eV molecule^{-1} for CH_3CHO (Table S13). These low doses correspond to (8 ± 2) × 10^5 yr of GCR exposure in a cold molecular cloud.[12] The average penetration depths were 313 ± 31 nm for CO–C_3H_8 ice and 338 ± 34 nm for C_2H_6–CH_3CHO ice. The depths containing 99% of the deposited electron energy were 659 ± 22 nm for CO–C_3H_8 ices, and 532 ± 17 nm for C_2H_6–CH_3CHO ices; both depths are smaller than the total ice thicknesses, preventing electron-induced interactions with the silver substrate. The average densities for CO–C_3H_8 ices of 0.754 ± 0.014 g cm^{-3} derived from 1.029 ± 0.005 g cm^{-3} for CO ice[6] and 0.653 ± 0.005 g cm^{-3} for C_3H_8 ice[7], and for C_2H_6–CH_3CHO ices of 0.751 ± 0.039 g cm^{-3} derived from 0.719 ± 0.07 g cm^{-3} for C_2H_6 ice[13] and 0.787 ± 0.005 g cm^{-3} for CH_3CHO ice[10] were used in these calculations. After irradiation, the

ices were subjected to temperature-programmed desorption. The $CO–C_3H_8$ ices were heated from 5 to 320 K at 1 K min^{-1}, whereas the $C_2H_6–CH_3CHO$ and $C_2H_6–CD_3CHO$ ices were heated from 5 to 320 K at 0.5 K min^{-1}. During TPD, subliming molecules were photoionized in the gas phase by pulsed VUV light generated at 30 Hz via two photon resonant four-wave mixing in a krypton/xenon gas jet. The fundamental laser beams were generated via two tunable dye lasers (Sirah Lasertechnik, Cobra-Stretch) and two Nd:YAG lasers (SpectraPhysics, Quanta Ray PRO 270–30). Detailed VUV photon generation parameters are provided in Table S14. The generated VUV light was spatially separated from residual laser beams by a biconvex lithium fluoride lens in an off-axis geometry and directed 2.0 ± 0.5 mm above the silver substrate to photoionize subliming molecules during TPD. The VUV flux was monitored by a Faraday cup during TPD. At each acquisition time, the measured ion signal was divided by the corresponding VUV signal and multiplied by a common scaling constant to obtain the VUV-flux-corrected ion intensity. The same scaling constant was applied to all datasets. Peak fitting and integration were performed using the VUV-flux-corrected profiles. Uncertainty associated with the flux correction may contribute to the uncertainties of the integrated peak areas and area ratios but does not affect the overall product assignments, which are based on the combined photon-energy-dependent ion signals, isotopic labeling experiments, and calculated ionization energies. The resulting ions from VUV photoionization were mass analyzed using a reflectron time-of-flight mass spectrometer and detected with a dual microchannel plate detector (Jordan TOF Products). The ion signals were amplified with a preamplifier (Ortec 9305), discriminated, and recorded with a multichannel scaler (FAST ComTec, MCS6A). Each mass spectrum represented the accumulation of 3600 laser shots over 2 min with a temporal resolution of 3.2 ns. TPD profiles were corrected for variations in the VUV photon flux throughout each experiment. No unexpected or unusually high safety hazards were encountered in the experiments.

Computational. All ab initio electronic structure calculations were performed using the GAUSSIAN 09 software package.[14] The computational study of the conformational diversity of the products was carried out in several stages. At the first stage, the starting geometries of each of the six backbone isomer of C_4H_8O were optimized at the B3LYP/6-31G* level: butyraldehyde (**6**), 2-butanone (**8**), 2-methylprop-1-en-1-ol (**25**), 1-buten-1-ol (**27**), 1-buten-2-ol (**28**) and 2-buten-2-ol (**29**). The resulting structures were used as templates for generating a complete set of rotamers by systematically rotating around all conformationally active σ-bonds in 120° increments. All

geometries generated in this way (3^n, where n is the number of active bonds) were reoptimized at the same level of theory B3LYP/6-31G*. A custom automated filtering algorithm based on the analysis of interatomic distances in the third coordination sphere was used to identify unique structures. For each optimized structure, an ascending vector of the indicated distances was formed, acting as a structural "fingerprint" that remains invariant under atom renumbering, molecular translation, overall rotation, and mirror reflection. The criterion for structural identity or the formation of enantiomeric pairs was the proximity to zero of the Euclidean norm of the difference between the corresponding vectors. The analysis of the distribution of the obtained values of the Euclidean norms revealed a bimodal character, which made it possible to objectively establish a threshold value for the automatic separation of pairs of conformers into identical and unique ones. The final stage of the calculations consisted in determining high-precision values of relative enthalpies and adiabatic ionization energies for all selected unique structures. Calculations were carried out using the CBS-QB3 composite method to obtain accurate energies for both the neutral and cationic states of each species. This approach yields molecular parameters with high precision: bond lengths within 0.01–0.02 Å, bond angles within 1–2°, and relative energies within 4–8 $kJ\,mol^{-1}$.[15] This method includes geometry optimization and frequency calculation at the B3LYP/6-311G(2d,d,p) level, followed by a series of single-point energy calculations at levels up to CCSD(T)/6-31+G(d) and extrapolation to the limit of the complete basis set using empirical corrections. The optimized Cartesian coordinates and harmonic frequencies of the six C_4H_8O isomers are compiled in Tables S15–S20.

Yield calculation

The product yields reported in Table S11 were determined by combining blank calibration experiments with the total energy deposited into the ice during electron irradiation. Blank calibration ices of pure isobutyraldehyde (**7**) and pure 2-butanone (**8**) were prepared by depositing commercially obtained reference compounds (Sigma-Aldrich, purity ≥ 99.0%) onto the silver wafer. The deposited area was 1.94 cm^2, corresponding to the area of the silver wafer. The total number of molecules in each calibration ice was determined from $N = \rho d A N_A/M$, where ρ, d, A, N_A, and M are the density, measured ice thickness, deposition area, Avogadro constant, and molar mass, respectively. Using d = 230 ± 23 nm (Table S12) and ρ = 0.79 g cm^{-3},[16] the pure isomer **7** calibration ice contained $(2.94 \pm 0.66)\times10^{17}$ molecules. Using d = 120 ± 24 nm (Table S13) and ρ = 0.721 g cm^{-3},[17] the pure isomer **8** calibration ice contained $(1.40 \pm 0.28) \times 10^{17}$ molecules.

Integration of the corrected blank TPD peaks gave areas of 118661 for **7** and 372009 for **8**. These values provided the calibration factors relating the integrated PI-ReTOF-MS signal to the number of molecules under identical experimental conditions. The same factors were subsequently applied to the assigned product peaks in the irradiated mixed ices recorded at the same photoionization energies, 9.92 eV for isomer **7** in the CO–C_3H_8 ice and 10.49 eV for isomer **8** in the C_2H_6–CH_3CHO ice. In this way, the total numbers of product molecules formed in the irradiation experiments were determined to be $(5.93 \pm 1.33) \times 10^{15}$ for **7** and $(8.78 \pm 1.76) \times 10^{14}$ for **8**. Because the blank calibration and irradiation experiments were performed for the same molecule at the same photoionization energy under identical PI-ReTOF-MS conditions, the photoionization response is inherently included in the calibration factor and no separate correction for the photoionization cross section was required in this step.

The total deposited energy was calculated from the number of incident electrons and the effective energy deposited into the ice after accounting for backscattered and transmitted electrons. The total number of incident electrons, N_e, was obtained from the irradiation current and irradiation time according to $N_e = It/e$, where I is the current, t is the irradiation time, and e is the elementary charge. The total deposited energy, E_{dose}, was then calculated as $E_{dose} = N_e (E_{init} - f_{bs}E_{bs} - f_{trans}E_{trans})$, where E_{init} is the initial electron energy (5000 eV), and E_{bs}, E_{trans}, f_{bs}, f_{trans} are the average kinetic energies and fractions of backscattered and transmitted electrons obtained from CASINO

simulations. Using the parameters listed in Table S11, the total deposited energies were calculated to be $(1.43 \pm 0.21) \times 10^{17}$ eV for the CO–C_3H_8 ice and $(1.53 \pm 0.21) \times 10^{17}$ eV for the C_2H_6–CH_3CHO ice. Finally, the product yield in molecules eV^{-1} was obtained by dividing the total number of product molecules by the total deposited energy, $Y = N_{product} / E_{dose}$. Accordingly, the formation yields were determined to be $(4.14 \pm 1.11) \times 10^{-2}$ molecules eV^{-1} for **7** and $(5.75 \pm 1.40) \times 10^{-3}$ molecules eV^{-1} for **8** (Table S11).

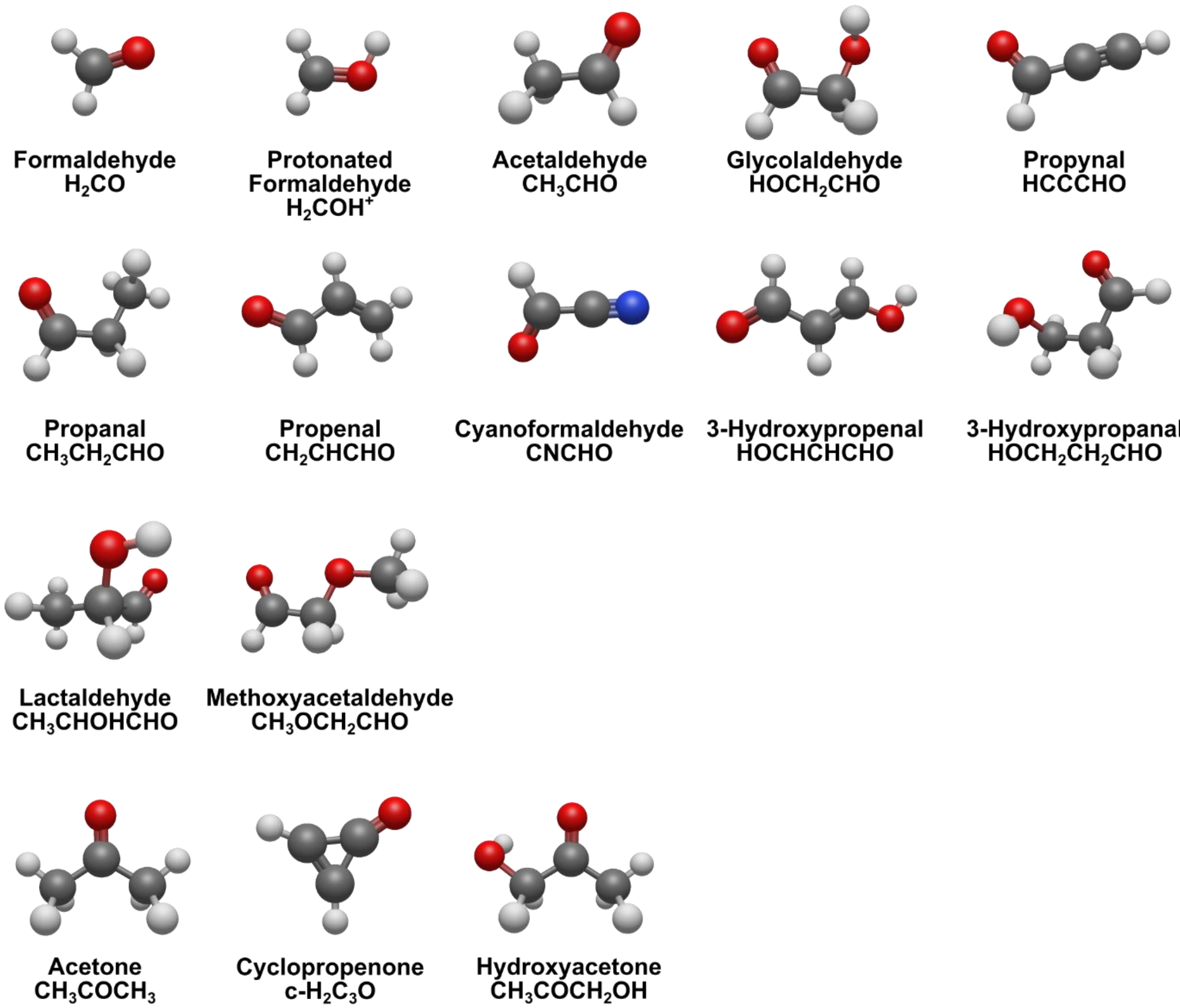

Figure S1. Aldehydes and ketones identified in interstellar medium. The atoms are color-coded in white (hydrogen), gray (carbon), red (oxygen), and blue (nitrogen).

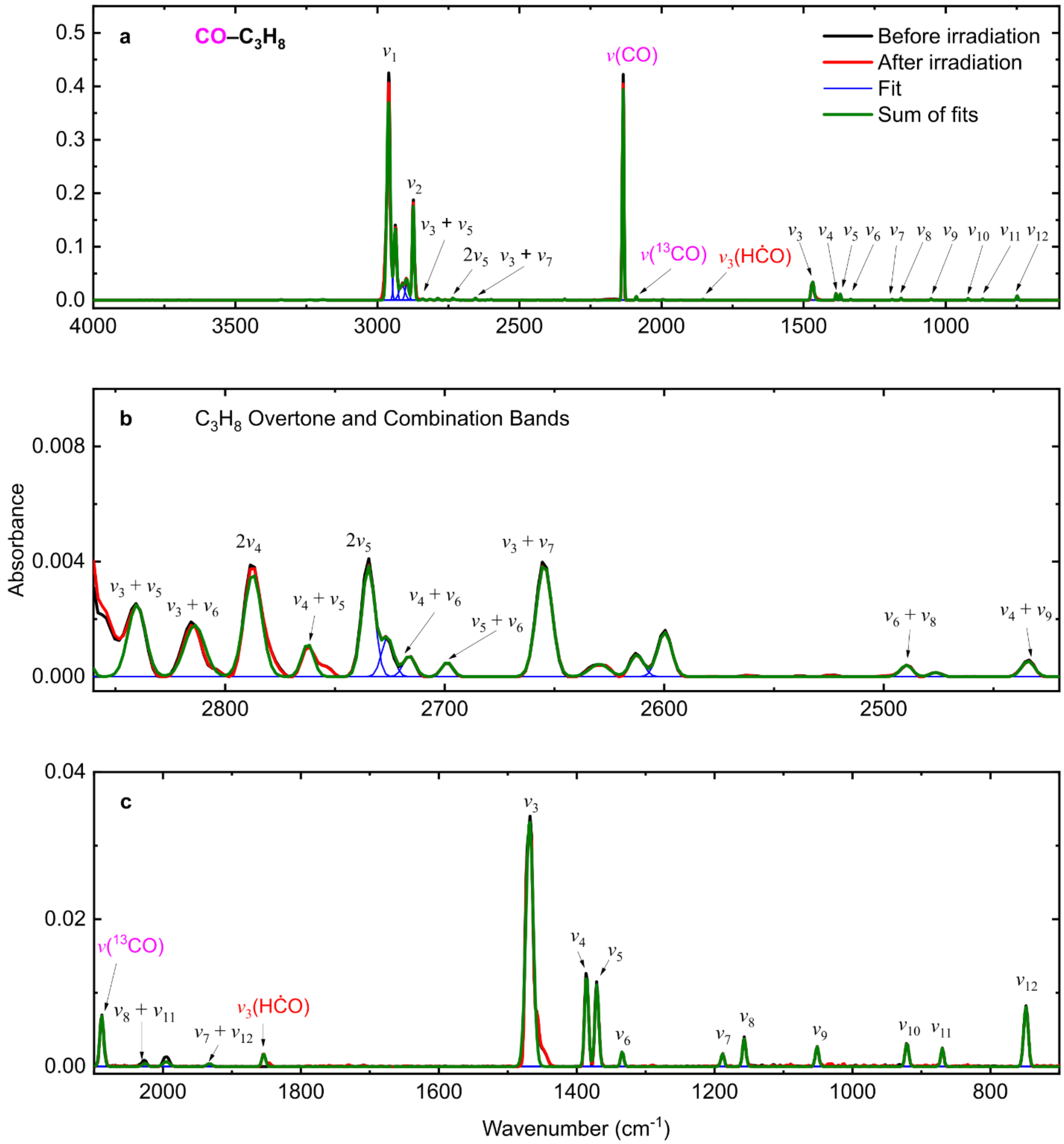

Figure S2. Infrared spectra of CO−C_3H_8 ice before (black) and after (red) irradiation (20 nA, 5 minutes) at 5 K. Magnified views and Gaussian deconvolutions of selected regions 2860–2420 cm^{-1}, and 2100–700 cm^{-1} are shown in (b), and (c) respectively. The assignments of the absorptions of CO, C_3H_8, and new absorptions after irradiation are labeled in magenta, black, and red, respectively. Detailed assignments are compiled in Table S1.

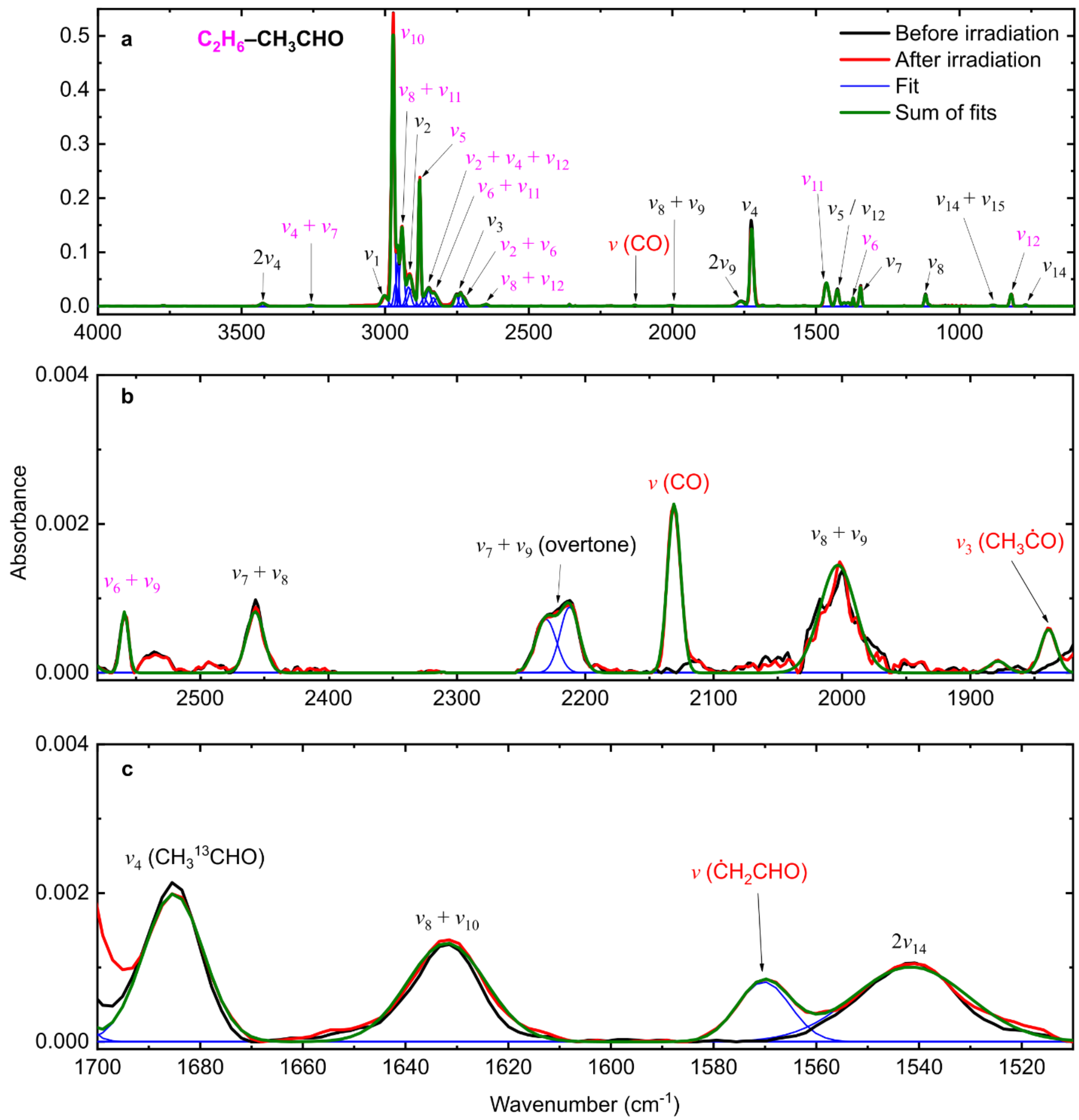

Figure S3. Infrared spectra of (a) C_2H_6−CH_3CHO ice before (black) and after (red) low dose irradiation (20 nA, 5 minutes) at 5 K. Magnified views and Gaussian deconvolutions of selected regions 2580–1820 cm^{-1} and 1700–1510 cm^{-1} are shown in (b) and (c) respectively. The assignments of the absorptions of C_2H_6, CH_3CHO, and new absorptions after irradiation are labeled in magenta, black, and red, respectively. Detailed assignments are compiled in Table S2.

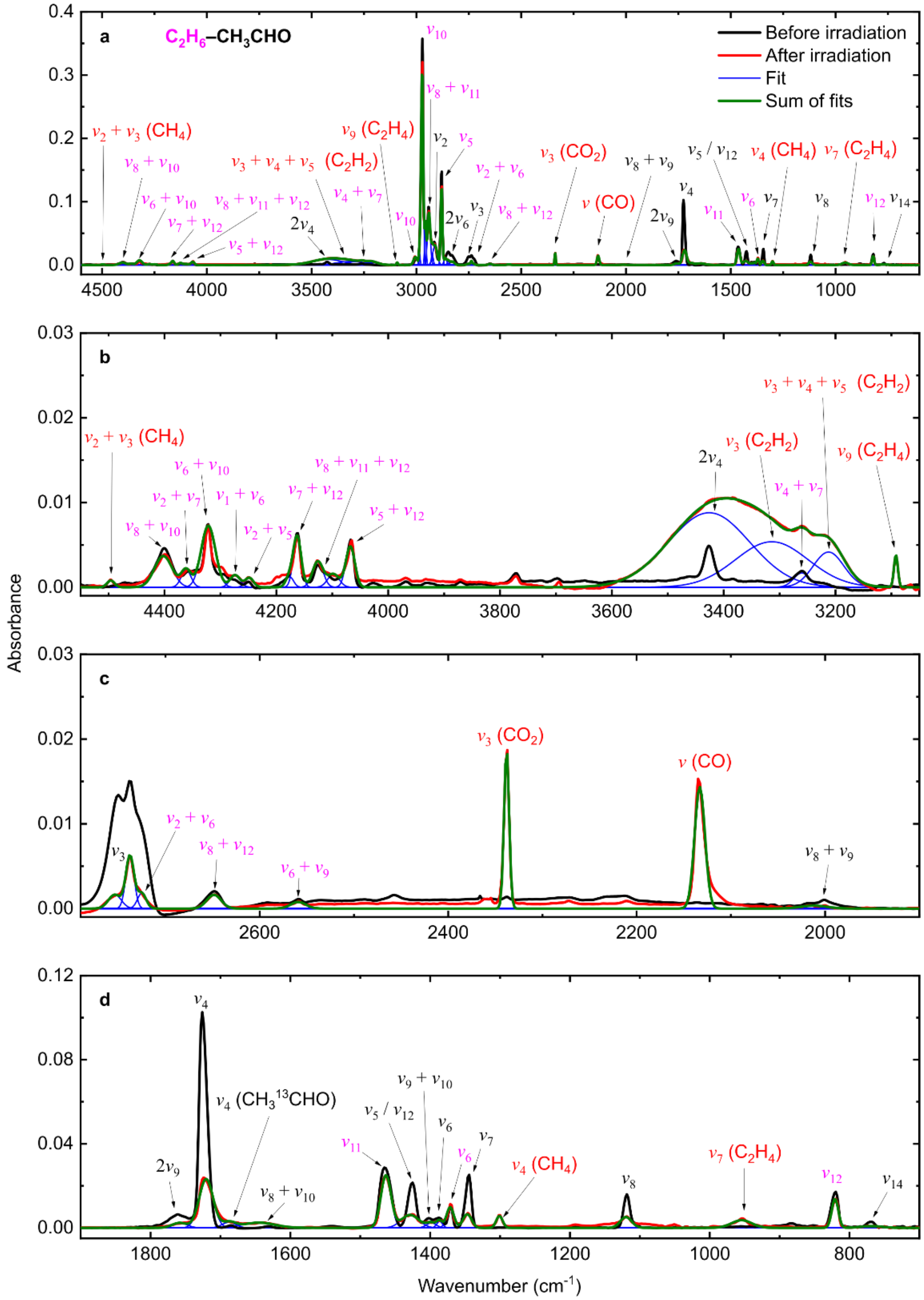

Figure S4. Infrared spectra of (a) C_2H_6−CH_3CHO ice before (black) and after (red) high dose irradiation (106 nA, 60 minutes) at 5 K. Magnified views and Gaussian deconvolutions of selected regions 4550–3050 cm^{-1}, 2790–1900 cm^{-1} , and 1900–700 cm^{-1} are shown in (b), (c), and (d) respectively. The assignments of the absorptions of C_2H_6, CH_3CHO, and new absorptions after irradiation are labeled in magenta, black, and red, respectively. Detailed assignments are compiled in Table S3.

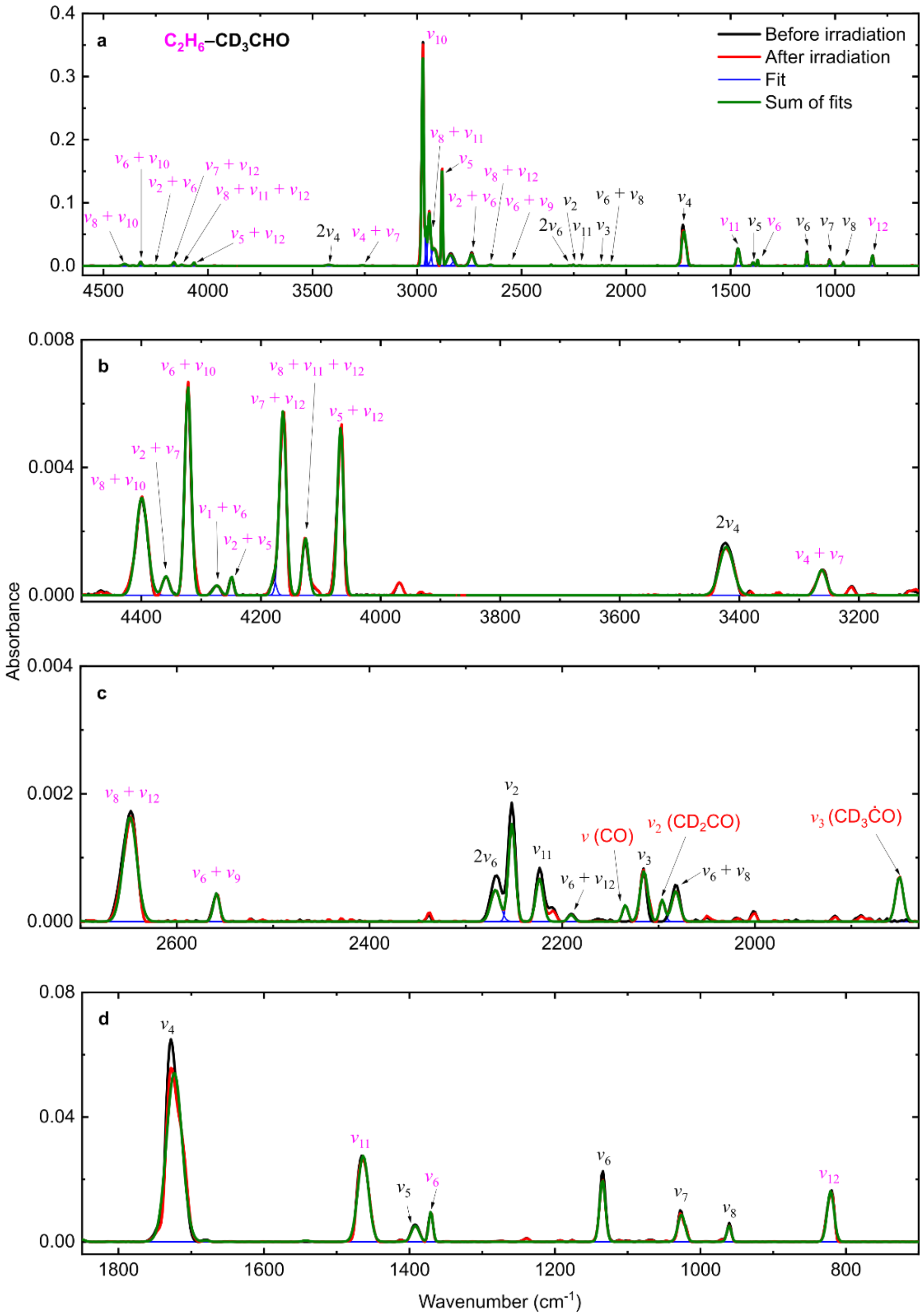

Figure S5. Infrared spectra of (a) C_2H_6–CD_3CHO ice before (black) and after (red) low dose irradiation (23 nA, 5 minutes) at 5 K. Magnified views and Gaussian deconvolutions of selected regions 4500–3100 cm^{-1}, 2700–1830 cm^{-1} , and 1850–700 cm^{-1} are shown in (b), (c) and (d) respectively. The assignments of the absorptions of C_2H_6, CD_3CHO, and new absorptions after irradiation are labeled in magenta, black, and red, respectively. Detailed assignments are compiled in Table S4.

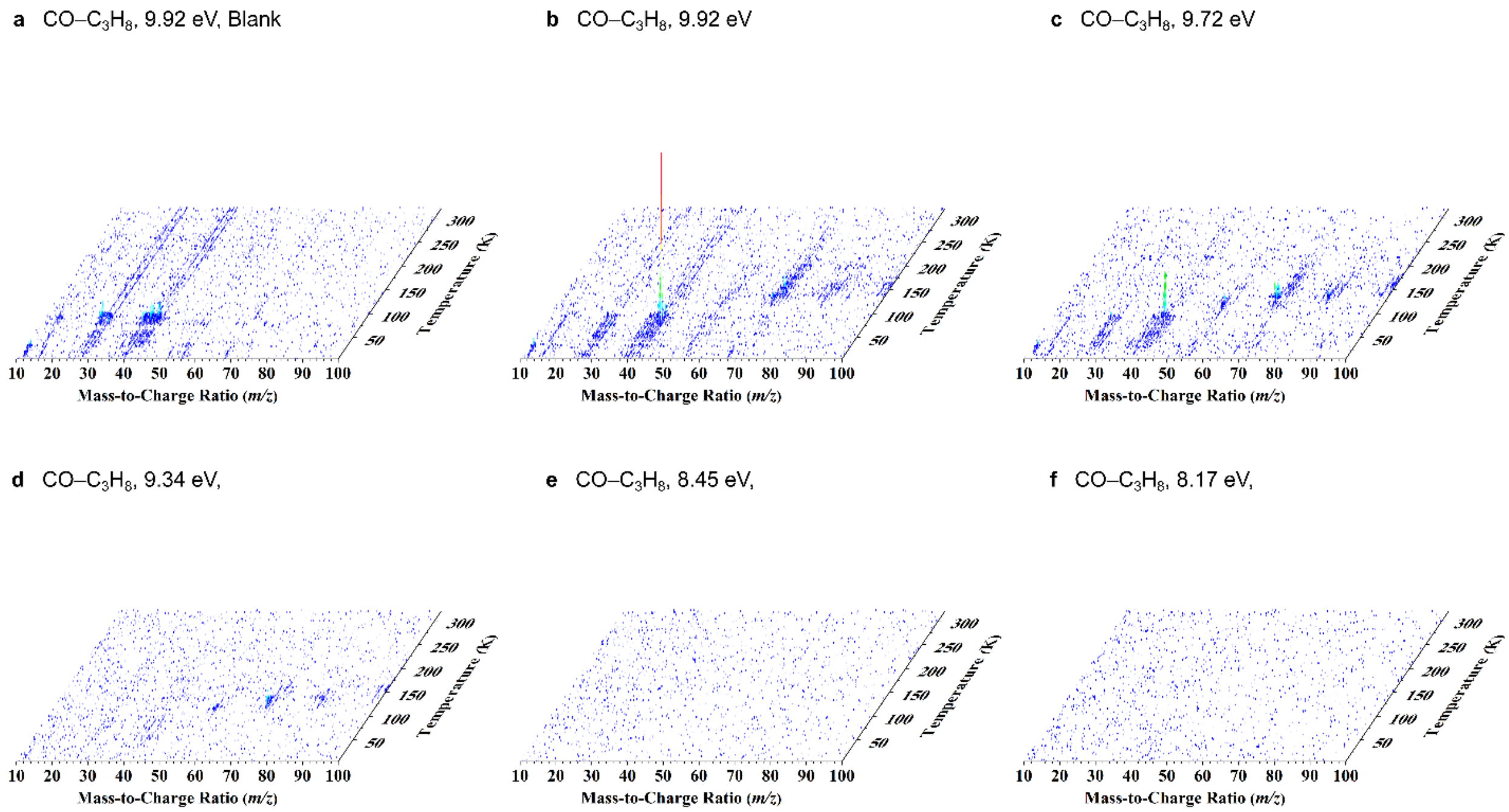

Figure S6. PI-ReToF-MS data during TPD of carbon monoxide−propane ices with low dose (20 nA, 5 minutes) irradiation. Data were recorded for the unirradiated (blank) CO–C_3H_8 ice at 9.92 eV (a), and the irradiated CO–C_3H_8 ice at 9.92 eV (b), 9.72 eV (c), 9.34 eV (d), 8.45 eV (e), and 8.17 eV (f).

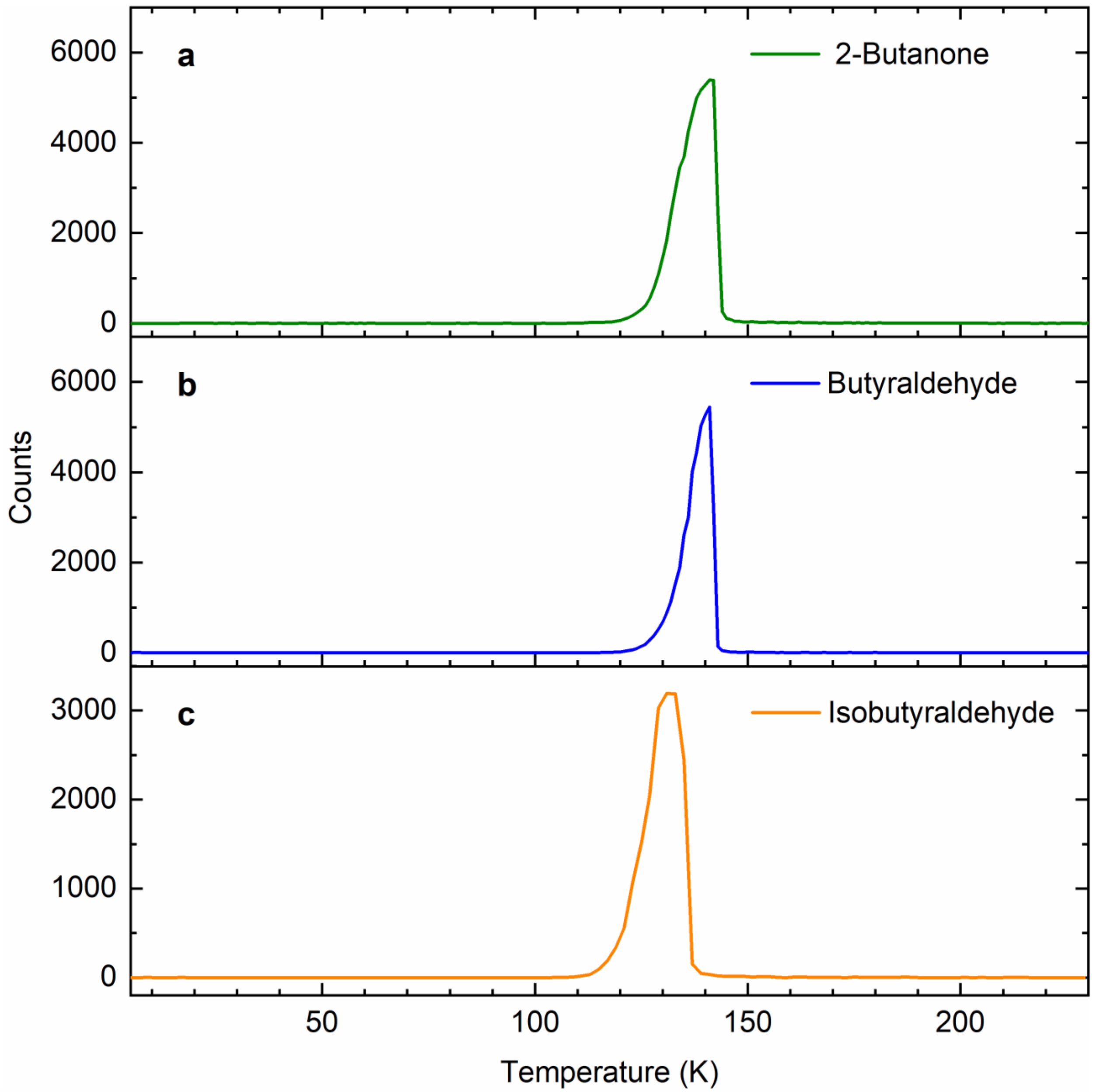

Figure S7. TPD profiles of *m/z* = 72 for 2-butanone ice recorded at 10.49 eV (a), butyraldehyde ice recorded at 10.49 eV (b), and isobutyraldehyde ice recorded at 9.92 eV (c).

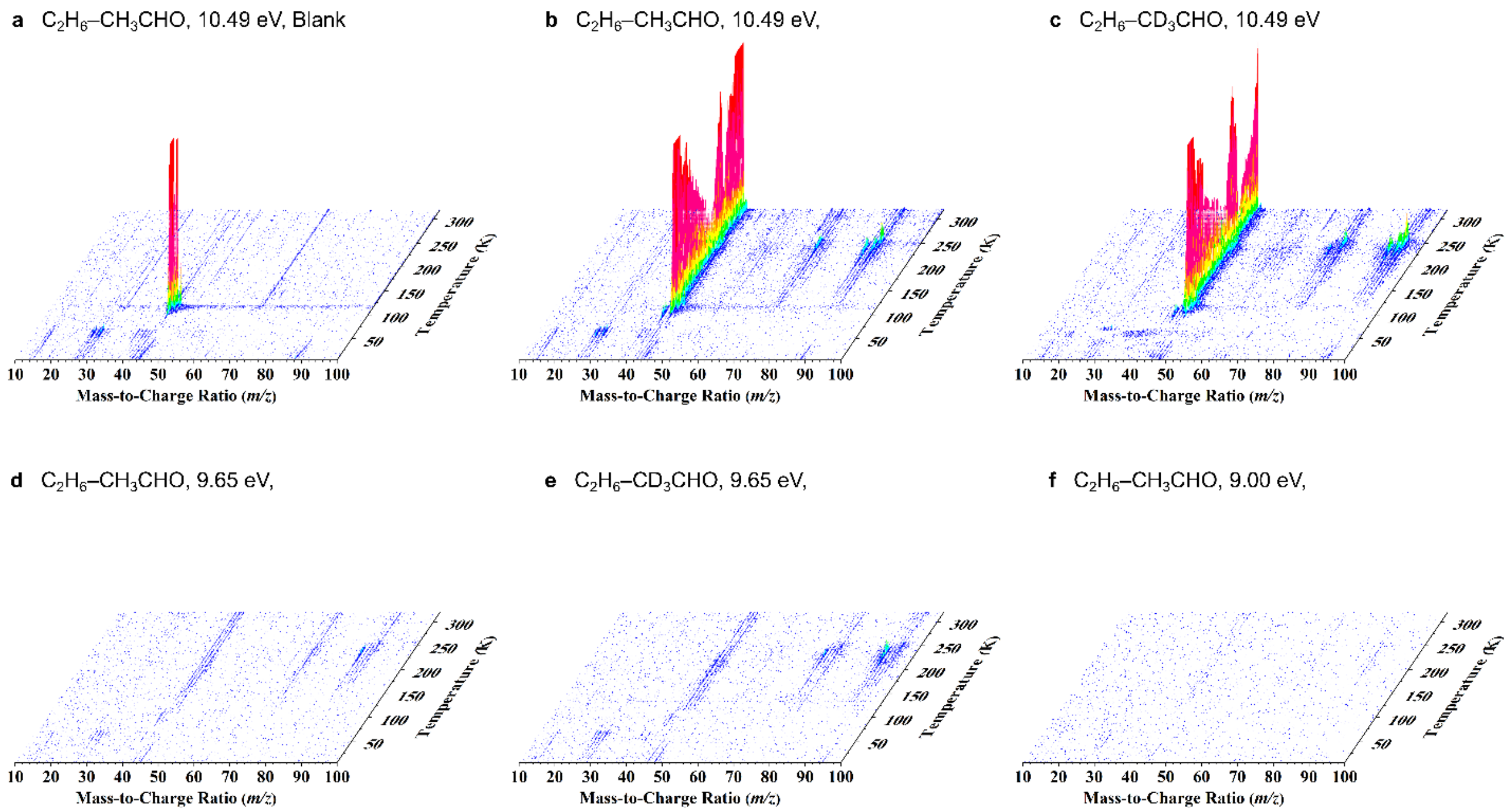

Figure S8. PI-ReToF-MS data during TPD of ethane–acetaldehyde ices with low dose (20 nA, 5 minutes) irradiation. Data were recorded for the unirradiated (blank) C_2H_6–CH_3CHO ice at 10.49 eV (a), the irradiated C_2H_6–CH_3CHO ice at 10.49 eV (b), 9.65 eV (d), and 9.00 eV (f), and the irradiated C_2H_6–CD_3CHO ice at 10.49 eV (c) and 9.65 eV (e).

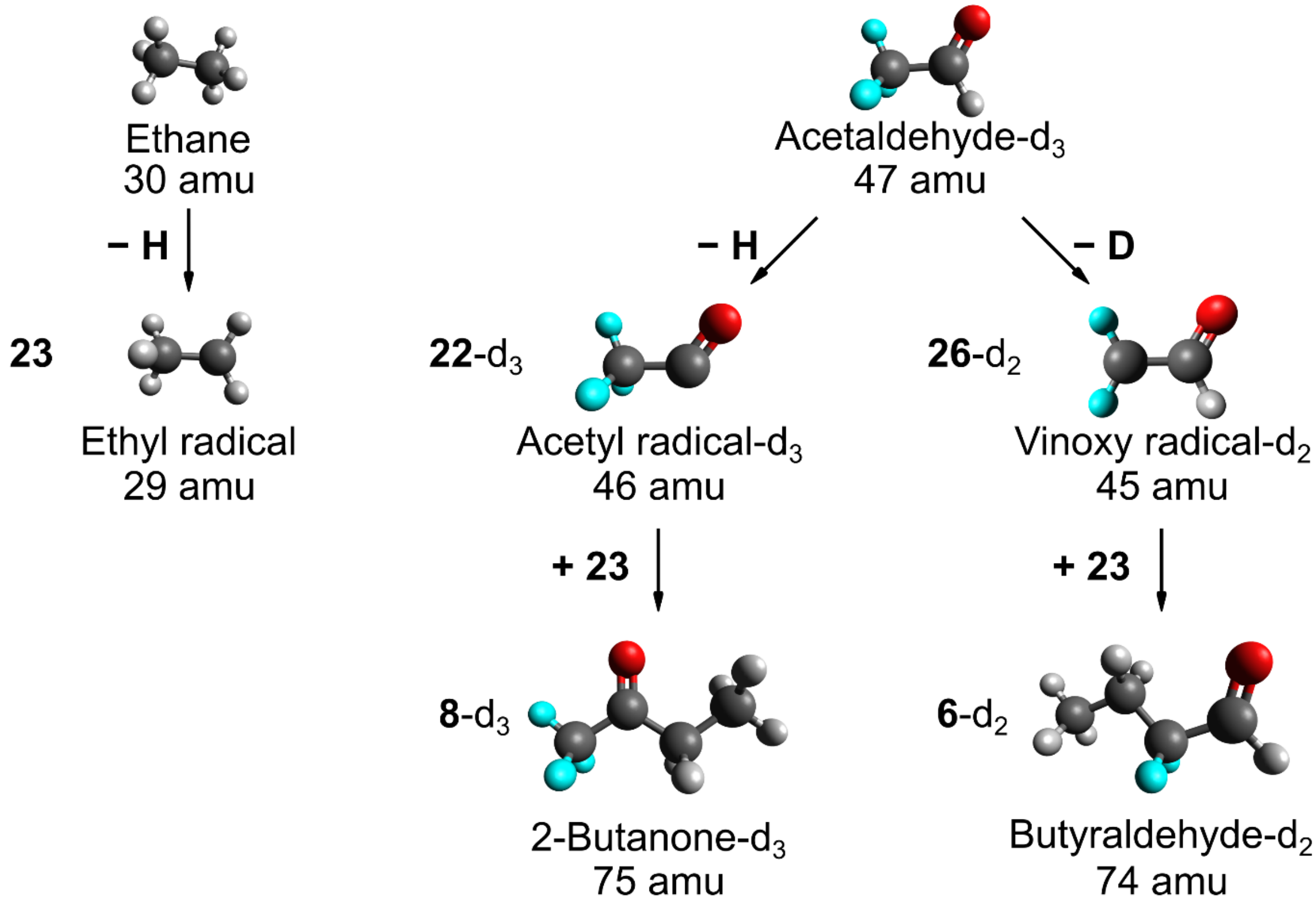

Figure S9. Proposed formation pathways of isomers **6** and **8** via radical-radical reactions in irradiated C_2H_6−CD_3CHO ices. The atoms are color-coded in white (hydrogen), cyan (deuterium), gray (carbon), red (oxygen).

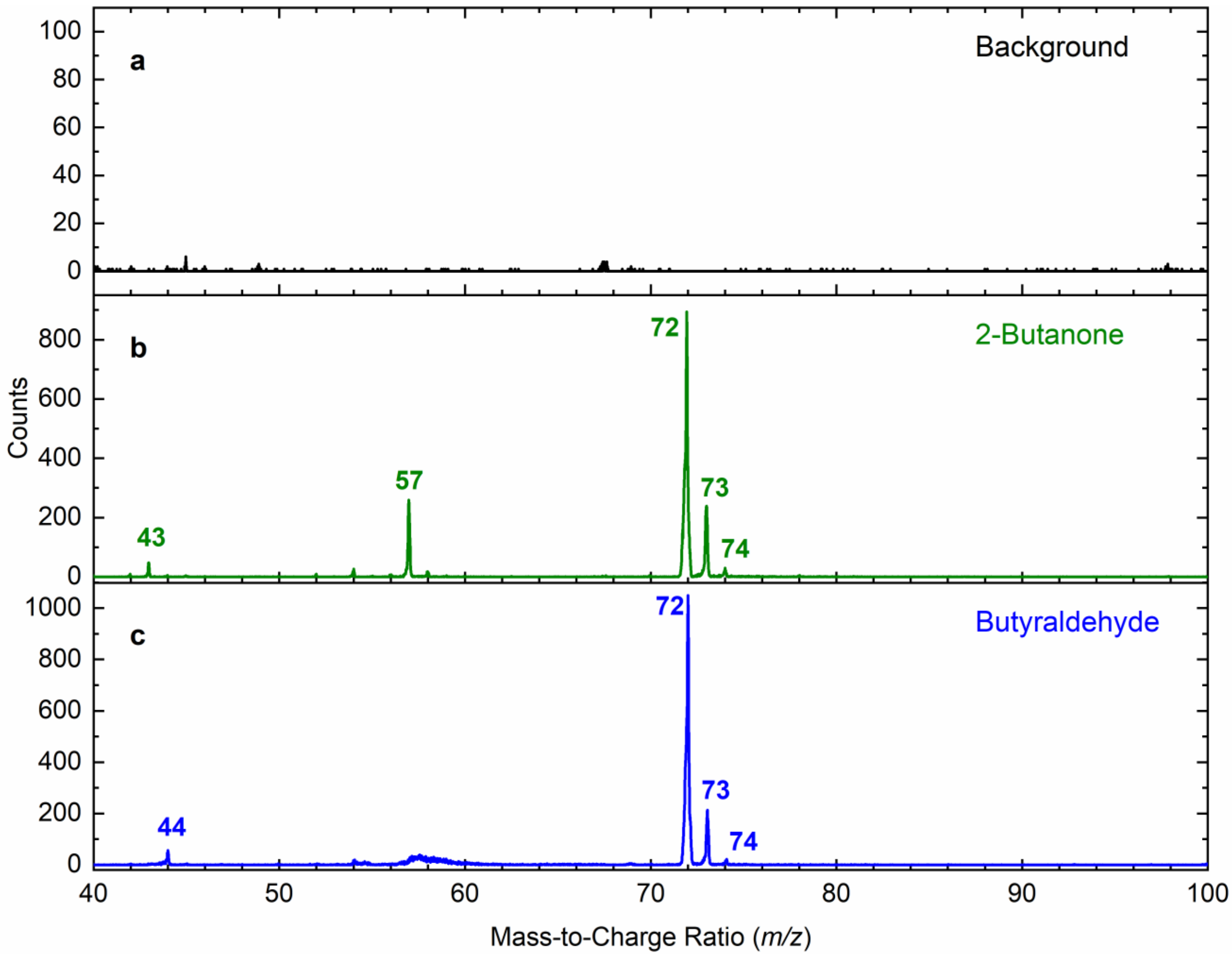

Figure S10. Gas phase mass spectra of 2-butanone (*m/z* = 72) and butyraldehyde (*m/z* = 72) samples recorded at 10.49 eV. Mass spectra were collected for background gases with 9000 VUV laser pulses (a), 2-butanone at a pressure of 1.5 $\times 10^{-8}$ Torr with 5400 laser pulses (b), and butyraldehyde at a pressure of 2 $\times 10^{-8}$ Torr with 9000 laser pulses (c).

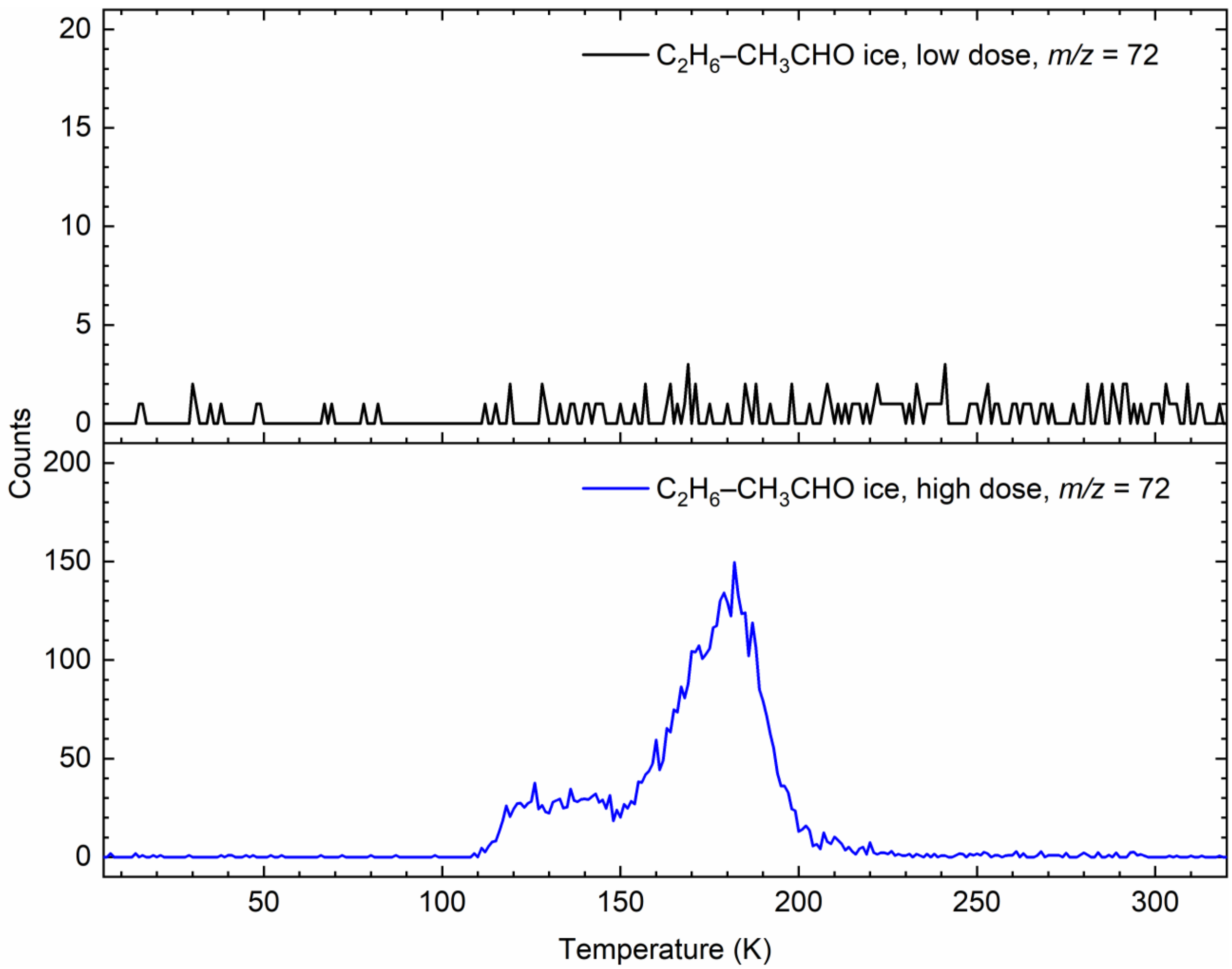

Figure S11. TPD profiles of *m/z* = 72 recorded at 9.00 eV in irradiated C_2H_6–CH_3CHO ices under low dose (20 nA, 5 minutes) and high dose (106 nA, 60 minutes) irradiation conditions.

Table S1. Absorption peaks observed in CO−C_3H_8 ice before and after irradiation (20 nA, 5 minutes) at 5 K. For consistency of notation in this work, the fundamental modes of C_3H_8 are labeled as ν_1–ν_{12} based on the selected bands reported by Hudson et al. Vibration mode: stretching (ν), bending (δ), wagging (ω), twisting (τ), rocking (ρ). Indication: asymmetric (a) and symmetric (s).

Pristine ice, absorptions before irradiation (cm^{-1})		
**CO**	Assignment[18]	
4246	2ν(CO)	
2135	ν(CO)	
2089	$\nu(^{13}CO)$	
**C_3H_8**	Assignment[7,19,20]	
2961, 2937, 2914, 2896	ν_1	ν(CH)
2874	ν_2	ν(CH)
2840	$\nu_3 + \nu_5$	combination
2814	$\nu_3 + \nu_6$	combination (tentative)
2787	$2\nu_4$	combination (tentative)
2762	$\nu_4 + \nu_5$	combination (tentative)
2735	$2\nu_5$	combination (tentative)
2726, 2716	$\nu_4 + \nu_6$	combination (tentative)
2699	$\nu_5 + \nu_6$	combination (tentative)
2655	$\nu_3 + \nu_7$	combination
2490	$\nu_6 + \nu_8$	combination
2434	$\nu_4 + \nu_9$	combination
2026	$\nu_8 + \nu_{11}$	combination
1933	$\nu_7 + \nu_{12}$	combination
1468	ν_3	$\delta(CH_2) + \delta(CH_3)$
1386	ν_4	$\omega(CH_3$/CH, in-phase)
1371	ν_5	$\omega(CH_3$/CH, out-of-phase)
1334	ν_6	$\omega(CH_2$/CH)
1188	ν_7	$\rho(CH_3/CH_2)$
1157	ν_8	$\omega(CH_3)$
1051	ν_9	ν_a(CC)
921	ν_{10}	$\rho(CH_3)$
870	ν_{11}	ν_s(CC)
748	ν_{12}	$\tau(CH_3/CH_2) + \rho(CH_3/CH_2)$
New absorptions after irradiation (cm^{-1})	Assignment[21,22]	
1796	$\nu_3(H\dot{C}O)$	

Table S2. Absorption peaks observed in C_2H_6–CH_3CHO ice before and after low dose irradiation (20 nA, 5 minutes) at 5 K. Vibration mode: stretching (*ν*), rocking (*ρ*), bending in plane (*δ*), and out of plane (*γ*). Indication: asymmetric (*a*) and symmetric (*s*).

Pristine ice, absorptions observed before irradiation (cm^{-1})		
**C_2H_6**	Assignment[13,23–32]	
4401	$\nu_8 + \nu_{10}$	combination
4360	$\nu_2 + \nu_7$	combination
4322	$\nu_6 + \nu_{10}$	combination
4272	$\nu_1 + \nu_6$	combination
4250	$\nu_2 + \nu_5$	combination
4175, 4162	$\nu_7 + \nu_{12}$	combination
4126, 4107	$\nu_8 + \nu_{11} + \nu_{12}$	combination
4073	$\nu_5 + \nu_{12}$	combination
3261	$\nu_4 + \nu_7$	combination
2973	ν_{10}	$\nu(CH_3)$
2956	ν_1	$\nu_s(CH_3)$
2941, 2908	$\nu_8 + \nu_{11}$	combination
2880	ν_5	$\nu_s(CH_3)$
2849	$\nu_2 + \nu_4 + \nu_{12}$	combination
2821	$\nu_6 + \nu_{11}$	combination
2737	$\nu_2 + \nu_6$	combination
2647	$\nu_8 + \nu_{12}$	combination
2559	$\nu_6 + \nu_9$	combination
1463	ν_{11}	$\nu(CH_3)$
1370	ν_6	$\nu_s(CH_3)$
820	ν_{12}	$\rho(CH_3)$
**CH_3CHO**	Assignment[21,33–35]	
3426	$2\nu_4$	overtone
3002	ν_1	$\nu_a(CH_3)$
2963	ν_{11}	$\nu(CH_3)$
2919	ν_2	$\nu_s(CH_3)$
2864, 2832	$2\nu_6$	overtone
2753, 2724	ν_3	$\nu(CH)$
2594	$\nu_4 + \nu_9$	combination
2457	$\nu_7 + \nu_8$	combination
2230	$\nu_7 + \nu_9$	combination
2003	$\nu_8 + \nu_9$	combination
1762	$2\nu_9$	overtone
1723	ν_4	$\nu(CO)$
1685	ν_4 ($CH_3{}^{13}CHO$)	$\nu({}^{13}CO)$
1631	$\nu_8 + \nu_{10}$	combination
1541	$2\nu_{14}$	overtone
1425	ν_5 / ν_{12}	$\delta(CH_3)$ / $\delta_a(CH_3)$
1403	$\nu_9 + \nu_{10}$	combination

1387	ν_6	$\delta(CH)$
1345	ν_7	$\delta_s(CH_3)$
1118	ν_8	$\gamma(CH_3)$
1105	ν_8 ($^{13}CH_3CHO$)	$\gamma(^{13}CH_3)$
882	$\nu_{14} + \nu_{15}$	combination
770	ν_{14}	$\gamma(CH)$

New absorptions observed after irradiation (cm^{-1})	Assignment[11,18,36]
2130	$\nu(CO)$
1839	$\nu_3(CH_3\dot{C}O)$
1570	$\nu_4(\dot{C}H_2CHO)$ (tentative)

Table S3. Absorption peaks observed in C_2H_6−CH_3CHO ice before and after high dose irradiation (106 nA, 60 minutes) at 5 K. Vibration mode: stretching (*ν*), rocking (*ρ*), bending in plane (*δ*), and out of plane (*γ*). Indication: asymmetric (*a*) and symmetric (*s*).

Pristine ice, absorptions observed before irradiation (cm^{-1})		
**C_2H_6**	Assignment[13,23–32]	
4402	$\nu_8 + \nu_{10}$	combination
4361	$\nu_2 + \nu_7$	combination
4322	$\nu_6 + \nu_{10}$	combination
4274	$\nu_1 + \nu_6$	combination
4248	$\nu_2 + \nu_5$	combination
4177, 4163	$\nu_7 + \nu_{12}$	combination
4126, 4098	$\nu_8 + \nu_{11} + \nu_{12}$	combination
4068	$\nu_5 + \nu_{12}$	combination
3260	$\nu_4 + \nu_7$	combination
2973	ν_{10}	$\nu(CH_3)$
2957	ν_1	$\nu_s(CH_3)$
2941, 2911	$\nu_8 + \nu_{11}$	combination
2880	ν_5	$\nu_s(CH_3)$
2848	$\nu_2 + \nu_4 + \nu_{12}$	combination
2821	$\nu_6 + \nu_{11}$	combination
2726	$\nu_2 + \nu_6$	combination
2648	$\nu_8 + \nu_{12}$	combination
2559	$\nu_6 + \nu_9$	combination
1463	ν_{11}	$\nu(CH_3)$
1371	ν_6	$\nu_s(CH_3)$
821	ν_{12}	$\rho(CH_3)$
**CH_3CHO**	Assignment[21,33–35]	
3426	$2\nu_4$	overtone
3005	ν_1	$\nu_a(CH_3)$
2985	ν_{11}	$\nu(CH_3)$
2924	ν_2	$\nu_s(CH_3)$
2862, 2832	$2\nu_6$	overtone
2754, 2738	ν_3	$\nu(CH)$
2594	$\nu_4 + \nu_9$	combination
2457	$\nu_7 + \nu_8$	combination
2230	$\nu_7 + \nu_9$	combination
2012	$\nu_8 + \nu_9$	combination
1760	$2\nu_9$	overtone
1720	ν_4	$\nu(CO)$
1688	ν_4 ($CH_3{}^{13}CHO$)	$\nu(^{13}CO)$
1645	$\nu_8 + \nu_{10}$	combination
1428	ν_5 / ν_{12}	$\delta(CH_3)$ / $\delta_a(CH_3)$
1398	$\nu_9 + \nu_{10}$	combination
1386	ν_6	$\delta(CH)$

1347	ν_7	$\delta_s(CH_3)$
1120	ν_8	$\gamma(CH_3)$
769	ν_{14}	$\gamma(CH)$

New absorptions observed after irradiation (cm^{-1})	Assignment[13,18,21,27,29,31,32,35]
4496	$\nu_2 + \nu_3(CH_4)$
3314	$\nu_3(C_2H_2)$ (tentative)
3213	$\nu_3 + \nu_4 + \nu_5(C_2H_2)$ (tentative)
3092	$\nu_9(C_2H_4)$
2338	$\nu_3(CO_2)$
2133	$\nu(CO)$
1301	$\nu_4(CH_4)$
954	$\nu_7(C_2H_4)$

Table S4. Absorption peaks observed in C_2H_6–CD_3CHO ice before and after low dose irradiation (23 nA, 5 minutes) at 5 K. Vibration mode: stretching (*ν*), rocking (*ρ*), bending in plane (*δ*), and out of plane (*γ*). Indication: asymmetric (*a*) and symmetric (*s*).

Pristine ice, absorptions observed before irradiation (cm^{-1})		
**C_2H_6**	Assignment[13,23–32]	
4399	$\nu_8 + \nu_{10}$	combination
4359	$\nu_2 + \nu_7$	combination
4322	$\nu_6 + \nu_{10}$	combination
4274	$\nu_1 + \nu_6$	combination
4249	$\nu_2 + \nu_5$	combination
4179, 4163	$\nu_7 + \nu_{12}$	combination
4125	$\nu_8 + \nu_{11} + \nu_{12}$	combination
4067	$\nu_5 + \nu_{12}$	combination
3262	$\nu_4 + \nu_7$	combination
2973	ν_{10}	$\nu(CH_3)$
2957	ν_1	$\nu_s(CH_3)$
2942, 2918	$\nu_8 + \nu_{11}$	combination
2880	ν_5	$\nu_s(CH_3)$
2843	$\nu_2 + \nu_4 + \nu_{12}$	combination
2825	$\nu_6 + \nu_{11}$	combination
2739	$\nu_2 + \nu_6$	combination
2649	$\nu_8 + \nu_{12}$	combination
2558	$\nu_6 + \nu_9$	combination
1464	ν_{11}	$\nu(CH_3)$
1371	ν_6	$\nu_s(CH_3)$
821	ν_{12}	$\rho(CH_3)$
**CD_3CHO**	Assignment[21,33,35,37,38]	
3421	$2\nu_4$	overtone
3108	$\nu_4 + \nu_5$	combination
2269	$2\nu_6$	overtone
2252	ν_2	$\nu_a(CD_3)$
2224	ν_{11}	$\nu(CD_3)$
2191	$\nu_6 + \nu_{12}$	combination
2115	ν_3	$\nu_s(CD_3)$
2082	$\nu_6 + \nu_8$	combination
1723	ν_4	$\nu(CO)$
1680	ν_4 ($CD_3{}^{13}CHO$)	$\nu(^{13}CO)$
1392	ν_5	$\delta(CD)$
1134	ν_6	$\nu(CC)$
1026	ν_7	$\delta_a(CD_3)$
963	ν_8	$\delta_s(CD_3)$
New absorptions observed after irradiation (cm^{-1})	Assignment[18,21,22,35,36,39]	
2137	$\nu(CO)$	

2096	$\nu_2(CD_2CO)$
1849	$\nu_3(CD_3\dot{C}O)$

Table S5. Error analysis of adiabatic ionization energies (IEs) and relative energies (ΔE) of 2-butanone (**8**) conformers; IEs and ΔE were computed at the composite CBS-QB3 level including the zero-point vibrational energy corrections. The IE ranges are corrected for the thermal and Stark effect by −0.03 eV and the combined error limits of −0.05/+0.05 eV.

Conformer	Structure	ΔE ($kJ\ mol^{-1}$)	Computed IE (eV)	Corrected IE ranges (eV)
**8a**	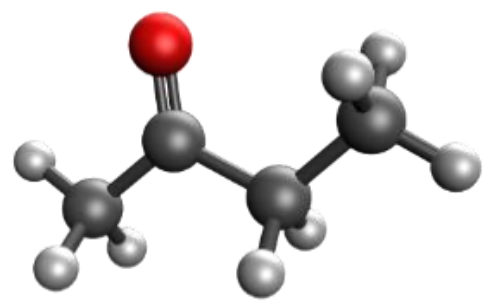	0.0	9.58	9.50 – 9.60
**8b**	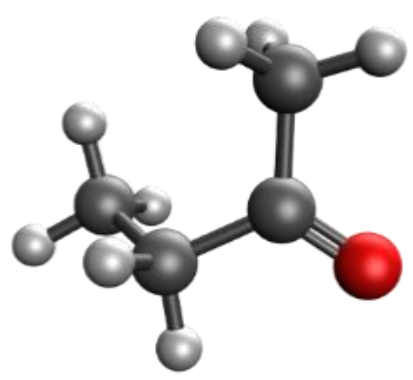	6.3	9.51	9.43 – 9.53

Table S6. Error analysis of IEs and relative energies (ΔE) of butyraldehyde (**6**) conformers; IEs and ΔE were computed at the composite CBS-QB3 level including the zero-point vibrational energy corrections. The IE ranges are corrected for the thermal and Stark effect by −0.03 eV and the combined error limits of −0.05/+0.05 eV.

Conformer	Structure	ΔE (kJ mol^{-1})	Computed IE (eV)	Corrected IE ranges (eV)
**6a**	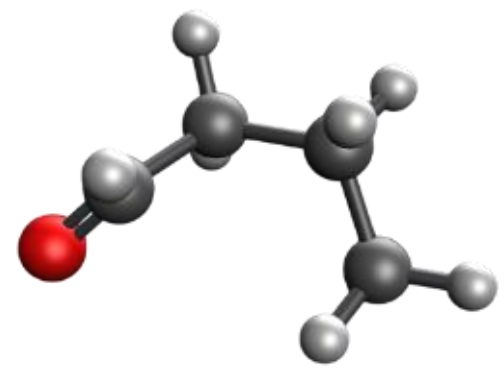	0.0	9.85	9.77 – 9.87
**6b**	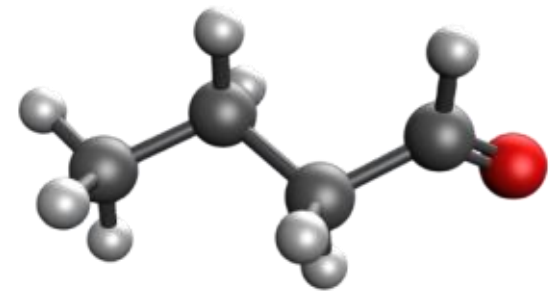	0.4	9.89	9.81 – 9.91
**6c**	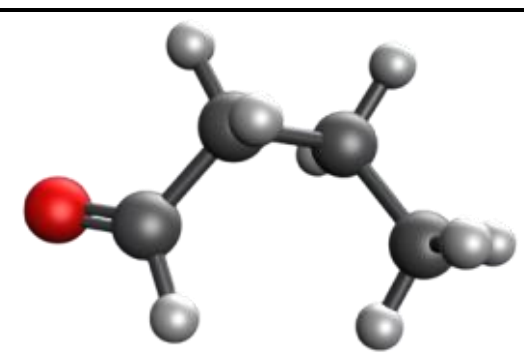	5.3	9.82	9.74 – 9.84

Table S7. Error analysis of IEs and relative energies (ΔE) of 1-buten-2-ol (**28**) conformers; IEs and ΔE were computed at the composite CBS-QB3 level including the zero-point vibrational energy corrections. The IE ranges are corrected for the thermal and Stark effect by −0.03 eV and the combined error limits of −0.05/+0.05 eV.

Conformer	Structure	ΔE (kJ mol^{-1})	Computed IE (eV)	Corrected IE ranges (eV)
**28a**	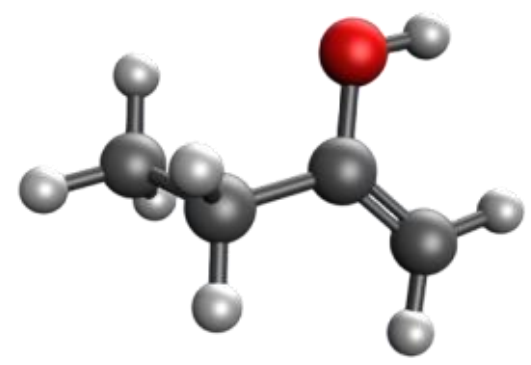	0.0	8.80	8.72 – 8.82
**28b**	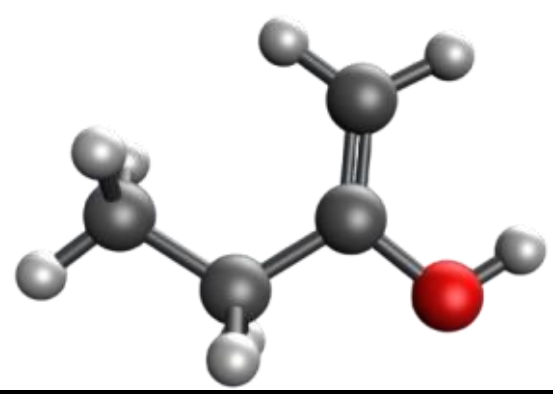	0.2	8.79	8.71 – 8.81
**28c**	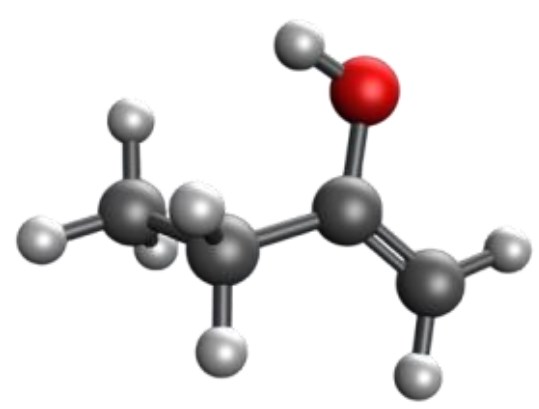	7.6	8.60	8.52 – 8.62

Table S8. Error analysis of IEs and relative energies (ΔE) of 2-buten-2-ol (**29**) conformers; IEs and ΔE were computed at the composite CBS-QB3 level including the zero-point vibrational energy corrections. The IE ranges are corrected for the thermal and Stark effect by −0.03 eV and the combined error limits of −0.05/+0.05 eV.

Conformer	Structure	ΔE (kJ mol^{-1})	Computed IE (eV)	Corrected IE ranges (eV)
**29a**	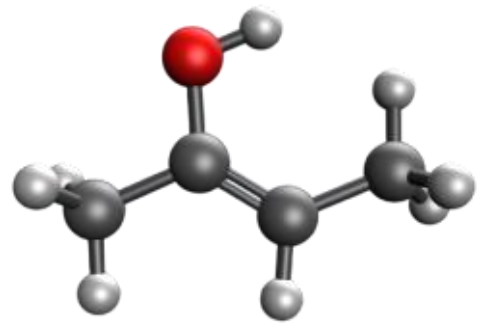	0.0	8.86	8.78 – 8.88
**29b**	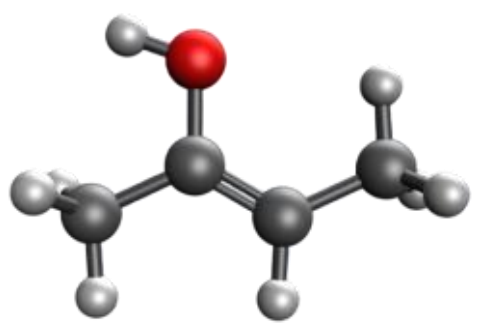	3.6	8.30	8.22 – 8.32
**29c**	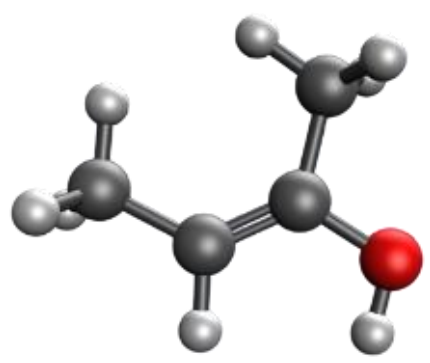	4.6	8.37	8.29 – 8.39
**29d**	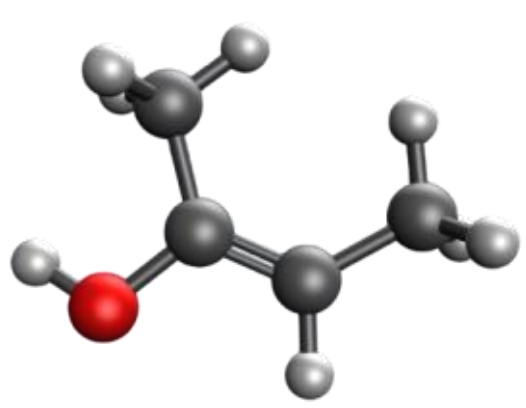	11.0	8.26	8.18 – 8.28

Table S9. Error analysis of IEs and relative energies (ΔE) of 1-buten-1-ol (**27**) conformers; IEs and ΔE were computed at the composite CBS-QB3 level including the zero-point vibrational energy corrections. The IE ranges are corrected for the thermal and Stark effect by −0.03 eV and the combined error limits of −0.05/+0.05 eV.

Conformer	Structure	ΔE (kJ mol^{-1})	Computed IE (eV)	Corrected IE ranges (eV)
**27a**	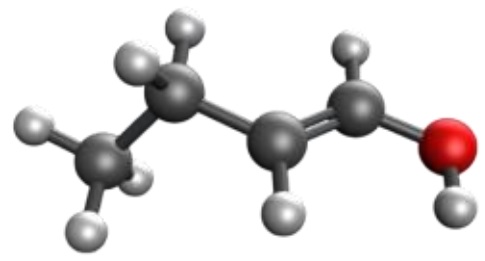	0.0	8.71	8.63 – 8.73
**27b**	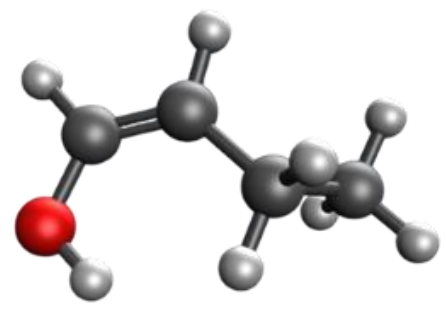	0.0	8.72	8.64 – 8.74
**27c**	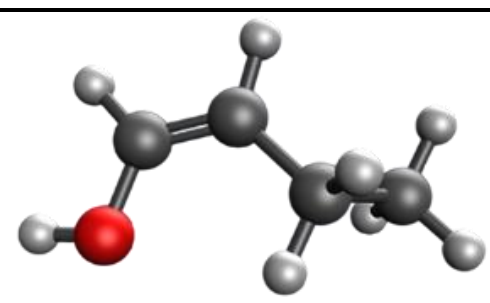	2.3	8.61	8.53 – 8.63
**27d**	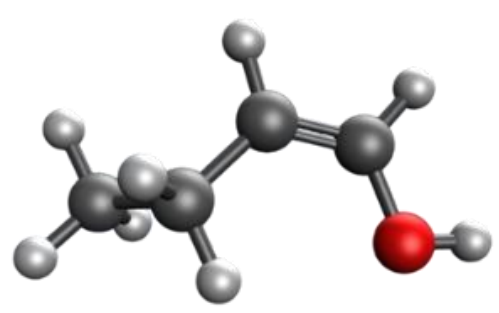	2.3	8.61	8.53 – 8.63
**27e**	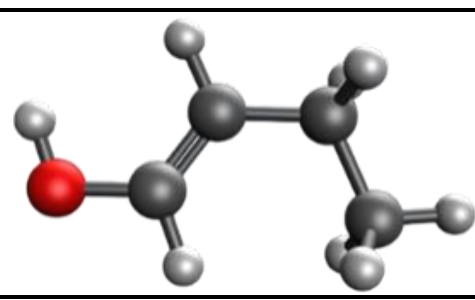	2.5	8.68	8.60 – 8.70
**27f**	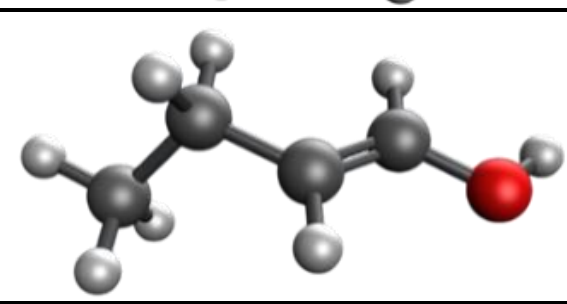	4.3	8.59	8.51 – 8.61
**27g**	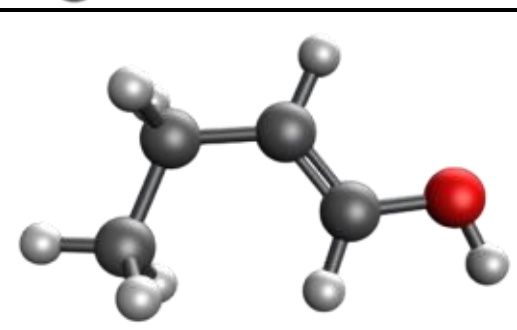	6.9	8.56	8.48 – 8.58
**27h**	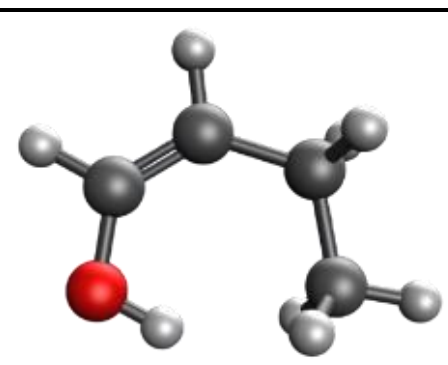	9.6	8.62	8.54 – 8.64

Table S10. Error analysis of IEs and relative energies (ΔE) of 2-methylprop-1-en-1-ol (**25**) conformers; IEs and ΔE were computed at the composite CBS-QB3 level including the zero-point vibrational energy corrections. The IE ranges are corrected for the thermal and Stark effect by −0.03 eV and the combined error limits of −0.05/+0.05 eV.

Conformer	Structure	ΔE (kJ mol^{-1})	Computed IE (eV)	Corrected IE ranges (eV)
**25a**	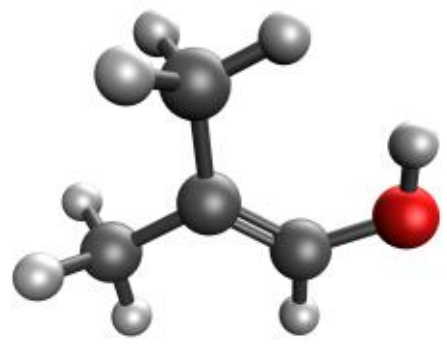	0.0	8.39	8.31 – 8.41
**25b**	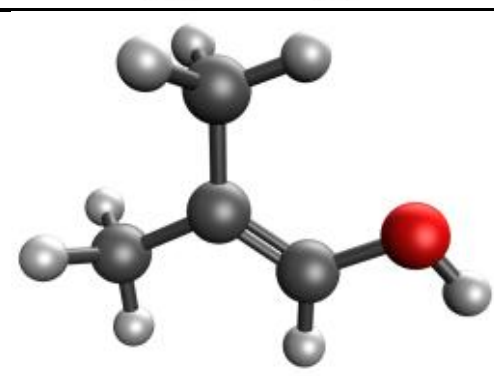	1.0	8.27	8.19 – 8.29

Table S11. Parameters used to calculate the yields of isobutyraldehyde (**7**) in carbon monoxide−propane ice and 2-butanone (**8**) in ethane−acetaldehyde ice.

	$CO-C_3H_8$	$C_2H_6-CH_3CHO$
Photoionization energy (eV)	9.92	10.49
Irradiation current (nA)	19 ± 2	20 ± 2
Irradiation time (s)	300 ± 10	300 ± 10
Total number of electrons	$(3.56 \pm 0.51) \times 10^{13}$	$(3.75 \pm 0.51) \times 10^{13}$
Initial kinetic energy of the electrons, E_{init} (eV)	5000	5000
Average kinetic energy of backscattered electrons, E_{bs}* (eV)	$(3.16 \pm 0.32) \times 10^{3}$	$(3.11 \pm 0.31) \times 10^{3}$
Average kinetic energy of transmitted electrons, E_{trans}* (eV)	$(6.77 \pm 0.68) \times 10^{2}$	$(5.67 \pm 0.57) \times 10^{2}$
Fraction of backscattered electrons, f_{bs}*	$(3.10 \pm 0.31) \times 10^{-1}$	$(2.98 \pm 0.30) \times 10^{-1}$
Fraction of transmitted electrons, f_{trans}*	$(9.20 \pm 0.20) \times 10^{-4}$	$(3.60 \pm 0.36) \times 10^{-4}$
Average penetration depth (nm)	313 ± 31	338 ± 34
Deposition depth of 99% electron energy (nm)	659 ± 22	532 ± 17
Total number of product molecules	$(5.93 \pm 1.33) \times 10^{15}$	$(8.78 \pm 1.76) \times 10^{14}$
Total deposited energy (eV)	$(1.43 \pm 0.21) \times 10^{17}$	$(1.53 \pm 0.21) \times 10^{17}$
Product yield (molecules eV^{-1})	$(4.14 \pm 1.11) \times 10^{-2}$	$(5.75 \pm 1.40) \times 10^{-3}$

*****Values derived from CASINO simulations

Table S12. Experimental conditions of carbon monoxide−propane ices and isobutyraldehyde ($(CH_3)_2CHCHO$) ice including composition, ice thickness, irradiation parameters, and VUV photon energies.

Exp.	Ice	Composition of carbon monoxide to propane	Thickness (nm)	Irradiation current (nA)	Irradiation time (s)	Dose (eV/ carbon monoxide)	Dose (eV/ propane)	Photon energy (eV)
1	$CO−C_3H_8$	1 : 1.1 ± 0.2	717 ± 24	–	–	–	–	9.92
2	$CO−C_3H_8$	1 : 1.1 ± 0.2	717 ± 24	19 ± 2	300 ± 10	0.18 ± 0.03	0.28 ± 0.05	9.92
3	$CO−C_3H_8$	1 : 1.1 ± 0.2	717 ± 24	20 ± 2	300 ± 10	0.18 ± 0.03	0.29 ± 0.05	9.72
4	$CO−C_3H_8$	1 : 1.1 ± 0.2	717 ± 24	20 ± 2	300 ± 10	0.18 ± 0.03	0.29 ± 0.05	9.34
5	$CO−C_3H_8$	1 : 1.0 ± 0.2	717 ± 24	23 ± 2	300 ± 10	0.21 ± 0.03	0.33 ± 0.05	8.45
6	$CO−C_3H_8$	1 : 1.1 ± 0.2	717 ± 24	20 ± 2	300 ± 10	0.18 ± 0.03	0.29 ± 0.05	8.17
7	$(CH_3)_2CHCHO$	–	230 ± 23	–	–	–	–	9.92

Table S13. Experimental conditions of ethane−acetaldehyde ices, and butyraldehyde ($CH_3CH_2CH_2CHO$) as well as 2-butanone ($CH_3CH_2COCH_3$) ices including composition, ice thickness, irradiation parameters, and VUV photon energies.

Exp.	Ice	Composition of ethane to acetaldehyde	Thickness (nm)	Irradiation current (nA)	Irradiation time (s)	Dose (eV/ ethane)	Dose (eV/ acetaldehyde)	Photon energy (eV)
1	C_2H_6−CH_3CHO	1.6 ± 0.4 : 1	719 ± 24	−	−	−	−	10.49
2	C_2H_6−CH_3CHO	1.5 ± 0.3 : 1	719 ± 24	20 ± 2	300 ± 10	0.20 ± 0.03	0.30 ± 0.05	10.49
3	C_2H_6−CH_3CHO	1.5 ± 0.3 : 1	719 ± 24	21 ± 2	300 ± 10	0.21 ± 0.03	0.31 ± 0.05	9.65
4	C_2H_6−CH_3CHO	1.5 ± 0.3 : 1	719 ± 24	20 ± 2	300 ± 10	0.20 ± 0.03	0.30 ± 0.05	9.00
5	C_2H_6−CH_3CHO	1.7 ± 0.4 : 1	719 ± 24	106 ± 2	3600 ± 10	13 ± 2	19 ± 2	9.00
6	C_2H_6−CD_3CHO	1.5 ± 0.3 : 1	719 ± 24	23 ± 2	300 ± 10	0.22 ± 0.03	0.34 ± 0.05	10.49
7	C_2H_6−CD_3CHO	1.6 ± 0.4 : 1	719 ± 24	22 ± 2	300 ± 10	0.21 ± 0.03	0.33 ± 0.05	9.65
8	$CH_3CH_2CH_2CHO$	−	120 ± 24	−	−	−	−	10.49
9	$CH_3CH_2COCH_3$	−	120 ± 24	−	−	−	−	10.49

Table S14. Vacuum ultraviolet (VUV) light generation parameters. The uncertainty of VUV photon energies is less than 0.001 eV.

VUV photon energy (eV)	Nonlinear medium in four-wave mixing	ω_1 laser wavelength (nm)	ω_1 Dye	ω_2 laser wavelength (nm)	ω_2 Dye
10.49 ($3\omega_1$)	Xenon	355	–	–	–
9.92 ($2\omega_1 - \omega_2$)	Krypton	212.556	Stilbene 420	710.092	LDS 722
9.72 ($2\omega_1 - \omega_2$)	Krypton	212.556	Stilbene 420	637.113	DCM
9.65 ($2\omega_1 - \omega_2$)	Krypton	212.556	Stilbene 420	614.992	Rhodamine 610 and 640
9.34 ($2\omega_1 - \omega_2$)	Krypton	212.556	Stilbene 420	532	–
9.00 ($2\omega_1 - \omega_2$)	Xenon	222.566	Coumarin 450	579.014	Pyrromethene 597
8.45 ($2\omega_1 - \omega_2$)	Krypton	202.316	Rhodamine 610 and 640	325.720	DCM in DMSO
8.17 ($2\omega_1 - \omega_2$)	Krypton	212.556	Stilbene 420	355	–

Table S15. Cartesian coordinates (Å), and harmonic frequencies (cm^{-1}) of isomer **8** (2-butanone) calculated at the composite CBS-QB3 level of theory.

8a

Geometry

```
H     0.5829780000   -1.4652520000    0.7977540000
H     0.6120910000   -1.3678170000   -0.9404750000
H     2.1040000000    0.5649670000    0.9445040000
H     2.1194820000    0.6744730000   -0.8091620000
H     2.8527180000   -0.7270230000   -0.0089440000
H    -2.0205580000   -1.0384140000    0.9645240000
H    -1.9809370000   -1.2406590000   -0.7831320000
H    -2.6721510000    0.2499970000   -0.0785290000
C     0.6841240000   -0.7546540000   -0.0324740000
C     2.0202440000   -0.0202580000    0.0266310000
C    -0.5280740000    0.1696470000   -0.0105610000
C    -1.8902810000   -0.5020190000    0.0187980000
O    -0.4142130000    1.3741780000   -0.0126140000
```

Frequency

```
2.927899999999999991e+01 1.001761000000000053e+02 1.945894999999999868e+02
2.485027000000000044e+02 4.002049999999999841e+02 4.751800999999999817e+02
5.904356999999999971e+02 7.552508000000000266e+02 7.603469999999999800e+02
9.382609999999999673e+02 9.532623999999999569e+02 9.991779999999999973e+02
1.105321099999999888e+03 1.131845399999999927e+03 1.185603699999999890e+03
1.283064200000000028e+03 1.368013799999999947e+03 1.386595499999999902e+03
1.418040099999999939e+03 1.454043400000000020e+03 1.468121799999999894e+03
1.478399400000000014e+03 1.493959000000000060e+03 1.502602699999999913e+03
1.797439399999999978e+03 3.007812199999999848e+03 3.028591100000000097e+03
3.031713499999999840e+03 3.041887600000000020e+03 3.084657799999999952e+03
3.105787499999999909e+03 3.113189499999999953e+03 3.138126400000000103e+03

5.635629999999999740e+01 1.205386000000000024e+02 2.285278000000000134e+02
2.415922999999999945e+02 3.399703000000000088e+02 4.043960000000000150e+02
4.701952999999999747e+02 5.667904999999999518e+02 7.422314000000000078e+02
8.071706000000000358e+02 9.393029000000000224e+02 9.669049999999999727e+02
1.015108799999999974e+03 1.063169900000000098e+03 1.078183899999999994e+03
1.247178900000000112e+03 1.278032400000000052e+03 1.335788099999999986e+03
1.406001800000000003e+03 1.426544599999999946e+03 1.428459800000000087e+03
1.451262500000000045e+03 1.456143399999999929e+03 1.491997399999999971e+03
1.694856399999999894e+03 3.008336800000000039e+03 3.030215599999999995e+03
3.034613100000000031e+03 3.080246200000000044e+03 3.101421600000000126e+03
3.122944100000000162e+03 3.142071300000000065e+03 3.177424399999999878e+03
```

8b

Geometry

```
H    -0.9252270000   -1.6110420000    0.3807400000
```

H	-0.9018080000	-0.2815180000	1.5436160000
H	-1.8062050000	0.0064650000	-1.3861750000
H	-1.8284900000	1.2970400000	-0.1762530000
H	-2.8830980000	-0.1030760000	0.0070980000
H	0.7144650000	1.7579370000	0.9857820000
H	0.5769290000	1.7860690000	-0.7691430000
H	2.1231170000	1.3216680000	-0.0303440000
C	-0.8059240000	-0.5305220000	0.4789740000
C	-1.8893840000	0.2150660000	-0.3160910000
C	0.6211120000	-0.2041260000	0.0529690000
C	1.0404800000	1.2569390000	0.0697550000
O	1.3915760000	-1.0747100000	-0.2836200000

Frequency

4.980570000000000164e+01 1.144433999999999969e+02 2.203262000000000000e+02
2.533352999999999895e+02 4.055738999999999805e+02 5.179808000000000447e+02
5.697600999999999658e+02 7.410864000000000260e+02 7.862301999999999680e+02
9.288060000000000400e+02 9.634896999999999707e+02 9.987241999999999962e+02
1.096725500000000011e+03 1.124846299999999928e+03 1.225368099999999913e+03
1.287161499999999933e+03 1.338972800000000007e+03 1.383584599999999909e+03
1.414874700000000075e+03 1.466082100000000082e+03 1.469076900000000023e+03
1.483705999999999904e+03 1.506270500000000084e+03 1.507532099999999900e+03
1.794542500000000018e+03 3.013835799999999836e+03 3.029640400000000227e+03
3.032296899999999823e+03 3.083206999999999880e+03 3.087179299999999785e+03
3.097079400000000078e+03 3.105826500000000124e+03 3.138295999999999822e+03

6.819939999999999714e+01 1.097541999999999973e+02 2.200146000000000015e+02
2.468294999999999959e+02 3.543965999999999781e+02 3.893469000000000051e+02
5.314086999999999534e+02 5.788139999999999645e+02 7.872912000000000035e+02
8.705384000000000242e+02 9.240416000000000167e+02 9.687628999999999451e+02
1.018367700000000013e+03 1.045245400000000018e+03 1.088701999999999998e+03
1.231775499999999965e+03 1.285947899999999890e+03 1.344801200000000108e+03
1.391052799999999934e+03 1.424678200000000061e+03 1.442260800000000017e+03
1.448217799999999897e+03 1.462332799999999907e+03 1.491429000000000087e+03
1.693346000000000004e+03 3.009587500000000091e+03 3.027438900000000103e+03
3.035252100000000155e+03 3.090947500000000218e+03 3.120394899999999780e+03
3.134147899999999936e+03 3.151309900000000198e+03 3.173313799999999901e+03

Table S16. Cartesian coordinates (Å), and harmonic frequencies (cm^{-1}) of isomer **6** (butyraldehyde) calculated at the composite CBS-QB3 level of theory.

6a

Geometry

H	1.0726890000	-1.6029690000	0.3902810000
H	2.7374570000	-1.0176530000	0.3370950000
H	1.7690480000	-1.0719170000	-1.1401500000
H	1.9514320000	1.2818120000	-0.2468850000
H	1.2565660000	0.7690080000	1.2748060000
H	-0.2231610000	0.6121070000	-1.4279090000
H	-0.3859120000	1.9154110000	-0.2374560000
H	-1.2140680000	0.2068970000	1.4759380000
C	1.7341970000	-0.8614150000	-0.0672460000
C	1.2510720000	0.5680310000	0.1967110000
C	-0.1558120000	0.8485510000	-0.3628000000
C	-1.2340710000	0.0819220000	0.3671350000
O	-2.0670450000	-0.6144030000	-0.1535650000

Frequency

6.358400000000000318e+01 1.194591999999999956e+02 2.176475000000000080e+02
3.026324000000000183e+02 3.966277999999999793e+02 5.101068999999999960e+02
7.605404999999999518e+02 7.755149999999999864e+02 8.998553000000000566e+02
9.454012000000000171e+02 9.786703999999999724e+02 1.058246399999999994e+03
1.124838199999999915e+03 1.157306399999999940e+03 1.236636500000000069e+03
1.279916899999999941e+03 1.348481099999999969e+03 1.374256100000000060e+03
1.418583499999999958e+03 1.422610300000000052e+03 1.462726000000000113e+03
1.493202199999999948e+03 1.502357199999999921e+03 1.506482299999999896e+03
1.817142200000000003e+03 2.832874499999999898e+03 3.005243699999999990e+03
3.017427299999999832e+03 3.025287200000000212e+03 3.053586499999999887e+03
3.081337700000000041e+03 3.089277700000000095e+03 3.093164400000000114e+03

1.755647999999999911e+02 1.865055999999999869e+02 2.362839999999999918e+02
2.972765999999999735e+02 3.988331000000000017e+02 5.718644000000000460e+02
7.296928000000000338e+02 7.606196999999999662e+02 7.803532000000000153e+02
8.638889000000000351e+02 9.223432999999999993e+02 9.518101000000000340e+02
1.056073599999999942e+03 1.087420100000000048e+03 1.117652199999999993e+03
1.144388699999999972e+03 1.206451099999999997e+03 1.264632399999999961e+03
1.286409900000000107e+03 1.357586399999999912e+03 1.388252400000000080e+03
1.410753999999999905e+03 1.424450399999999945e+03 1.470176899999999932e+03
1.703693899999999985e+03 2.687229200000000219e+03 2.867674599999999828e+03
2.912864300000000185e+03 2.987384500000000116e+03 3.052746799999999894e+03
3.075297300000000178e+03 3.096357700000000023e+03 3.118565700000000106e+03

6b

Geometry

H	-2.4493210000	-1.0165240000	-0.7528850000

H	-3.1782900000	0.5483490000	-0.3791130000
H	-2.6251700000	-0.5038670000	0.9284920000
H	-0.9977380000	1.3789150000	0.5211200000
H	-0.8255840000	0.8776710000	-1.1512980000
H	0.1759490000	-1.3135350000	-0.4177110000
H	-0.0752470000	-0.8373590000	1.2732860000
H	1.5233750000	1.1136710000	0.9517390000
C	-2.3951940000	-0.1540390000	-0.0824390000
C	-1.0142160000	0.5040400000	-0.1391720000
C	0.1191210000	-0.4582080000	0.2601760000
C	1.4656960000	0.2243750000	0.2811470000
O	2.4249480000	-0.1180410000	-0.3614870000

Frequency

6.77972000000000368e+01 9.70225999999999707e+01 2.35235999999999900e+02
2.54616999999999902e+02 3.80305999999999832e+02 5.15176100000000195e+02
7.48894599999999685e+02 8.05801600000000076e+02 9.02894000000000055e+02
9.52640899999999878e+02 1.00428290000000041e+03 1.04427739999999943e+03
1.13608919999999892e+03 1.16166570000000015e+03 1.25258200000000107e+03
1.28608330000000008e+03 1.32904430000000021e+03 1.38003040000000100e+03
1.41579970000000030e+03 1.42244650000000015e+03 1.46720970000000112e+03
1.49561959999999991e+03 1.50121129999999937e+03 1.50996630000000047e+03
1.81697499999999909e+03 2.83996779999999897e+03 2.99963110000000060e+03
3.01668930000000003e+03 3.02387460000000100e+03 3.04535269999999913e+03
3.08212690000000205e+03 3.09028540000000209e+03 3.09230580000000090e+03

8.35123999999999952e+01 9.67088999999999986e+01 2.25879199999999973e+02
2.43826500000000100e+02 3.23527699999999816e+02 4.20724100000000213e+02
6.72230999999999945e+02 7.66790700000000153e+02 8.18572000000000027e+02
8.81901399999999669e+02 9.07798499999999900e+02 9.21952599999999613e+02
1.01071820000000024e+03 1.04560570000000070e+03 1.15968679999999948e+03
1.20551099999999967e+03 1.25445900000000060e+03 1.25821669999999946e+03
1.29280449999999962e+03 1.37794890000000094e+03 1.42098240000000098e+03
1.45204250000000018e+03 1.48988020000000059e+03 1.49900459999999982e+03
1.74878400000000106e+03 2.91696759999999948e+03 3.00661790000000191e+03
3.02791030000000007e+03 3.05553029999999897e+03 3.09880960000000046e+03
3.10351670000000128e+03 3.13770559999999777e+03 3.14736360000000133e+03

6c

Geometry

H	2.2656170000	-0.5452290000	1.0989640000
H	2.9310670000	-0.6309320000	-0.5339070000
H	1.5100500000	-1.5815620000	-0.1150130000
H	0.8701470000	0.4730480000	-1.4532810000
H	1.6863360000	1.4896990000	-0.2872000000
H	-0.6823290000	1.6619820000	0.1753760000

H	0.0560840000	0.7270550000	1.4914050000
H	-0.8445160000	-1.4123600000	0.4573090000
C	2.0007010000	-0.6172730000	0.0395160000
C	1.1180120000	0.5602750000	-0.3898960000
C	-0.1881750000	0.7190790000	0.4203600000
C	-1.1846530000	-0.3902720000	0.1779420000
O	-2.2834710000	-0.2265690000	-0.2901480000

Frequency

3.247059999999999746e+01 1.061085000000000065e+02 2.301628000000000043e+02
2.835058999999999969e+02 4.012791000000000281e+02 5.026032999999999902e+02
7.498345000000000482e+02 8.210701000000000249e+02 8.738509000000000242e+02
9.544566999999999553e+02 9.813468000000000302e+02 1.052659300000000030e+03
1.134433099999999968e+03 1.147087299999999914e+03 1.248022200000000112e+03
1.286860400000000027e+03 1.342806000000000040e+03 1.373207400000000007e+03
1.419477399999999989e+03 1.428860300000000052e+03 1.468449499999999944e+03
1.496332200000000057e+03 1.503813100000000077e+03 1.510558199999999943e+03
1.811467599999999948e+03 2.862821600000000217e+03 3.004066299999999956e+03
3.024623799999999846e+03 3.027387499999999818e+03 3.055234800000000178e+03
3.087064600000000155e+03 3.089004300000000057e+03 3.094730700000000070e+03

9.056879999999999598e+01 1.155495999999999981e+02 2.106913000000000125e+02
2.719434999999999718e+02 3.803376999999999839e+02 4.423539000000000101e+02
6.643710999999999558e+02 7.491588000000000420e+02 8.188440000000000509e+02
8.401957999999999629e+02 9.169492000000000189e+02 9.927780999999999949e+02
1.028429900000000089e+03 1.057488900000000058e+03 1.132711600000000089e+03
1.161588700000000017e+03 1.204135700000000043e+03 1.281717699999999923e+03
1.335665700000000015e+03 1.401156500000000051e+03 1.416203999999999951e+03
1.436087199999999939e+03 1.455648500000000013e+03 1.482575100000000020e+03
1.769824399999999969e+03 2.856524399999999787e+03 2.905442399999999907e+03
2.966172300000000178e+03 3.009517400000000180e+03 3.061418299999999817e+03
3.066120699999999943e+03 3.110616100000000188e+03 3.142140100000000075e+03

Table S17. Cartesian coordinates (Å), and harmonic frequencies (cm^{-1}) of isomer **28** (1-buten-2-ol) calculated at the composite CBS-QB3 level of theory.

28a

Geometry

H	-2.8736970000	-0.2715570000	-0.0243510000
H	-1.7659680000	-0.4287820000	-1.3960340000
H	-1.8674250000	1.1003090000	-0.5130560000
H	-0.8087980000	-1.5470990000	0.6521950000
H	-0.9553490000	-0.0352020000	1.5443850000
H	2.5473680000	-0.5411060000	-0.5606160000
H	1.4142970000	-1.9626330000	-0.2122820000
H	1.6004850000	1.5425580000	-0.2233510000
C	-1.8897640000	0.0146160000	-0.4044550000
C	-0.7815760000	-0.4602610000	0.5489310000
C	0.5936580000	-0.0525280000	0.1027840000
C	1.5713860000	-0.8938790000	-0.2429570000
O	0.7183580000	1.3119780000	0.0884120000

Frequency

8.999240000000000350e+01 2.242813999999999908e+02 2.496568000000000040e+02
4.158639999999999759e+02 4.372604000000000042e+02 4.806351999999999975e+02
6.280336999999999534e+02 7.259125999999999976e+02 7.977541999999999689e+02
8.082788000000000466e+02 8.170435999999999694e+02 9.711014000000000124e+02
1.001886100000000056e+03 1.073167099999999891e+03 1.101641399999999976e+03
1.208527800000000070e+03 1.282929300000000012e+03 1.344603800000000092e+03
1.379707200000000057e+03 1.411380799999999908e+03 1.450551400000000058e+03
1.481855399999999918e+03 1.498093399999999974e+03 1.512898799999999937e+03
1.712991299999999910e+03 3.023407000000000153e+03 3.032765499999999975e+03
3.080208399999999983e+03 3.095137299999999868e+03 3.114006800000000112e+03
3.137472400000000107e+03 3.234527900000000045e+03 3.815988699999999881e+03

5.297670000000000101e+01 2.129496000000000038e+02 2.144115999999999929e+02
3.391376000000000204e+02 4.115305000000000177e+02 4.947948000000000093e+02
6.369287000000000489e+02 6.794945999999999913e+02 8.152939000000000078e+02
8.253162999999999556e+02 8.868162999999999556e+02 9.342380000000000564e+02
9.976957999999999629e+02 1.042958000000000084e+03 1.102082100000000082e+03
1.174649799999999914e+03 1.240875600000000077e+03 1.293826800000000048e+03
1.395216200000000072e+03 1.445964799999999968e+03 1.477803200000000061e+03
1.482521899999999960e+03 1.490285200000000032e+03 1.519084299999999985e+03
1.531091300000000047e+03 3.049535499999999956e+03 3.057065000000000055e+03
3.117958099999999831e+03 3.133115400000000136e+03 3.140217900000000100e+03
3.142575299999999970e+03 3.260467999999999847e+03 3.688003600000000006e+03

28b

Geometry

H	-2.8686070000	-0.5723350000	-0.0000160000

H	-2.0654990000	0.7275260000	-0.8843100000
H	-2.0654740000	0.7275570000	0.8842080000
H	-0.6848740000	-1.3967840000	-0.8719210000
H	-0.6849130000	-1.3968560000	0.8717810000
H	1.6642070000	1.8642520000	0.0000670000
H	-0.1766580000	2.0246830000	0.0000110000
H	2.4532830000	-0.2902870000	0.0000790000
C	-1.9989080000	0.0887260000	-0.0000410000
C	-0.7110590000	-0.7324310000	-0.0000430000
C	0.5709950000	0.0535330000	0.0000150000
C	0.6915340000	1.3831670000	0.0000310000
O	1.6391450000	-0.8057170000	0.0000410000

Frequency

9.279420000000000357e+01 2.546783000000000072e+02 2.748410000000000082e+02
4.283145000000000095e+02 4.608301000000000158e+02 4.972726000000000113e+02
4.986684999999999945e+02 7.256739000000000033e+02 7.978849999999999909e+02
8.118278000000000247e+02 8.149693999999999505e+02 9.749711999999999534e+02
1.021509800000000041e+03 1.085952500000000100e+03 1.119347400000000107e+03
1.185145500000000084e+03 1.289624800000000050e+03 1.322868600000000015e+03
1.405864900000000034e+03 1.419295200000000023e+03 1.449492199999999912e+03
1.480500399999999900e+03 1.501888300000000072e+03 1.513407999999999902e+03
1.713174700000000030e+03 3.018009199999999964e+03 3.033143700000000081e+03
3.039378600000000006e+03 3.097715400000000045e+03 3.098273400000000038e+03
3.142475100000000111e+03 3.245823300000000017e+03 3.815791400000000067e+03

5.320960000000000178e+01 2.129745000000000061e+02 2.144582000000000050e+02
3.391730000000000018e+02 4.115364999999999895e+02 4.947941999999999894e+02
6.369438000000000102e+02 6.794849000000000387e+02 8.153102999999999838e+02
8.253710999999999558e+02 8.867943999999999960e+02 9.340615000000000236e+02
9.976824000000000296e+02 1.042905500000000075e+03 1.102075100000000020e+03
1.174656400000000076e+03 1.240807600000000093e+03 1.293713199999999915e+03
1.395187699999999950e+03 1.445955099999999902e+03 1.477782099999999900e+03
1.482515400000000000e+03 1.490277800000000070e+03 1.519099699999999984e+03
1.531099899999999934e+03 3.049519600000000082e+03 3.057194800000000214e+03
3.118085900000000038e+03 3.133133200000000215e+03 3.140224999999999909e+03
3.142576599999999871e+03 3.260458000000000084e+03 3.688008400000000165e+03

28c

Geometry

H	-2.8511270000	-0.3681720000	-0.0210560000
H	-1.7281670000	-0.5143450000	-1.3841070000
H	-1.9192670000	1.0301570000	-0.5565420000
H	-0.7556330000	-1.5392270000	0.7032880000
H	-0.9434510000	-0.0137990000	1.5493320000
H	2.5612430000	-0.5006550000	-0.5602830000

H	1.4501410000	-1.9421990000	-0.1755900000
H	0.0770150000	1.7877860000	0.3783230000
C	-1.8783690000	-0.0524140000	-0.4056570000
C	-0.7518180000	-0.4568940000	0.5615010000
C	0.6179360000	-0.0349940000	0.0983640000
C	1.6006290000	-0.8734510000	-0.2292780000
O	0.8223720000	1.3208720000	-0.0103680000

Frequency

8.904470000000000596e+01 1.999770000000000039e+02 2.220459999999999923e+02
2.634601000000000113e+02 4.175203999999999951e+02 4.722945000000000277e+02
6.266276000000000295e+02 7.380805000000000291e+02 7.954909000000000106e+02
8.059189000000000078e+02 8.423206999999999880e+02 9.663799999999999955e+02
9.984162999999999784e+02 1.074223099999999931e+03 1.097824900000000071e+03
1.232432900000000018e+03 1.278211999999999989e+03 1.329678900000000112e+03
1.357552899999999909e+03 1.416085100000000011e+03 1.435752300000000105e+03
1.483302799999999934e+03 1.498511099999999942e+03 1.508058199999999943e+03
1.737615000000000009e+03 2.989452299999999923e+03 3.029273200000000088e+03
3.077059799999999996e+03 3.096518900000000031e+03 3.103895199999999932e+03
3.160599999999999909e+03 3.251574500000000171e+03 3.841960199999999986e+03

6.910020000000000095e+01 2.267190999999999974e+02 2.513877999999999986e+02
3.788303000000000225e+02 4.107848999999999933e+02 4.951315000000000168e+02
5.571385000000000218e+02 6.705851000000000113e+02 7.862626999999999953e+02
7.956598000000000184e+02 8.980818000000000438e+02 1.001315000000000055e+03
1.013970400000000041e+03 1.082153700000000072e+03 1.084243199999999888e+03
1.170952700000000050e+03 1.259577600000000075e+03 1.322969399999999951e+03
1.394538900000000012e+03 1.424435300000000097e+03 1.443198399999999992e+03
1.487711399999999912e+03 1.506044900000000098e+03 1.506957300000000032e+03
1.551577999999999975e+03 2.998156300000000101e+03 3.015021000000000186e+03
3.026162899999999809e+03 3.088903299999999945e+03 3.128458200000000033e+03
3.155030400000000100e+03 3.275723500000000058e+03 3.652105799999999817e+03

Table S18. Cartesian coordinates (Å), and harmonic frequencies (cm^{-1}) of isomer **29** (2-buten-2-ol) calculated at the composite CBS-QB3 level of theory.

**29a**<br>**Geometry**<br>H 2.6856970000 -0.5884630000 -0.7776910000<br>H 2.5827910000 -0.2843610000 0.9534410000<br>H 2.0928260000 0.9552630000 -0.1973460000<br>H 0.6003140000 -1.7781320000 0.0143740000<br>H -1.7759150000 -1.7062820000 -0.0052530000<br>H -2.3925990000 -0.2886670000 -0.8820750000<br>H -2.3950530000 -0.2939840000 0.8793050000<br>H 0.3314810000 1.7386380000 0.0245690000<br>C 2.0695460000 -0.1227510000 -0.0018070000<br>C 0.6803270000 -0.6971790000 0.0025910000<br>C -0.4658390000 -0.0062370000 0.0012360000<br>C -1.8320510000 -0.6178430000 -0.0015600000<br>O -0.5551800000 1.3637570000 -0.0015100000<br><br>**Frequency**<br>4.190570000000000306e+01 1.796681000000000097e+02 2.287023000000000081e+02<br>2.745158000000000129e+02 3.984350000000000023e+02 4.100658000000000243e+02<br>5.689971000000000458e+02 5.726284000000000560e+02 7.837079999999999700e+02<br>8.221992999999999938e+02 9.826163999999999987e+02 1.045674099999999953e+03<br>1.050934199999999919e+03 1.075185600000000022e+03 1.077414399999999887e+03<br>1.187927599999999984e+03 1.329444600000000037e+03 1.370011299999999892e+03<br>1.417707599999999957e+03 1.433166999999999916e+03 1.470906500000000051e+03<br>1.478497399999999971e+03 1.491873599999999897e+03 1.506735400000000027e+03<br>1.749006800000000112e+03 3.002485700000000179e+03 3.026499299999999948e+03<br>3.046715200000000095e+03 3.060036399999999958e+03 3.073750799999999799e+03<br>3.125247499999999945e+03 3.169916099999999915e+03 3.820940099999999802e+03<br><br>5.320409999999999684e+01 2.129736000000000047e+02 2.144556000000000040e+02<br>3.391761999999999944e+02 4.115359000000000265e+02 4.947916999999999916e+02<br>6.369411999999999807e+02 6.794876000000000431e+02 8.153088000000000193e+02<br>8.253682000000000016e+02 8.867930000000000064e+02 9.340739999999999554e+02<br>9.976814000000000533e+02 1.042908300000000054e+03 1.102074000000000069e+03<br>1.174657099999999900e+03 1.240806499999999915e+03 1.293725100000000111e+03<br>1.395190100000000029e+03 1.445956300000000056e+03 1.477779600000000073e+03<br>1.482514899999999898e+03 1.490277700000000095e+03 1.519098300000000108e+03<br>1.531100799999999936e+03 3.049518199999999979e+03 3.057175400000000081e+03<br>3.118081700000000183e+03 3.133131499999999960e+03 3.140223399999999856e+03<br>3.142577200000000175e+03 3.260458399999999983e+03 3.688006199999999808e+03
**29b**<br>**Geometry**<br>H 2.6541210000 -0.3715930000 -0.8798440000

H	2.6541420000	-0.3717320000	0.8796820000
H	2.0082170000	1.0210920000	0.0000380000
H	0.6519910000	-1.7768650000	-0.0001880000
H	-1.7304800000	-1.7410300000	-0.0001390000
H	-2.3748250000	-0.3496900000	-0.8844900000
H	-2.3747950000	-0.3498590000	0.8845040000
H	-1.3343190000	1.6812310000	0.0001260000
C	2.0755000000	-0.0670590000	-0.0000500000
C	0.7125360000	-0.6940290000	-0.0000850000
C	-0.4404910000	-0.0213660000	0.0000060000
C	-1.8010800000	-0.6534840000	-0.0000310000
O	-0.4291060000	1.3592580000	0.0001590000

Frequency

5.449360000000000070e+01 1.594384000000000015e+02 1.892838000000000136e+02
2.542632999999999868e+02 2.542844000000000051e+02 4.061886000000000081e+02
5.621848999999999705e+02 5.694184000000000196e+02 7.962143999999999551e+02
8.225827000000000453e+02 9.707197999999999638e+02 1.042984099999999899e+03
1.055650499999999965e+03 1.073139200000000073e+03 1.103968200000000024e+03
1.194671499999999924e+03 1.309544499999999971e+03 1.362833300000000008e+03
1.413374199999999973e+03 1.421426600000000008e+03 1.477966799999999921e+03
1.489481199999999944e+03 1.493121100000000069e+03 1.498315399999999954e+03
1.772610599999999977e+03 2.999778200000000197e+03 3.008368199999999888e+03
3.040190900000000056e+03 3.043689300000000003e+03 3.115969300000000203e+03
3.120817500000000109e+03 3.165893799999999828e+03 3.855172399999999925e+03

4.054569999999999652e+01 1.133542999999999950e+02 1.580883000000000038e+02
2.673184999999999718e+02 4.164486999999999739e+02 5.314563000000000557e+02
5.703038999999999987e+02 6.332178999999999860e+02 7.686055000000000064e+02
8.124483000000000175e+02 9.563614999999999782e+02 1.000003299999999967e+03
1.032607899999999972e+03 1.034987499999999955e+03 1.144700900000000047e+03
1.197292899999999918e+03 1.322169499999999971e+03 1.355459599999999909e+03
1.392298399999999901e+03 1.429486900000000105e+03 1.452834000000000060e+03
1.460592399999999998e+03 1.460785300000000007e+03 1.510042300000000068e+03
1.596373499999999922e+03 2.981965200000000095e+03 3.006429599999999937e+03
3.015407000000000153e+03 3.065655400000000100e+03 3.153077200000000175e+03
3.160220100000000002e+03 3.187544600000000173e+03 3.717084299999999985e+03

29c

Geometry

H	-2.6649870000	-0.2156500000	-0.8795840000
H	-1.9720670000	1.1448790000	0.0005720000
H	-2.6653950000	-0.2165180000	0.8790360000
H	-0.7889540000	-1.7318980000	-0.0000270000
H	-0.0672270000	2.0056940000	-0.0000100000
H	1.4213680000	1.6099910000	-0.8802800000

H	1.4213740000	1.6100030000	0.8802690000
H	1.3974570000	-1.7658050000	0.0000110000
C	-2.0710060000	0.0583880000	0.0000020000
C	-0.7426080000	-0.6423680000	-0.0000020000
C	0.4742220000	-0.0860590000	0.0000020000
C	0.8169090000	1.3713250000	-0.0000010000
O	1.6316670000	-0.8310520000	0.0000010000

Frequency

1.208564999999999969e+02 1.392869000000000028e+02 2.469950000000000045e+02
2.888000000000000114e+02 4.208450000000000273e+02 4.408460999999999785e+02
4.934306000000000267e+02 5.351113000000000284e+02 8.000375999999999976e+02
8.088098999999999705e+02 9.811799999999999500e+02 1.028009600000000091e+03
1.059979700000000093e+03 1.068588999999999942e+03 1.113108099999999922e+03
1.220939200000000028e+03 1.268107600000000048e+03 1.388603599999999915e+03
1.425543499999999995e+03 1.447594200000000001e+03 1.474094800000000077e+03
1.484463899999999967e+03 1.490175799999999981e+03 1.501854499999999916e+03
1.746887400000000071e+03 3.007422099999999773e+03 3.032088600000000042e+03
3.041463200000000143e+03 3.077601999999999862e+03 3.090483000000000175e+03
3.107826799999999821e+03 3.142484300000000076e+03 3.813019200000000183e+03

8.266140000000000043e+01 1.204813999999999936e+02 1.649074999999999989e+02
2.591732999999999834e+02 4.747436000000000149e+02 4.932744999999999891e+02
5.216381000000000085e+02 6.569349999999999454e+02 7.720044000000000324e+02
7.973669999999999618e+02 9.519115000000000464e+02 9.580574000000000296e+02
1.034852200000000039e+03 1.055175300000000107e+03 1.160988800000000083e+03
1.205533899999999903e+03 1.348492299999999886e+03 1.365503600000000006e+03
1.394482400000000098e+03 1.427709000000000060e+03 1.428330500000000029e+03
1.450503099999999904e+03 1.484411299999999983e+03 1.500229900000000043e+03
1.571897699999999986e+03 2.983865899999999783e+03 3.009839300000000094e+03
3.027686499999999796e+03 3.077739599999999882e+03 3.132708900000000085e+03
3.155047799999999825e+03 3.159452299999999923e+03 3.695038700000000063e+03

29d

Geometry

H	2.6660040000	-0.2068940000	0.8867840000
H	1.9737080000	1.1544130000	0.0073410000
H	2.6842780000	-0.1996630000	-0.8701030000
H	0.7963020000	-1.7349810000	-0.0226350000
H	0.0622120000	1.9925080000	-0.0375020000
H	-1.3802310000	1.6015560000	0.9084020000
H	-1.4483920000	1.5959200000	-0.8582720000
H	-2.3480510000	-0.4588350000	-0.1443300000
C	2.0781030000	0.0678600000	0.0031260000
C	0.7601710000	-0.6488410000	-0.0139940000
C	-0.4604880000	-0.1085990000	-0.0059420000

C	-0.8150520000	1.3496460000	0.0034870000
O	-1.5477790000	-0.9630520000	0.0262810000

Frequency

1.087368000000000023e+02 1.281266999999999996e+02 1.857827000000000055e+02
2.636754000000000246e+02 2.872887999999999806e+02 4.360613000000000170e+02
4.853439999999999941e+02 5.334303999999999633e+02 7.916549999999999727e+02
8.455268999999999551e+02 9.736615000000000464e+02 1.027761600000000044e+03
1.059841599999999971e+03 1.063532799999999952e+03 1.108592699999999923e+03
1.233045299999999997e+03 1.254256800000000112e+03 1.381597099999999955e+03
1.423460800000000063e+03 1.426101400000000012e+03 1.480514599999999973e+03
1.485036499999999933e+03 1.488350100000000111e+03 1.503348099999999931e+03
1.770434400000000096e+03 3.003203899999999976e+03 3.007341300000000047e+03
3.040679900000000089e+03 3.045482100000000173e+03 3.095018199999999979e+03
3.135651499999999942e+03 3.145747699999999895e+03 3.846773999999999887e+03

6.041649999999999920e+01 1.243983000000000061e+02 1.778077000000000112e+02
2.556093999999999937e+02 4.712493999999999801e+02 4.953768999999999778e+02
5.260407000000000153e+02 6.373038000000000238e+02 7.874262999999999693e+02
7.912349000000000387e+02 9.462096000000000231e+02 9.642845999999999549e+02
1.033559700000000021e+03 1.051756300000000010e+03 1.153670900000000074e+03
1.214177200000000084e+03 1.344963600000000042e+03 1.362544499999999971e+03
1.397378799999999956e+03 1.429330300000000079e+03 1.440181700000000092e+03
1.447715500000000020e+03 1.478348899999999958e+03 1.501081500000000005e+03
1.591419900000000098e+03 2.985608299999999872e+03 3.011347800000000007e+03
3.028319500000000062e+03 3.083351599999999962e+03 3.127598899999999958e+03
3.146456000000000131e+03 3.171038300000000163e+03 3.718946600000000217e+03

Table S19. Cartesian coordinates (Å), and harmonic frequencies (cm^{-1}) of isomer **27** (1-buten-1-ol) calculated at the composite CBS-QB3 level of theory.

27a

Geometry

H -3.2684480000 0.0916330000 -0.3746890000
H -2.4073340000 -1.3173890000 0.2536940000
H -2.0056440000 -0.6850830000 -1.3453840000
H -1.5832900000 0.8363370000 1.2900050000
H -1.1520070000 1.4419340000 -0.2970120000
H 0.1929530000 -1.0059960000 1.0415730000
H 1.2008020000 1.2074530000 -0.8431390000
H 2.4520520000 -0.9796320000 0.4213160000
C -2.2917580000 -0.3988730000 -0.3298490000
C -1.2350810000 0.5252680000 0.2966590000
C 0.1204490000 -0.1159980000 0.4153270000
C 1.2120660000 0.3296250000 -0.2054050000
O 2.4671080000 -0.2036750000 -0.1508450000

Frequency

1.018961999999999932e+02 1.952634999999999934e+02 2.135915999999999997e+02
3.201768999999999892e+02 4.100751000000000204e+02 4.604873000000000047e+02
5.332492999999999483e+02 7.825013999999999896e+02 8.257591999999999643e+02
9.113846999999999525e+02 9.659868999999999915e+02 1.015283900000000017e+03
1.085176500000000033e+03 1.130203500000000076e+03 1.142479000000000042e+03
1.248268800000000056e+03 1.298386400000000094e+03 1.335049099999999953e+03
1.350692500000000109e+03 1.407477100000000064e+03 1.411707599999999957e+03
1.485304699999999912e+03 1.497646199999999908e+03 1.507288000000000011e+03
1.728744899999999916e+03 2.999241700000000037e+03 3.020556099999999788e+03
3.040624699999999848e+03 3.082760000000000218e+03 3.091277599999999893e+03
3.101042699999999968e+03 3.174532700000000204e+03 3.806359800000000178e+03

5.089979999999999905e+01 1.602466000000000008e+02 1.997864999999999895e+02
2.840733000000000175e+02 3.788763000000000147e+02 5.180093000000000529e+02
6.405813000000000557e+02 7.692989999999999782e+02 8.552650999999999613e+02
9.049980000000000473e+02 9.648776000000000295e+02 9.946649999999999636e+02
1.023829700000000003e+03 1.147325299999999970e+03 1.189747399999999971e+03
1.202957699999999932e+03 1.276917699999999968e+03 1.318929499999999962e+03
1.371801999999999907e+03 1.398737399999999980e+03 1.418231199999999944e+03
1.461422000000000025e+03 1.487899100000000089e+03 1.497370100000000093e+03
1.606956100000000106e+03 2.945357700000000023e+03 3.053579000000000178e+03
3.095532200000000103e+03 3.130842500000000200e+03 3.136327999999999975e+03
3.140670099999999820e+03 3.187130500000000211e+03 3.680255999999999858e+03

27b

Geometry

H 2.9233000000 -0.9072250000 -0.0614980000

H 2.6032880000 0.7645950000 -0.5376810000
H 1.8173010000 -0.5578270000 -1.4019640000
H 1.3455840000 0.1249580000 1.5583520000
H 0.5610430000 -1.1720510000 0.6971980000
H 0.1848590000 1.8495370000 0.0557190000
H -2.0791150000 1.2775770000 -0.4563340000
H -1.3419850000 -1.3702170000 0.1673730000
C 2.1476860000 -0.2221990000 -0.4156500000
C 0.9678330000 -0.1605800000 0.5686260000
C -0.1041490000 0.8071850000 0.1456230000
C -1.3730580000 0.5154600000 -0.1482450000
O -1.9805180000 -0.7060670000 -0.1154120000

Frequency
3.816140000000000043e+01 2.134020999999999901e+02 2.468035999999999888e+02
2.894123000000000161e+02 4.035475000000000136e+02 5.473904999999999745e+02
6.461816999999999780e+02 7.614575999999999567e+02 7.935336999999999534e+02
8.779030000000000200e+02 9.608218000000000529e+02 1.010985800000000040e+03
1.052622399999999971e+03 1.091581200000000081e+03 1.141717499999999973e+03
1.248708100000000059e+03 1.290890900000000101e+03 1.331211800000000039e+03
1.361270099999999957e+03 1.409452999999999975e+03 1.437097099999999955e+03
1.488903999999999996e+03 1.497985599999999977e+03 1.509064800000000105e+03
1.724013599999999997e+03 2.994362500000000182e+03 3.017961499999999887e+03
3.023936299999999846e+03 3.086921200000000226e+03 3.094027999999999793e+03
3.154652799999999843e+03 3.193009100000000217e+03 3.810601999999999862e+03

4.819200000000000017e+01 1.878445000000000107e+02 2.027572999999999865e+02
2.735475000000000136e+02 3.730332000000000221e+02 6.394913000000000238e+02
6.572238999999999578e+02 7.694963999999999942e+02 8.240702999999999747e+02
8.910951000000000022e+02 9.522863999999999578e+02 9.949605000000000246e+02
1.033929200000000037e+03 1.088046499999999924e+03 1.179793899999999894e+03
1.207758000000000038e+03 1.248571099999999888e+03 1.304570699999999988e+03
1.398715599999999995e+03 1.405936599999999999e+03 1.443714600000000019e+03
1.488368699999999990e+03 1.489605399999999918e+03 1.511385999999999967e+03
1.586961800000000039e+03 2.961364799999999832e+03 3.052082199999999830e+03
3.055842200000000048e+03 3.136530799999999999e+03 3.140485599999999977e+03

27c
Geometry
H 2.9200730000 -0.9442220000 -0.0516110000
H 2.6446150000 0.7456590000 -0.4898750000
H 1.8509170000 -0.5403180000 -1.4039800000
H 1.3274140000 0.0761930000 1.5608100000
H 0.5131040000 -1.1744970000 0.6376100000
H 0.2172630000 1.8487630000 0.0344890000
H -2.0543400000 1.2886410000 -0.4504320000

H -2.7783280000 -0.7441540000 -0.3143100000
C 2.1654190000 -0.2344560000 -0.4026540000
C 0.9656480000 -0.1836450000 0.5573530000
C -0.0803820000 0.8092860000 0.1348240000
C -1.3501340000 0.5206120000 -0.1419950000
O -1.8555020000 -0.7533560000 -0.0509830000

Frequency
4.911260000000000048e+01 2.041196999999999946e+02 2.090523000000000025e+02
2.622948999999999842e+02 2.999898999999999774e+02 5.275960000000000036e+02
6.571147999999999456e+02 7.526938999999999851e+02 7.992582999999999629e+02
8.824796999999999798e+02 9.370363999999999578e+02 1.018808499999999981e+03
1.048252199999999903e+03 1.089791699999999992e+03 1.146320899999999938e+03
1.256287800000000061e+03 1.291733799999999974e+03 1.306806499999999915e+03
1.346522899999999936e+03 1.407767000000000053e+03 1.444289600000000064e+03
1.489892800000000079e+03 1.498625399999999900e+03 1.507894099999999980e+03
1.750158400000000029e+03 2.999609599999999773e+03 3.019870499999999993e+03
3.068690300000000207e+03 3.086553699999999935e+03 3.092760499999999865e+03
3.135848599999999806e+03 3.162367999999999938e+03 3.874195999999999913e+03

3.350379999999999825e+01 1.966584000000000003e+02 2.088941000000000088e+02
2.796716999999999871e+02 3.809970000000000141e+02 6.234779999999999518e+02
6.542815000000000509e+02 7.645614000000000487e+02 8.296199000000000296e+02
8.865788000000000011e+02 9.557386999999999944e+02 9.715149000000000115e+02
1.022260800000000017e+03 1.098060300000000097e+03 1.179286900000000060e+03
1.191265599999999949e+03 1.262005200000000059e+03 1.329063000000000102e+03
1.358100699999999961e+03 1.400221099999999979e+03 1.434886700000000019e+03
1.489125000000000000e+03 1.490462700000000041e+03 1.510807700000000068e+03
1.611524400000000014e+03 2.932362500000000182e+03 3.053327299999999923e+03
3.114346599999999853e+03 3.135198899999999867e+03 3.139654799999999796e+03
3.160742000000000189e+03 3.175768300000000181e+03 3.716579600000000028e+03

27d

Geometry
H -2.9200870000 -0.9442140000 -0.0516220000
H -1.8509600000 -0.5402460000 -1.4039950000
H -2.6446300000 0.7456880000 -0.4898060000
H -0.5131010000 -1.1745340000 0.6375330000
H -1.3273880000 0.0761120000 1.5608140000
H -0.2172750000 1.8487490000 0.0345240000
H 2.0543360000 1.2886620000 -0.4503970000
H 2.7783450000 -0.7441270000 -0.3143200000
C -2.1654370000 -0.2344350000 -0.4026470000
C -0.9656440000 -0.1836770000 0.5573360000
C 0.0803790000 0.8092720000 0.1348350000
C 1.3501360000 0.5206180000 -0.1419820000

O 1.8555180000 -0.7533460000 -0.0509980000

Frequency
4.911480000000000246e+01 2.041184000000000083e+02 2.090494999999999948e+02
2.622919999999999732e+02 2.999916000000000054e+02 5.275927000000000362e+02
6.571159999999999854e+02 7.526932000000000471e+02 7.992578999999999496e+02
8.824801999999999680e+02 9.370363999999999578e+02 1.018809300000000007e+03
1.048252099999999928e+03 1.089791899999999941e+03 1.146320999999999913e+03
1.256287800000000061e+03 1.291734099999999899e+03 1.306805699999999888e+03
1.346522500000000036e+03 1.407767200000000003e+03 1.444290099999999939e+03
1.489892399999999952e+03 1.498625299999999925e+03 1.507894000000000005e+03
1.750158899999999903e+03 2.999607399999999870e+03 3.019870499999999993e+03
3.068691100000000006e+03 3.086554399999999987e+03 3.092760400000000118e+03
3.135848399999999856e+03 3.162367299999999886e+03 3.874197700000000168e+03

3.350379999999999825e+01 1.966584000000000003e+02 2.088941000000000088e+02
2.796716999999999871e+02 3.809970000000000141e+02 6.234781000000000404e+02
6.542815000000000509e+02 7.645614000000000487e+02 8.296199000000000296e+02
8.865788000000000011e+02 9.557386999999999944e+02 9.715149000000000115e+02
1.022260800000000017e+03 1.098060300000000097e+03 1.179286900000000060e+03
1.191265499999999975e+03 1.262005200000000059e+03 1.329063000000000102e+03
1.358100699999999961e+03 1.400221099999999979e+03 1.434886600000000044e+03
1.489125000000000000e+03 1.490462700000000041e+03 1.510807700000000068e+03
1.611524400000000014e+03 2.932362399999999980e+03 3.053327299999999923e+03
3.114346599999999853e+03 3.135198899999999867e+03 3.139654799999999796e+03
3.160742000000000189e+03 3.175768300000000181e+03 3.716579600000000028e+03

27e
Geometry
H 3.0522640000 -0.6498620000 0.0000620000
H 1.6639050000 -1.2877500000 -0.8841980000
H 1.6638370000 -1.2878340000 0.8841610000
H 1.7081580000 1.2368120000 -0.8714740000
H 1.7081570000 1.2367570000 0.8716390000
H -0.6132120000 1.7222130000 0.0001990000
H -0.6300600000 -1.3519160000 -0.0002060000
H -2.6427000000 0.6177350000 0.0000240000
C 1.9617090000 -0.7170220000 0.0000200000
C 1.3381500000 0.6805100000 0.0000650000
C -0.1687350000 0.7264340000 0.0000700000
C -0.9831490000 -0.3280730000 -0.0000770000
O -2.3497750000 -0.3009060000 -0.0000850000

Frequency
1.346377999999999986e+02 2.074896999999999991e+02 2.231110999999999933e+02
3.058038999999999987e+02 3.988120999999999867e+02 4.545122000000000071e+02

5.953523000000000138e+02 7.612568999999999733e+02 8.394918000000000120e+02
8.915688999999999851e+02 9.694945999999999913e+02 1.025627500000000055e+03
1.094892599999999902e+03 1.108225300000000061e+03 1.142139300000000048e+03
1.253381000000000085e+03 1.293363399999999956e+03 1.338481399999999894e+03
1.375945699999999988e+03 1.397518299999999954e+03 1.420765900000000101e+03
1.489121499999999969e+03 1.501516599999999926e+03 1.512146300000000110e+03
1.732376700000000028e+03 2.992386300000000119e+03 3.005432600000000093e+03
3.025874800000000050e+03 3.086510600000000068e+03 3.093108299999999872e+03
3.100307299999999941e+03 3.197471100000000206e+03 3.805089899999999943e+03

1.272061000000000064e+02 1.696448000000000036e+02 1.958659000000000106e+02
2.562848999999999933e+02 4.030437999999999761e+02 5.883120999999999867e+02
6.344112999999999829e+02 6.522536000000000058e+02 8.653518000000000256e+02
8.902938000000000329e+02 9.953097000000000207e+02 1.012872499999999945e+03
1.044465200000000095e+03 1.113907200000000103e+03 1.175371799999999894e+03
1.231365299999999934e+03 1.292589999999999918e+03 1.334402399999999943e+03
1.375500999999999976e+03 1.389147400000000061e+03 1.404212099999999964e+03
1.436937699999999950e+03 1.502991899999999987e+03 1.507603299999999990e+03
1.610048500000000104e+03 2.923074799999999868e+03 2.932681500000000142e+03
3.044948699999999917e+03 3.109654500000000098e+03 3.130759000000000015e+03
3.132323100000000068e+03 3.203002300000000105e+03 3.678351900000000114e+03

27f

Geometry

H	-3.2712810000	0.0855400000	-0.4043910000
H	-2.4301420000	-1.2784320000	0.3412400000
H	-2.0090790000	-0.7794290000	-1.2987910000
H	-1.5926050000	0.9399190000	1.2124900000
H	-1.1413970000	1.4159660000	-0.4131110000
H	0.1773120000	-0.9383450000	1.1215240000
H	1.1993390000	1.1198480000	-0.9036590000
H	3.0763870000	0.0958750000	-0.5689090000
C	-2.3005630000	-0.4103030000	-0.3118650000
C	-1.2382480000	0.5488370000	0.2499030000
C	0.1054840000	-0.0981300000	0.4346730000
C	1.2074120000	0.2877410000	-0.2019140000
O	2.4183690000	-0.3287270000	-0.0138970000

Frequency

1.015811000000000064e+02 1.705508000000000095e+02 2.108285999999999945e+02
2.453714999999999975e+02 3.256390999999999849e+02 4.209239999999999782e+02
5.302143999999999551e+02 7.833234999999999673e+02 8.351518999999999551e+02
9.089062999999999874e+02 9.489065000000000509e+02 1.012888799999999947e+03
1.085355600000000095e+03 1.134399100000000089e+03 1.153117999999999938e+03
1.220403900000000021e+03 1.287644700000000057e+03 1.334702399999999898e+03
1.352686599999999999e+03 1.392676600000000008e+03 1.409235699999999952e+03

1.485612399999999980e+03 1.497230399999999918e+03 1.507808899999999994e+03
1.755300400000000081e+03 2.997071300000000065e+03 3.020992400000000089e+03
3.034487399999999980e+03 3.083853099999999813e+03 3.090617099999999937e+03
3.124234699999999975e+03 3.139421200000000226e+03 3.870729699999999866e+03

5.600310000000000343e+01 1.675799000000000092e+02 1.991475000000000080e+02
2.857176000000000045e+02 3.843784999999999741e+02 5.200313999999999623e+02
6.246635999999999740e+02 7.673678999999999633e+02 8.627073000000000320e+02
9.076747000000000298e+02 9.560421999999999798e+02 9.726559999999999491e+02
1.020671299999999974e+03 1.147434600000000046e+03 1.190837199999999939e+03
1.210287399999999934e+03 1.283995899999999892e+03 1.317311599999999999e+03
1.357342000000000098e+03 1.380894600000000082e+03 1.417159599999999955e+03
1.460558099999999968e+03 1.489372100000000046e+03 1.498492199999999912e+03
1.628270700000000033e+03 2.941542899999999918e+03 3.054186999999999898e+03
3.090943699999999808e+03 3.135768799999999828e+03 3.140434600000000046e+03
3.155154100000000199e+03 3.159576399999999921e+03 3.712895700000000033e+03

27g

Geometry

H	-3.0411510000	-0.7026880000	-0.0000230000
H	-1.6402050000	-1.3107240000	0.8845390000
H	-1.6401940000	-1.3107230000	-0.8845670000
H	-1.7375110000	1.2129440000	0.8709660000
H	-1.7374950000	1.2129430000	-0.8709950000
H	0.5849730000	1.7443220000	0.0000120000
H	0.6459090000	-1.3150010000	-0.0000020000
H	2.7758850000	-0.9675820000	0.0000080000
C	-1.9493550000	-0.7460850000	-0.0000160000
C	-1.3548320000	0.6642200000	-0.0000110000
C	0.1486810000	0.7483680000	0.0000030000
C	0.9871130000	-0.2837870000	0.0000060000
O	2.3500190000	-0.1074730000	0.0000210000

Frequency

1.390151999999999930e+02 1.729336999999999875e+02 2.068933000000000106e+02
2.532467000000000041e+02 3.194920999999999935e+02 3.948849000000000160e+02
5.950379000000000360e+02 7.608029999999999973e+02 8.448010000000000446e+02
8.864237000000000535e+02 9.538039999999999736e+02 1.023290700000000015e+03
1.091492199999999912e+03 1.107348700000000008e+03 1.158749600000000100e+03
1.217645299999999907e+03 1.293260899999999992e+03 1.349598400000000083e+03
1.364378699999999981e+03 1.384651800000000094e+03 1.416619500000000016e+03
1.488263400000000047e+03 1.501505400000000009e+03 1.511967399999999998e+03
1.757807000000000016e+03 2.992421600000000126e+03 3.004693400000000111e+03
3.023557299999999941e+03 3.082900700000000143e+03 3.091549799999999777e+03
3.140624800000000050e+03 3.150229800000000068e+03 3.871346599999999853e+03

1.331860000000000070e+02 1.748017000000000110e+02 1.943093000000000075e+02
2.579001999999999839e+02 4.026220000000000141e+02 5.940701999999999998e+02
6.129195999999999458e+02 6.626692000000000462e+02 8.739296000000000504e+02
8.867695999999999685e+02 9.560865000000000009e+02 1.010215299999999957e+03
1.046136199999999917e+03 1.110647699999999986e+03 1.183078299999999899e+03
1.235331500000000005e+03 1.307214899999999943e+03 1.323731199999999944e+03
1.364743600000000015e+03 1.374365500000000111e+03 1.401556100000000015e+03
1.435379300000000057e+03 1.503177699999999959e+03 1.508558800000000019e+03
1.629812400000000025e+03 2.927979100000000017e+03 2.936987399999999980e+03
3.043074599999999919e+03 3.106933799999999792e+03 3.129775700000000143e+03
3.158590200000000095e+03 3.174623700000000099e+03 3.712478599999999915e+03

27h

Geometry

H	2.5994570000	-1.0705070000	0.0000010000
H	1.1182570000	-1.3680320000	-0.8925770000
H	1.1182560000	-1.3680310000	0.8925770000
H	1.7492240000	1.0732760000	-0.8703400000
H	1.7492240000	1.0732760000	0.8703390000
H	-0.3153750000	2.1696660000	0.0000000000
H	-2.2860200000	0.9335120000	0.0000000000
H	-0.7190160000	-1.4084290000	-0.0000010000
C	1.5234830000	-0.8797760000	0.0000000000
C	1.2478790000	0.6298910000	0.0000000000
C	-0.1900250000	1.0906990000	0.0000000000
C	-1.3379010000	0.4077450000	0.0000000000
O	-1.5593280000	-0.9407600000	0.0000000000

Frequency

5.932119999999999749e+01 2.291706000000000074e+02 2.669085999999999785e+02
3.548179999999999836e+02 3.859827000000000226e+02 4.891965000000000146e+02
7.402926999999999680e+02 7.489112000000000080e+02 8.109230000000000018e+02
8.205451000000000477e+02 9.672336000000000240e+02 1.000839899999999943e+03
1.062185199999999895e+03 1.109322200000000066e+03 1.114608699999999999e+03
1.254981999999999971e+03 1.304239600000000110e+03 1.357586000000000013e+03
1.384233999999999924e+03 1.412279099999999971e+03 1.440859400000000051e+03
1.487366600000000062e+03 1.515222199999999930e+03 1.525460000000000036e+03
1.731826199999999972e+03 2.994239700000000084e+03 3.007128400000000056e+03
3.012649600000000191e+03 3.073333500000000186e+03 3.085881800000000112e+03
3.143076500000000124e+03 3.184618699999999990e+03 3.844846100000000206e+03

9.866320000000000334e+01 2.237554000000000087e+02 2.378474999999999966e+02
3.187803000000000111e+02 3.658405000000000200e+02 6.503388999999999669e+02
6.650003000000000384e+02 7.397136000000000422e+02 8.229072999999999638e+02
8.456316000000000486e+02 1.000216499999999996e+03 1.001978600000000029e+03
1.052240999999999985e+03 1.069350300000000061e+03 1.169672800000000052e+03

```
1.248529999999999973e+03 1.250702500000000100e+03 1.355283300000000054e+03
1.387721299999999928e+03 1.408082599999999957e+03 1.427481199999999944e+03
1.485044300000000021e+03 1.517263300000000072e+03 1.518708399999999983e+03
1.591485799999999927e+03 2.945874899999999798e+03 2.949252800000000207e+03
3.010444800000000214e+03 3.074648700000000190e+03 3.120169499999999971e+03
3.167676300000000083e+03 3.191897399999999834e+03 3.621337199999999939e+03
```

Table S20. Cartesian coordinates (Å), and harmonic frequencies (cm^{-1}) of isomer **25** (2-methylprop-1-en-1-ol) calculated at the composite CBS-QB3 level of theory.

25a

Geometry

H	-2.312691	-0.531598	-0.880171
H	-2.312691	-0.531599	0.880170
H	-1.551386	-1.863546	-0.000001
H	-1.052146	1.829625	0.880323
H	-1.052128	1.829627	-0.880333
H	0.463700	1.982926	0.000011
H	0.788957	-1.780357	0.000001
H	1.981087	0.775773	-0.000004
C	-0.402440	-0.030352	0.000000
C	-1.707841	-0.782431	0.000000
C	-0.504738	1.472925	0.000000
C	0.756470	-0.696748	0.000000
O	2.024824	-0.186402	0.000000

Frequency

7.995310000000000628e+01 1.941245000000000118e+02 2.798928999999999974e+02
2.868007000000000062e+02 3.380011999999999830e+02 3.656050000000000182e+02
4.887389000000000010e+02 5.757933000000000447e+02 8.036471999999999980e+02
8.857115000000000009e+02 9.742323999999999842e+02 1.014793400000000020e+03
1.041115800000000036e+03 1.096796499999999924e+03 1.173590899999999920e+03
1.201775699999999915e+03 1.313148100000000113e+03 1.405223600000000033e+03
1.416683199999999943e+03 1.428819300000000112e+03 1.468722299999999905e+03
1.485367600000000039e+03 1.496371499999999969e+03 1.504088999999999942e+03
1.743708900000000085e+03 2.996905499999999847e+03 3.003609199999999873e+03
3.034170000000000073e+03 3.046231099999999969e+03 3.064586699999999837e+03
3.091136800000000221e+03 3.182378499999999804e+03 3.818422799999999825e+03

7.820289999999999964e+01 1.223552999999999997e+02 1.916457000000000050e+02
2.823485000000000014e+02 3.816689999999999827e+02 4.014504999999999768e+02
5.684365999999999985e+02 6.602743000000000393e+02 7.974950999999999794e+02
8.958218000000000529e+02 9.576686999999999443e+02 9.722308000000000447e+02
1.031985799999999927e+03 1.039921599999999899e+03 1.200897999999999911e+03
1.276189699999999903e+03 1.353379500000000007e+03 1.383060500000000047e+03
1.388183399999999892e+03 1.399613000000000056e+03 1.432382299999999987e+03
1.443342799999999897e+03 1.481457599999999957e+03 1.509644700000000057e+03
1.599708000000000084e+03 2.982324599999999919e+03 2.992545099999999820e+03
3.043707699999999932e+03 3.067040700000000015e+03 3.111493599999999788e+03
3.136275399999999991e+03 3.193190000000000055e+03 3.687758800000000065e+03

25b

Geometry

H	-2.319826	-0.551036	0.879688

H	-1.547863	-1.877989	-0.000042
H	-2.319960	-0.550936	-0.879512
H	-1.054256	1.832539	0.879924
H	-1.054250	1.832548	-0.879976
H	0.480856	1.932627	-0.000008
H	0.804183	-1.755352	-0.000051
H	2.666919	-0.656199	-0.000503
C	-0.418701	-0.028798	-0.000037
C	-1.712663	-0.797493	0.000027
C	-0.507082	1.473143	-0.000026
C	0.750716	-0.669633	-0.000042
O	1.958823	-0.008689	0.000118

Frequency

1.006471999999999980e+02 1.662081000000000017e+02 2.125841000000000065e+02
2.665602000000000089e+02 3.030939000000000192e+02 3.599658999999999764e+02
4.538086000000000126e+02 5.817904999999999518e+02 8.146326000000000249e+02
8.468911000000000513e+02 9.740421999999999798e+02 1.013155600000000049e+03
1.043863000000000056e+03 1.101364600000000110e+03 1.168344000000000051e+03
1.195836000000000013e+03 1.312932299999999941e+03 1.386511999999999944e+03
1.413890300000000025e+03 1.424484699999999975e+03 1.473993300000000090e+03
1.487743199999999888e+03 1.495730199999999968e+03 1.503136199999999917e+03
1.766549500000000080e+03 2.997494200000000092e+03 3.004952600000000075e+03
3.035860999999999876e+03 3.040494700000000194e+03 3.084413300000000163e+03
3.124286399999999958e+03 3.138434400000000096e+03 3.876851999999999862e+03

7.571980000000000643e+01 1.249329000000000036e+02 2.060579999999999927e+02
2.822678999999999974e+02 3.812760999999999854e+02 4.217264999999999873e+02
5.797598000000000411e+02 6.270362999999999829e+02 8.069530999999999494e+02
8.889989000000000487e+02 9.195616999999999734e+02 9.691647000000000389e+02
1.030859500000000025e+03 1.039863299999999981e+03 1.210712700000000041e+03
1.289500000000000000e+03 1.346193999999999960e+03 1.361569199999999910e+03
1.377526699999999892e+03 1.399927400000000034e+03 1.433943999999999960e+03
1.441759299999999939e+03 1.478078199999999924e+03 1.501029899999999998e+03
1.620874499999999898e+03 2.976450899999999820e+03 2.991403699999999844e+03
3.045934600000000046e+03 3.081182800000000043e+03 3.133333999999999833e+03
3.146732700000000023e+03 3.165458099999999831e+03 3.728698400000000220e+03

SI References

(1) Marks, J. H.; Wang, J.; Fortenberry, R. C.; Kaiser, R. I. Preparation of methanediamine (CH2(NH2)2)—A precursor to nucleobases in the interstellar medium. *Proc. Natl. Acad. Sci. U. S. A.* **2022**, *119*, e2217329119. DOI: 10.1073/pnas.2217329119.

(2) Wang, J.; Marks, J. H.; Eckhardt, A. K.; Kaiser, R. I. Unconventional formation of cyanamide, a key intermediate in prebiotic chemical evolution, in interstellar ice analogues. *J. Phys. Chem. Lett.* **2026**, *17*, 2748–2753. DOI: 10.1021/acs.jpclett.6c00331.

(3) Wang, J.; Inada, S.; Zhang, C.; Marks, J. H.; Wang, Z.; Bergantini, A.; McAnally, M.; Eckhardt, A. K.; Kaiser, R. I. Abiotic formation of nitrile precursors to amino acids and nucleobases in interstellar ice analogues. *Chem. Sci.* **2026**, *17*, 4966–4977. DOI: 10.1039/D5SC08569A.

(4) Wang, J.; Marks, J. H.; Inada, S.; Kaiser, R. I. Bottom-up formation of phenol (C6H5OH) in interstellar analog ices of acetylene and water exposed to ionizing radiation. *Astrophys. J.* **2026**, *1000*, 76. DOI: 10.3847/1538-4357/ae48fa.

(5) Turner, A. M.; Abplanalp, M. J.; Chen, S. Y.; Chen, Y. T.; Chang, A. H. H.; Kaiser, R. I. A photoionization mass spectroscopic study on the formation of phosphanes in low temperature phosphine ices. *Phys. Chem. Chem. Phys.* **2015**, *17*, 27281–27291. DOI: 10.1039/C5CP02835C.

(6) Gerakines, P. A.; Materese, C. K.; Hudson, R. L. Carbon monoxide ices—A semicentennial review and update for crystalline CO along with the first IR spectrum and band strength for amorphous CO. *Mon. Not. R. Astron. Soc.* **2023**, *522*, 3145–3162. DOI: 10.1093/mnras/stad1164.

(7) Hudson, R. L.; Gerakines, P. A.; Yarnall, Y. Y.; Coones, R. T. Infrared spectra and optical constants of astronomical ices: III. Propane, propylene, and propyne. *Icarus* **2021**, *354*, 114033. DOI: 10.1016/j.icarus.2020.114033.

(8) Garozzo, M.; Fulvio, D.; Kanuchova, Z.; Palumbo, M. E.; Strazzulla, G. The fate of S-bearing species after ion irradiation of interstellar icy grain mantles. *Astron. Astrophys.* **2010**, *509*, A67. DOI: 10.1051/0004-6361/200913040.

(9) Hudson, R. L.; Gerakines, P. A.; Moore, M. H. Infrared spectra and optical constants of astronomical ices: II. Ethane and ethylene. *Icarus* **2014**, *243*, 148–157. DOI: 10.1016/j.icarus.2014.09.001.

(10) Hudson, R. L.; Coleman, F. M. Infrared intensities and molar refraction of amorphous dimethyl carbonate—Comparisons to four interstellar molecules. *Phys. Chem. Chem. Phys.* **2019**, *21*, 11284–11289. DOI: 10.1039/C9CP01709G.

(11) Hudson, R. L.; Ferrante, R. F. Quantifying acetaldehyde in astronomical ices and laboratory analogues: IR spectra, intensities, 13C shifts, and radiation chemistry. *Mon. Not. R. Astron. Soc.* **2020**, *492*, 283–293. DOI: 10.1093/mnras/stz3323.

(12) Yeghikyan, A. G. Irradiation of dust in molecular clouds. II. Doses produced by cosmic rays. *Astrophysics* **2011**, *54*, 87–99. DOI: 10.1007/s10511-011-9160-2.

(13) Abplanalp, M. J.; Kaiser, R. I. Complex hydrocarbon chemistry in interstellar and solar system ices revealed: A combined infrared spectroscopy and reflectron time-of-flight mass spectrometry analysis of ethane (C2H6) and D6-ethane (C2D6) ices exposed to ionizing radiation. *Astrophys. J.* **2016**, *827*, 132. DOI: 10.3847/0004-637X/827/2/132.

(14) Frisch, M. J.; Trucks, G. W.; Schlegel, H. B.; Scuseria, G. E.; Robb, M. A.; Cheeseman, J. R.; Scalmani, G.; Barone, V.; Mennucci, B.; Petersson, G. A.; Nakatsuji, H.; Caricato, M.; Li, X.; Hratchian, H. P.; Izmaylov, A. F.; Bloino, J.; Zheng, G.; Sonnenberg, J. L.; Hada, M.; Ehara, M.; Toyota, K.; Fukuda, R.; Hasegawa, J.; Ishida, M.; Nakajima, T.; Honda, Y.; Kitao, O.; Nakai, H.; Vreven, T.; Montgomery, J. A., Jr.; Peralta, J. E.; Ogliaro, F.; Bearpark, M.; Heyd, J. J.; Brothers, E.; Kudin, K. N.; Staroverov, V. N.; Keith, T.; Kobayashi, R.; Normand, J.; Raghavachari, K.; Rendell, A.; Burant, J. C.; Iyengar, S. S.; Tomasi, J.; Cossi, M.; Rega, N.; Millam, J. M.; Klene, M.; Knox, J. E.; Cross, J. B.; Bakken, V.; Adamo, C.; Jaramillo, J.; Gomperts, R.; Stratmann, R. E.; Yazyev, O.; Austin, A. J.; Cammi, R.; Pomelli, C.; Ochterski, J. W.; Martin, R. L.; Morokuma, K.; Zakrzewski, V. G.; Voth, G. A.; Salvador, P.; Dannenberg, J. J.; Dapprich, S.; Daniels, A. D.; Farkas, O.; Foresman, J. B.; Ortiz, J. V.; Cioslowski, J.; Fox, D. J. *Gaussian 09, Revision D.01*; Gaussian, Inc.: Wallingford, CT, **2013**.

(15) Montgomery, J. A.; Frisch, M. J.; Ochterski, J. W.; Petersson, G. A. A complete basis set model chemistry. VI. Use of density functional geometries and frequencies. *J. Chem. Phys.* **1999**, *110*, 2822–2827. DOI: 10.1063/1.477924.

(16) Zhou, J.; Fu, C.; Ma, S. Gold-catalyzed stereoselective cycloisomerization of allenoic acids for two types of common natural γ-butyrolactones. *Nat. Commun.* **2018**, *9*, 1654. DOI: 10.1038/s41467-018-03894-6.

(17) Hudson, R. L.; Loeffler, M. J.; Ferrante, R. F.; Gerakines, P. A.; Coleman, F. M. Testing densities and refractive indices of extraterrestrial ice components using molecular structures—Organic compounds and molar refractions. *Astrophys. J.* **2020**, *891*, 22. DOI: 10.3847/1538-4357/ab6efa.

(18) Bouilloud, M.; Fray, N.; Bénilan, Y.; Cottin, H.; Gazeau, M.-C.; Jolly, A. Bibliographic review and new measurements of the infrared band strengths of pure molecules at 25 K: H2O, CO2, CO, CH4, NH3, CH3OH, HCOOH and H2CO. *Mon. Not. R. Astron. Soc.* **2015**, *451*, 2145–2160. DOI: 10.1093/mnras/stv1021.

(19) Gough, K. M.; Murphy, W. F.; Raghavachari, K. The harmonic force field of propane. *J. Chem. Phys.* **1987**, *87*, 3332–3340. DOI: 10.1063/1.453027.

(20) Ghosh, J.; Hariharan, A. K.; Bhuin, R. G.; Methikkalam, R. R. J.; Pradeep, T. Propane and propane–water interactions: A study at cryogenic temperatures. *Phys. Chem. Chem. Phys.* **2018**, *20*, 1838–1847. DOI: 10.1039/C7CP06467E.

(21) Wang, J.; Marks, J. H.; Batrakova, E. A.; Tuchin, S. O.; Antonov, I. O.; Kaiser, R. I. Formation of methylglyoxal (CH3C(O)CHO) in interstellar analog ices—A key intermediate in cellular metabolism. *Phys. Chem. Chem. Phys.* **2024**, *26*, 23654–23662. DOI: 10.1039/D4CP02779E.

(22) Eckhardt, A. K.; Bergantini, A.; Singh, S. K.; Schreiner, P. R.; Kaiser, R. I. Formation of glyoxylic acid in interstellar ices: A key entry point for prebiotic chemistry. *Angew. Chem., Int. Ed.* **2019**, *58*, 5663–5667. DOI: 10.1002/anie.201901059.

(23) Pacansky, J.; Dupuis, M. Assignment of the infrared spectrum for the ethyl radical. *J. Am. Chem. Soc.* **1982**, *104*, 415–421. DOI: 10.1021/ja00366a007.

(24) Hepp, M.; Herman, M. The jet cooled spectrum of ethane between 4000 and 4500 cm−1. *Mol. Phys.* **1998**, *94*, 829–838. DOI: 10.1080/00268979809482376.

(25) Hepp, M.; Herman, M. Weak combination bands in the 3-μm region of ethane. *J. Mol. Spectrosc.* **1999**, *197*, 56–63. DOI: 10.1006/jmsp.1999.7893.

(26) Kim, S. J. Infrared excitation processes of C2H6 in comets. *Earth, Planets Space* **2003**, *55*, 139–151. DOI: 10.1186/BF03351741.

(27) Bennett, C. J.; Jamieson, C. S.; Osamura, Y.; Kaiser, R. I. Laboratory studies on the irradiation of methane in interstellar, cometary, and solar system ices. *Astrophys. J.* **2006**, *653*, 792–811. DOI: 10.1086/508561.

(28) Bennett, C. J.; Kaiser, R. I. The formation of acetic acid (CH3COOH) in interstellar ice analogs. *Astrophys. J.* **2007**, *660*, 1289–1295. DOI: 10.1086/513267.

(29) Kim, Y. S.; Bennett, C. J.; Chen, L.-H.; O'Brien, K.; Kaiser, R. I. Laboratory studies on the irradiation of solid ethane analog ices and implications to Titan's chemistry. *Astrophys. J.* **2010**, *711*, 744–756. DOI: 10.1088/0004-637X/711/2/744.

(30) Lattanzi, F.; Di Lauro, C.; Vander Auwera, J. Toward the understanding of the high resolution infrared spectrum of C2H6 near 3.3 μm. *J. Mol. Spectrosc.* **2011**, *267*, 71–79. DOI: 10.1016/j.jms.2011.02.003.

(31) Kaiser, R. I.; Maity, S.; Jones, B. M. Infrared and reflectron time-of-flight mass spectroscopic analysis of methane (CH4)–carbon monoxide (CO) ices exposed to ionization radiation—Toward the formation of carbonyl-bearing molecules in extraterrestrial ices. *Phys. Chem. Chem. Phys.* **2014**, *16*, 3399–3424. DOI: 10.1039/C3CP54255F.

(32) Wu, Y.-J.; Chuang, S.-J.; Chen, S.-C.; Huang, T.-P. Infrared spectra of acetylene diluted in solid nitrogen upon irradiation with vacuum ultraviolet light and electrons. *Astrophys. J., Suppl. Ser.* **2014**, *212*, 7. DOI: 10.1088/0067-0049/212/1/7.

(33) Crighton, J. S.; Bell, S. Structure and potential energy functions for acetaldehyde: *Ab initio* calculations of X̃1A′, Ã1A″, and B̃1A′ states. *J. Mol. Spectrosc.* **1985**, *112*, 285–303. DOI: 10.1016/0022-2852(85)90161-4.

(34) Kleimeier, N. F.; Kaiser, R. I. Interstellar enolization—Acetaldehyde (CH3CHO) and vinyl alcohol (H2CCH(OH)) as a case study. *ChemPhysChem* **2021**, *22*, 1229–1236. DOI: 10.1002/cphc.202100111.

(35) Wang, J.; Turner, A. M.; Marks, J. H.; Fortenberry, R. C.; Kaiser, R. I. Formation of paraldehyde (C6H12O3) in interstellar analog ices of acetaldehyde exposed to ionizing radiation. *ChemPhysChem* **2024**, *25*, e202400837. DOI: 10.1002/cphc.202400837.

(36) Jacox, M. E. The reaction of F atoms with acetaldehyde and ethylene oxide. Vibrational spectra of the CH3CO and CH2CHO free radicals trapped in solid argon. *Chem. Phys.* **1982**, *69*, 407–422. DOI: 10.1016/0301-0104(82)88079-8.

(37) Kleimeier, N. F.; Turner, A. M.; Fortenberry, R. C.; Kaiser, R. I. On the formation of the popcorn flavorant 2,3-butanedione (CH3COCOCH3) in acetaldehyde-containing interstellar ices. *ChemPhysChem* **2020**, *21*, 1531–1540. DOI: 10.1002/cphc.202000116.

(38) Hollenstein, H.; Günthard, Hs. H. Solid state and gas infrared spectra and normal coordinate analysis of 5 isotopic species of acetaldehyde. *Spectrochim. Acta*, *Part A* **1971**, *27*, 2027–2060. DOI: 10.1016/0584-8539(71)80102-2.

(39) Hudson, R. L.; Loeffler, M. J. Ketene formation in interstellar ices: A laboratory study. *Astrophys. J.* **2013**, *773*, 109. DOI: 10.1088/0004-637X/773/2/109.
